# TMEM232 is required for the formation of sperm flagellum and male fertility in mice

**DOI:** 10.1038/s41419-024-07200-9

**Published:** 2024-11-08

**Authors:** Xinying Cai, Hui Zhang, Shuai Kong, Weilong Xu, Jie Zheng, Ning Wang, Shuai He, Shupei Li, Yiru Shen, Ke Wang, Zengyunou Zhang, Haijian Cai, Fang Ma, Shun Bai, Fuxi Zhu, Fengli Xiao, Fengsong Wang

**Affiliations:** 1 Department of Dermatology, The First Affiliated Hospital of Anhui Medical University, Hefei, 230022 China; 2 Institute of Dermatology, Anhui Medical University, Hefei, Anhui China; 3 Key Laboratory of Dermatology, Anhui Medical University, Ministry of Education, Hefei, Anhui China; 4 School of Life Science, Anhui Medical University, Hefei, 230022 China; 5 NHC Key Laboratory of Study on Abnormal Gametes and Reproductive Tract (Anhui Medical University), Hefei, 230032 Anhui China; 6 The Center for Scientific Research of Anhui Medical University, Hefei Anhui, China; 7 Reproductive and Genetic Hospital, The First Affiliated Hospital of USTC, Division of Life Sciences and Medicine, University of Science and Technology of China, Hefei, Anhui 230001 China; 8 Reproductive Medicine Center, Department of Obstetrics and Gynecology, The Second Affiliated Hospital of Anhui Medical University, Hefei, 230601 China; 9 Reproductive Medicine Center, Department of Obstetrics and Gynecology, The First Affiliated Hospital of Anhui Medical University, Hefei, 230022 Anhui China; 10 Inflammation and Immune Mediated Diseases Laboratory of Anhui Province, Hefei, China

**Keywords:** Infertility, Protein-protein interaction networks, Infertility

## Abstract

Asthenoteratozoospermia is a major cause of male infertility. Thus far, the identified related genes can explain only a small share of asthenoteratozoospermia cases, suggesting the involvement of other genes. The transmembrane protein TMEM232 is highly expressed in mouse testes. In the present study, to determine its function of TMEM232 in testes, we constructed a *Tmem232*-null mouse model using CRISPR–Cas9 technology. *Tmem232* knockout (KO) male mice was completely infertile, and their sperm were immotile, with morphological defects of the flagellum. Electron microscopy revealed an aberrant midpiece-principal junction and the loss of the fourth outer microtubule doublet in the sperm of *Tmem232*^−/−^ mice. Sperm cells presented an 8 + 2 conformation and an irregular arrangement of the mitochondrial sheath. Proteomic analysis revealed altered expression of proteins related to flagellar motility, sperm capacitation, the integrity and stability of sperm structure, especially an upregulated expression of multiple ribosome components in TMEM232-deficient spermatids. Additionally, TMEM232 was observed to be involved in autophagy by interacting with autophagy-related proteins, such as ATG14, to regulate ribosome homeostasis during spermiogenesis. These results suggest that TMEM232, as a potential scaffold protein involving in the correct assembly, distribution, and stability maintenance of certain functional complexes by recruiting key intracellular proteins, is essential for the formation of a highly structured flagellum and plays an important role in the autophagic elimination of cytosolic ribosomes to provide energy for sperm motility.

## Introduction

Spermatozoa have highly specialized structural characteristics, with normal morphology and structural integrity critical for male fertility and complete fertilization [1]. A mature sperm comprises a head and a long flagellum connected by a neck. The normal sperm flagellum contains a midpiece, a principal piece, and an endpiece, with the axoneme running throughout the length. The annulus, a SEPTIN-based electron-densering structure, forms a diffusion barrier between the midpiece and principal piece of sperm to maintain the orderly arrangement of the mitochondrial sheath (MS). The axoneme comprises nine outer microtubular doublets (MTDs) and a central pair of microtubules, resulting in the classical 9 + 2 microtubular arrangement [1]. Attached to each of the nine MTDs, the regularly repeating hook-shaped structures of outer dynein arms and inner dynein arms exhibit ATPase activity, providing the sliding force for motility [2]. The axoneme is surrounded by accessory structures such as the outer dense fiber, fibrous sheath, and MS, which are specific to the sperm flagellum [3]. Any alterations within these structures can cause abnormal sperm morphology, reducing sperm motility and fertilization capacity.

Asthenoteratozoospermia, a major cause of male infertility, is characterized by malformed sperm with motility defects [4, 5]. According to the World Health Organization classification, sperm can be divided into progressive motile (PR), non-progressive motile (NP), and immotile types. Asthenoteratozoospermia is diagnosed when total motility (PR + NP) is <40%, progressive motility (PR) is <32%, and the normal sperm form represents 4% [6]. Based on the abnormal characteristics of sperm morphology, asthenoteratozoospermia can be divided into several types, including multiple morphological abnormalities of the sperm flagella (MMAF), oligo- or teratozoospermia, primary ciliary dyskinesia (PCD), and others. MMAF is a severe subtype of asthenoteratozoospermia, characterized by absent, bent, short, coiled, or irregular-caliber flagella. PCD is a genetically heterogeneous disorder characterized by chronic airway disease, left–right laterality disturbances, and male infertility [7]. As sperm flagella and motile cilia share an evolutionarily conserved axonemal structure, male infertility caused by malformations of the axonemal structure is often associated with PCD. To date, over 20 MMAF- and 40 PCD-associated genes, respectively, have been identified [8, 9]. However, these explain a small share of cases of asthenoteratozoospermia, indicating the potential involvement of other genetic factors.

Spermatogenesis is a complex process involving mitotic, meiotic, and post-meiotic phases for the formation of sperm [10]. The morphological and functional transformation from round spermatids into elongating mature spermatozoa is crucial in the late stage of spermatogenesis [11]. This stage is characterized by acrosome biogenesis, chromatin condensation, MS formation, sperm flagellum assembly, and removal of excess cytoplasm and organelles [11–13]. The removal of cytoplasm organelles is essential for mature sperm formation, and variations in this process can result in aberrant sperm phenotypes [13, 14]. Autophagy facilitates the intracellular degradation of dysfunctional or unnecessary proteins and organelles to maintain intracellular metabolism and energy homeostasis [15, 16]. In mice, the selective autophagic removal of ribosomes during the transition from round spermatids into elongated sperm was found to provide energy resources for the mitochondria in motile spermatids [17].

*TMEM232* is a highly conserved gene among mammals. In humans, *TMEM232* is localized on chromosome 5, contains 14 exons, and encodes a 657-amino-acid protein. Furthermore, *TMEM232* has been reported as a susceptibility gene for atopic dermatitis in the Chinese Han population [18], which was also confirmed in the Japanese and Korean populations [19]. To investigate the role of TMEM232 in atopic dermatitis, we generated *Tmem232* knockout (KO) mice [20]. Interestingly, the *Tmem232* KO mice were infertile and had a typical phenotype of asthenozoospermia. In this study, we aimed to elucidate the function of TMEM232 protein and the potential involvement of *Tmem232* gene in asthenoteratozoospermia. This mouse model, *Tmem232* KO, was generated to investigate its potential pathogenic mechanism and molecular function. We took observation of the sperm phenotype of *Tmem232* KO mice and conducted proteomic analysis of differential expression between *Tmem232*^*−/−*^ and *Tmem232*^*+/+*^ mouse sperm to reveal the proteins potentially responsible for phenotypic defects in sperm. In vivo and in vitro experiment results indicated that TMEM232 involved in the regulation of microtubule dynamics and fine structure assembly of sperm by interacting with ATAT1 (Alpha α-Tubulin Acetyltransferase 1) and SEPTINs, and also played an important role in regulating autophagy, affecting the selective elimination of cytosolic ribosomes during spermiogenesis, thereby supplying energy for flagellar motility. Our findings provide comprehensive experimental evidence for the function and important role of TMEM232 in sperm motility and flagellum development and highlight the pathological basis of asthenozoospermia, with implications for its clinical diagnosis and treatment.

## Results

### TMEM232 is an evolutionarily conserved testis-enriched protein

The Ensembl database (http://asia.ensembl.org/index.html) indicates that TMEM232 is highly conserved in mammals. Human TMEM232 shares 99.09, 95.13, 77.66, 66.01, and 65.49% protein sequence identity with its orthologs in macaques, chimpanzees, dogs, rats, and mice, respectively (Supplementary Fig. S1A, S1B). A long internal N-terminus, two potential transmembrane domains, and a long internal C-terminus were predicted using bioinformatics analyzes (https://www.uniprot.org/). To further explore the biological function of TMEM232, we generated an antibody that recognizes the 302–381 aa epitope of TMEM232. The specificity of the antibody was validated using a GST-TMEM232 fusion protein (Supplementary Fig. S1C).

**Fig. 1:**
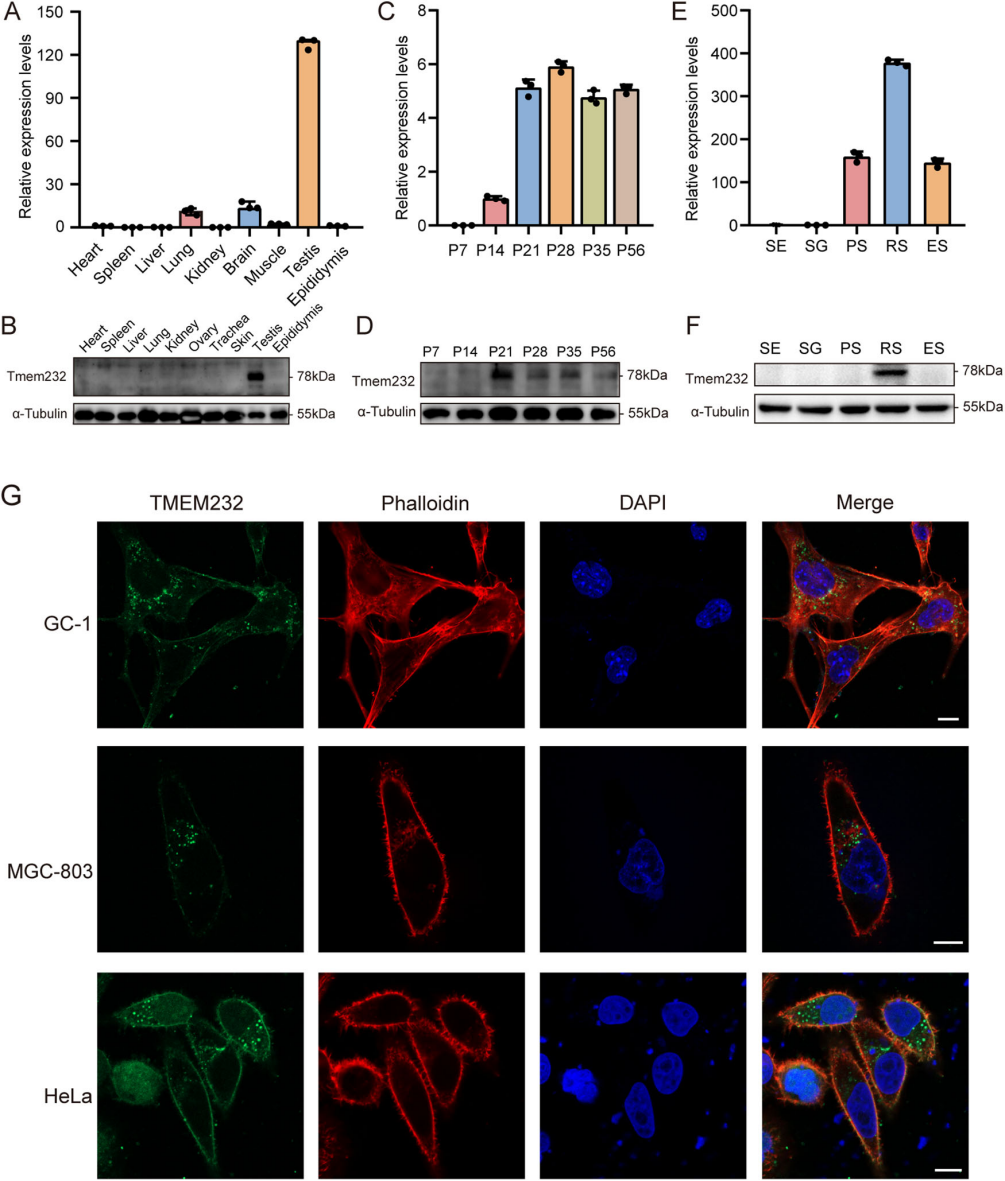
TMEM232 is an evolutionarily conserved testis-enriched protein. **A** RT-qPCR and **B** western blotting analysis of TMEM232 expression in multiple tissues from mice. The representative image of biological duplicates is shown. **C** RT-qPCR and **D** western blot analysis of TMEM232 in mouse testes on various postnatal days. The image represents two independent experiments. **E** RT-qPCR analysis of *Tmem232* in different fractions of spermatogenic cells. PS (pachytene spermatocytes), RS (round spermatids) and ES (elongating spermatids) were from mice at 2-months-old (*n* = 6), SG (spermatogonia) and SE (somatic Sertoli cells) were isolated from mice at postnatal day 6 using the STA-PUT method (*n* = 18). **F** The protein amount of TMEM232 in different fractions of spermatogenic cells. The image is a representation of two independent experiments with similar results. GADPH and α-Tubulin served as loading controls for RT-qPCR and western blotting, respectively. *n* = 3 for each sample. **G** Immunofluorescent staining of TMEM232 (green), phalloidin (F-actin, red), and DAPI (Nucleus, blue). GC-1, MGC-803, and HeLa cells were transfected with a *pEGFP-N2-TMEM232 plasmid*. Scale bar: 10 µm.

To determine the expression profile of TMEM232, RT-qPCR and western blotting experiments were performed using multiple tissues from adult mice. TMEM232 showed higher expression in the testis than in the other tissues (Fig. 1A, B). To gain insight into the temporal expression of TMEM232 in the testes, we isolated testes from postnatal day 7 (P7, stem cell, spermatogonia), P14 (late, pachytene, spermatocytes), P21 (early, round, spermatids), P28, P35, and P56 mice for RT-qPCR and western blotting experiments. Of these, P28, P35 and P56 mouse testes contained mature elongated spermatids, with one complete wave or multiple waves of spermatogenesis. Furthermore, *Tmem232* was significantly expressed in P14, peaked at P21, and maintained stable expression levels thereafter (Fig. 1C, D). To better understand the expression of *Tmem232* at different stages of mouse germ cell development, we performed STA-PUT velocity sedimentation to isolate elongating spermatids, round spermatids, pachytene spermatocytes, spermatogonia, and sertoli cells. The expression profile determined by RT-qPCR indicated that *Tmem232* was absent in spermatogonia, originally produced in pachytene spermatocytes, predominantly expressed in round spermatids, and reduced in elongated spermatids (Fig. 1E). Western blotting analysis revealed that the TMEM232 protein was highly enriched in round spermatids (Fig. 1F). This result is consistent with the localization of TMEM232 in human germ cells of testis [21], with high expression only in round spermatids. To determine the subcellular location of TMEM232 within cells, we constructed a green fluorescent protein (GFP)-tagged plasmid encoding TMEM232-GFP fusion protein and performed transient transfection assays in cell lines, including GC-1, MGC803, and HeLa. TMEM232-GFP fusion protein was located exclusively on the plasma membrane and some vesicles (Fig. 1G). These results indicated that TMEM232 is a testis-enriched protein with functional conservation and predicted spatiotemporal cross-species during spermatogenesis.

### *Tmem232* KO induces male infertility with significantly reduced sperm count and motility

**Fig. 2:**
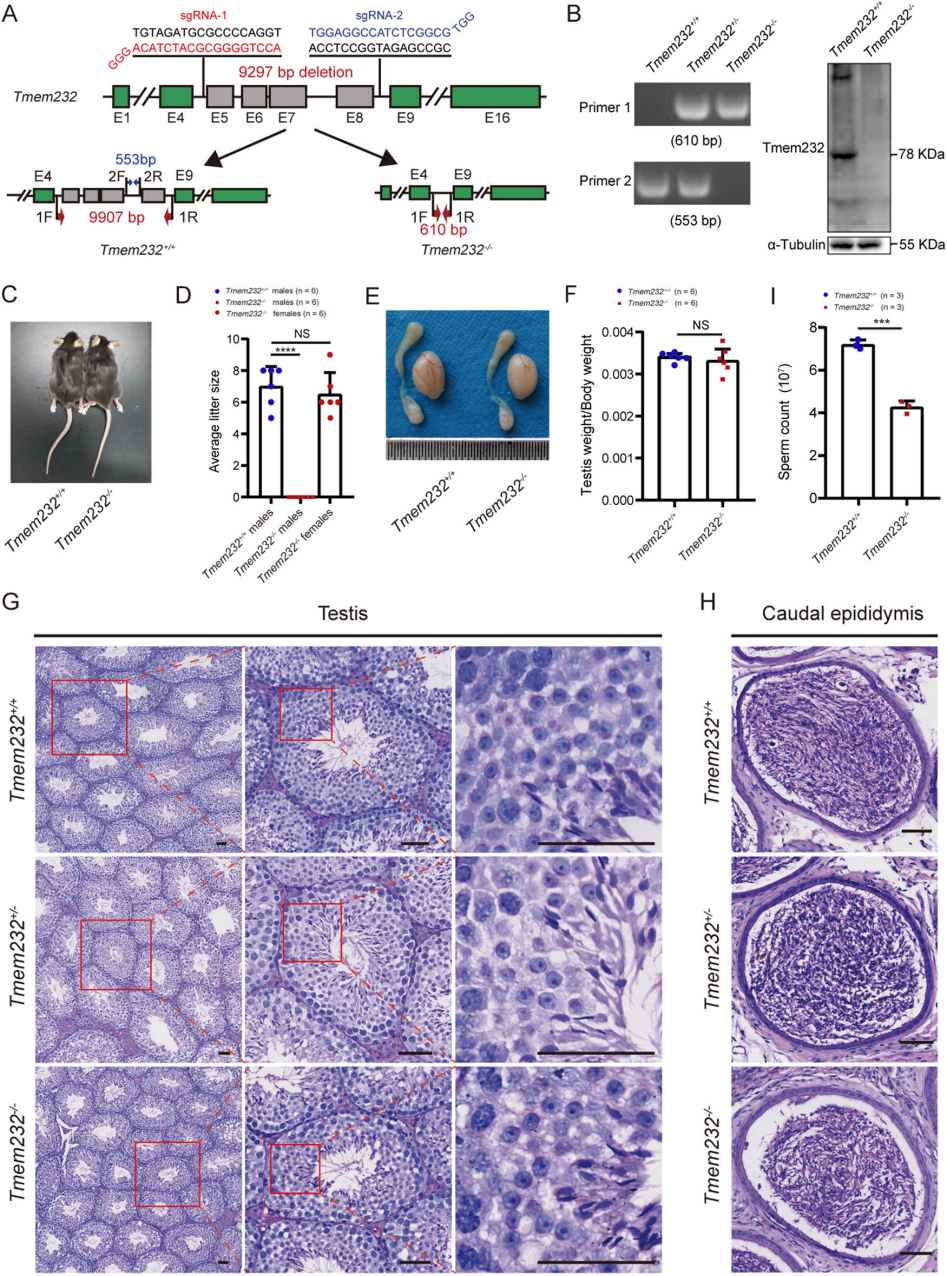
Knockout of *Tmem232* induces male infertility. **A** Schematic of the generation of *Tmem232* KO mice using CRISPR-Cas9. Two sgRNAs, sgRNA-1 and sgRNA-2, were constructed to target sequences in introns 4 and 8, respectively. The lower panel shows the location of primers for mouse genotyping at the *Tmem232* gene locus and the PCR product size. **B** Electrophoresis of PCR products amplified from total genomic DNA from the tails of mice with different genotypes (*Tmem232*^*+/+*^*, Tmem232*^*+/−*^, and *Tmem232*^*−/−*^) (left panel). The right panel shows Tmem232 levels in *Tmem232*^*+/+*^ and *Tmem232*^*−/−*^ mouse testis. **C** No significant differences in appearance were observed between *Tmem232*^*−/−*^ and wild-type mice. **D** The average litter size of *Tmem232*^+/+^ (*n* = 6), *Tmem232*^−/−^ males (*n* = 6), and *Tmem232*^−/−^ females (*n* = 6). *Tmem232*^−/−^ male and *Tmem232*^−/−^ female mice were bred with *Tmem232*^+/+^ mice, and the average litter size was noted. The *Tmem232*^−/−^ males were sterile. *Tmem232*^*+/+*^ males (7.00 ± 0.52), *Tmem232*^−/−^ males (0 ± 0), and *Tmem232*^−/−^ females (6.50 ± 0.56). Data are presented as mean ± SEM. **** indicates *P* < 0.0001. NS indicates that the value is not significant. *n* = 6 for each genotype. **E** Testis sizes were comparable between *Tmem232*^+/+^ and *Tmem232*^−/−^ mice at 8 weeks of age. **F** Ratio of testis weight/body weight in *Tmem232*^*+/+*^ and *Tmem232*^*−/−*^ male mice at 8 weeks of age. *Tmem232*^+/+^ (3.40 ± 0.04 ×10^−3^), *Tmem232*^−/−^ (3.33 ± 0.12 ×10^−3^). Data are presented as the mean ± SEM. NS indicates no significance. *n* = 6 for each genotype. Representative PAS staining images of testes (**G**) and caudal epididymis (**H**) of adult *Tmem232*^*+/+*^, *Tmem232*^*+/−*^, and *Tmem232*^*−/−*^ mice. Histomorphology of seminiferous tubules and acrosome morphology in different steps of spermatid development were normal in *Tmem232*^−/−^ mice. Sperm count in the *Tmem232*^*−/−*^ caudal epididymis was lower compared with that of the *Tmem232*^+/+^ and *Tmem232*^+/−^ caudal epididymis. Scale bar: 50 µm. **I** Fewer sperm were observed in the *Tmem232*^*−/−*^ (4.23 ± 0.17) caudal epididymis than that in *Tmem232*^+/+^ (7.20 ± 0.13) caudal epididymis. Data are presented as the mean ± SEM. *** indicates *P* < 0.001. *n* = 3 for each genotype.

To examine the functions of TMEM232 in vivo, we constructed a *Tmem232*-null (*Tmem232*^*−/−*^) mouse model by deleting a 9297-bp fragment of *Tmem232* gene using CRISPR-Cas9-mediated genome editing (Fig. 2A). This deletion induced a truncated protein with only 39 amino acid residues. TMEM232 protein was absent in *Tmem232*^*−/−*^ testes (Fig. 2B). No significant differences in appearance (Fig. 2C), behavior (including sexual behavior), or survival were observed between *Tmem232*^*−/−*^ and wild-type mice. Unexpectedly, *Tmem232*^*−/−*^ male mice were infertile, whereas *Tmem232* deletion did not affect the fertility of female mice (Fig. 2D).

To explore the cause of male infertility, we investigated the gross anatomic and histological properties of *Tmem232*^*−/−*^ male mice. The testes size, weight, and ratio of *Tmem232*^*−/−*^ mice were indistinguishable from those of wild-type mice (Fig. 2E, F). Furthermore, no morphological differences were observed in testicular and ovarian tissue sections using periodic acid-Schiff (PAS) staining and hematoxylin and eosin (H&E), respectively (Fig. 2G and Supplementary Fig. S2). Analysis of the caudal epididymal sections revealed a reduced sperm count in *Tmem232*^*−/−*^ male mice than in their wild-type counterparts (Fig. 2H). Furthermore, the total sperm count in the caudal epididymis was markedly reduced in *Tmem232*^*−/−*^ mice (Fig. 2I). A computer-assisted sperm analyzer was used to examine the sperm quality of *Tmem232*^*−/−*^ mice comprehensively (Supplementary Table S1). Interestingly, almost no motile or progressive sperm was discerned in *Tmem232*^*−/−*^ mice as opposed to in *Tmem232*^*+/+*^ mice (Supplementary Movies 1 and 2). After incubation under capacitating conditions for 10 min, *Tmem232*^*+/+*^ sperm flagella bent with a high amplitude, whereas *Tmem232*^*−/−*^ sperm remained immobile (Supplementary Movies 3 and 4). Collectively, these data indicate that TMEM232 is involved in sperm flagellar motility and that its abnormality may further lead to male infertility.

### *Tmem232*^*−/−*^ mouse sperm exhibit aberrant midpiece-principal piece junction

**Fig. 3:**
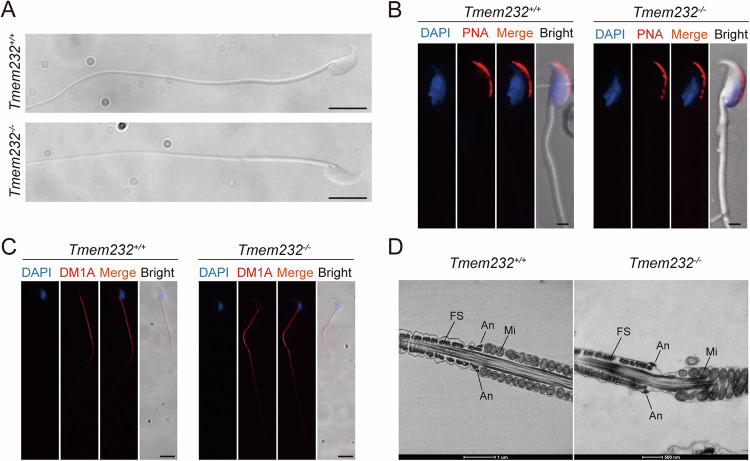
*Tmem232* deficiency disrupts the midpiece-principal piece junction in mice sperm. **A** Morphology of mouse sperm obtained from the caudal epididymes. *Tmem232*^−/−^ sperm flagella showed a gap between the midpiece and principal piece. Magnification: 20×, scale bars: 5 µm. No change was observed in the expression of PNA (**B**) and DM1A (**C**) between *Tmem232*^*−/−*^ and *Tmem232*^*+/+*^ mouse sperm. (red, detected protein, blue, DAPI; scale bar: 1 µm). **D** Transmission electron microscopy (TEM) analysis revealed that the annulus of *Tmem232*^*−/−*^ mouse sperm lost contact with the MS; However, this was not the case for *Tmem232*^*+/+*^ mouse sperm. An annulus, FS fibrous sheath, Mi mitochondrion; Scale bar: 1 µm or 500 nm.

The integrity of sperm structure is necessary for its motility and male fertility. Light microscopy revealed a gap between the principal piece and midpiece in the sperm flagella of *Tmem232*^*−/−*^ mice, whereas the sperm head was normal (Fig. 3A). Subsequent single sperm immunofluorescence staining of peanut agglutinin (PNA) and DM1A (α-Tubulin), the major components of microtubules, confirmed the normal acrosome structure and continuous axoneme of *Tmem232*^*−/−*^ mouse sperm (Fig. 3B, C). The surface structure of sperm flagella was further characterized by scanning electron microscopy (SEM) imaging, which clearly demonstrated abnormal types of sperm flagella and a normal hooked form of the sperm head in *Tmem232*^*−/−*^ mice (Supplementary Fig. S3A). Furthermore, transmission electron microscopy (TEM) analyses of spermatozoa isolated from the caudal epididymis were conducted to characterize the flagellum in *Tmem232*^*−/−*^ mouse sperm. The annulus, a ring-like cytoskeletal structure, was identified at the beginning of the principal piece, which had lost contact with the MS (Fig. 3D).

**Fig. 4:**
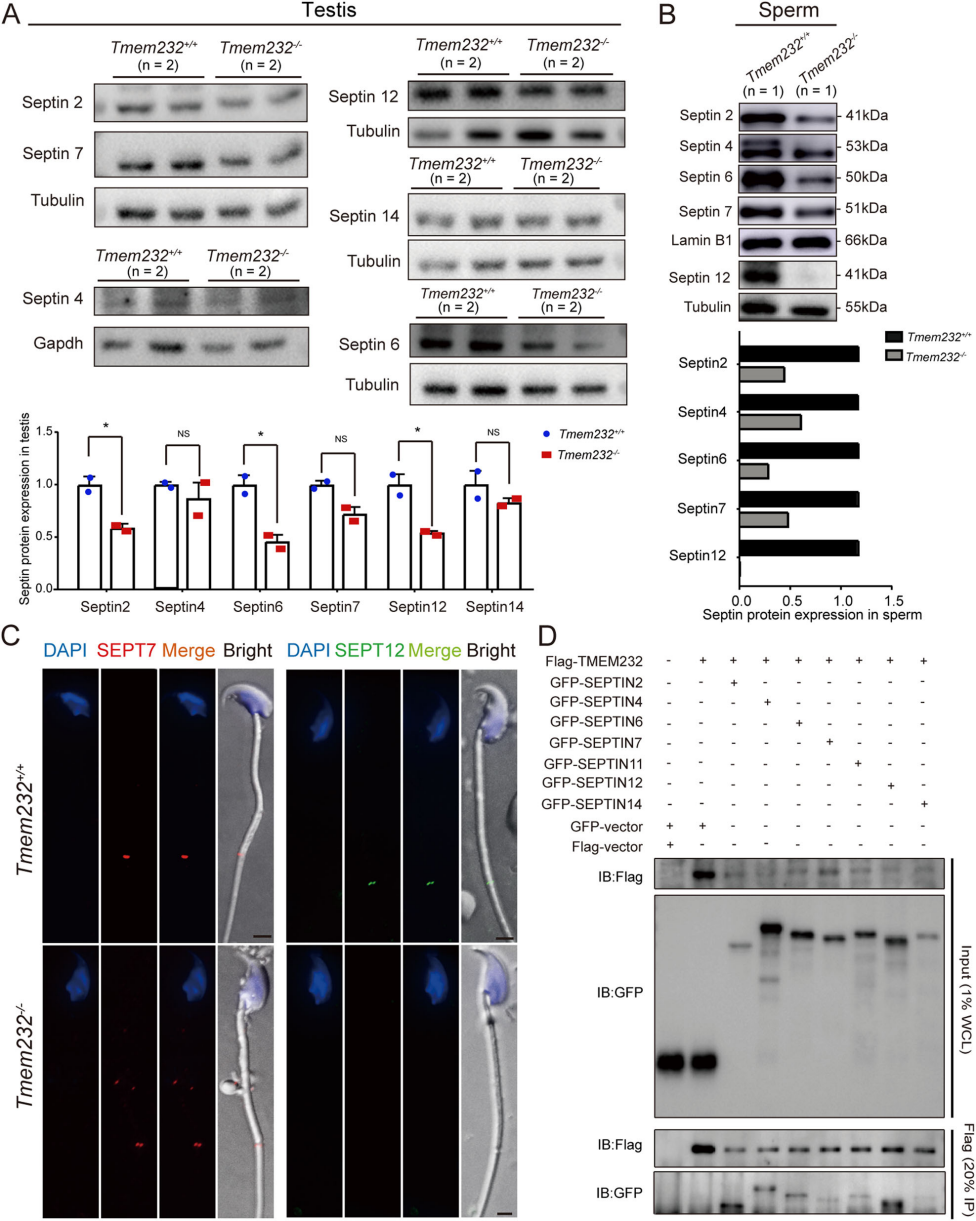
Expression of sperm annulus proteins is dysregulated in *Tmem232*^*−/−*^ mouse testis. The reduced protein expression of SEPTIN 2, 4, 6, 7, and 12 was detected in the testis (**A**) or spermatozoa (**B**) of *Tmem232*^*−/−*^ mice via western blotting. The corresponding optical density readings for each image are shown below. α-Tubulin and lamin-B1 served as loading controls. *n* = 2 for each genotype of testis tissue. The representative image of biological duplicates is shown. **C** The expression of SEPTIN 7 (left) and SEPTIN 12 (right) in *Tmem232*^*+/+*^and *Tmem232*^*−/−*^ mouse sperm was detected via immunofluorescence staining. **D** Immunoprecipitation experiments were performed using FLAG-M2 beads and the lysates of HEK293T cells which were co-transfected with GFP-SEPTIN2, GFP-SEPTIN4, GFP-SEPTIN6, GFP-SEPTIN7, GFP-SEPTIN11, GFP-SEPTIN12, GFP-SEPTIN14, and FLAG-TMEM232. The isolated proteins were then analyzed via western blotting with anti-FLAG and anti-GFP antibodies. The representative image of biological duplicates is shown.

Sperm annulus is a SEPTIN-based structure composed of members of the SEPTIN family 1, 2, 4, 6, 7, 12, et al. [22]. To explore whether the absence of *Tmem232* could affect the assembly of SEPTIN filaments, we determined the expression patterns of SEPTIN family proteins. Western blotting confirmed decreased expression of SEPTIN 2, 6, and 12 in *Tmem232*^*−/−*^ mouse testes (Fig. 4A), along with the downregulated expression of SEPTIN 2, 4, 6, 7, and 12 in the sperm of *Tmem232*^*−/−*^ mice (Fig. 4B). The expression of SEPTIN 2 and 6 was consistent with their transcriptional levels in the testes (Supplementary Fig. S3B). Meanwhile, immunofluorescence staining of sperm revealed the complete absence of the SEPTIN 12 signal, consistent with the results of western blotting. SEPTIN 7 was slightly decreased in *Tmem232*^*−/−*^ than in wild-type mice (Fig. 4C). Co-immunoprecipitation assay showed that TMEM232 could interact with SEPTIN 2, 4, 6, 7, 11, 12 and 14, especially with SEPTIN 2 and 12 (Fig. 4D). In Hela cells, TMEM232 was partly colocalized in some vesicles with septins (Supplementary Fig. S4). These results suggest that TMEM232 plays an important role in the formation of annulus-MS junction structure in mice.

### Tmem232 is required for the proper assembly of sperm flagellum axoneme

**Fig. 5:**
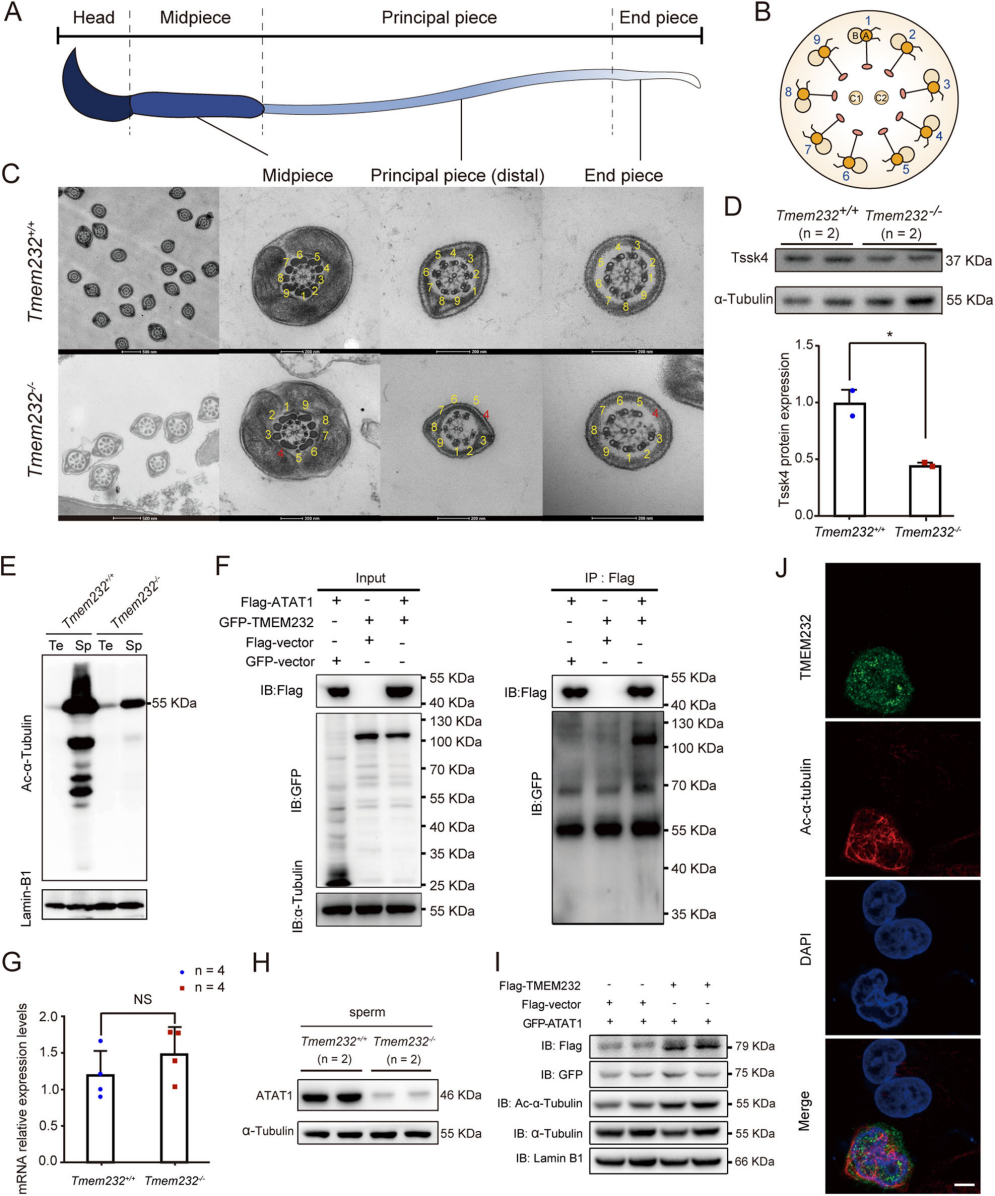
*Tmem232* deficiency leads to axonemal microtubule defects in mouse sperm flagella and decreased sperm motility. Schematic diagram showing a full-length sperm (**A**) and a cross-sectional view of the sperm axoneme (**B**). **C** Cross-sections showing the ultrastructure of the midpiece, principal piece, and endpiece of *Tmem232*^*+/+*^ and *Tmem232*^*−/−*^ mouse spermatozoa. Note that the *Tmem232*^*+/+*^ flagellum comprises a typical 9 + 2 arrangement of axonemal microtubules, whereas the *Tmem232*^*−/−*^ flagellum contains an atypical 8 + 2 composition of axonemal microtubules in the midpiece, principal piece, and endpiece. The red numbers indicate the missing MTDs of sperm flagella. Scale bars: 100 or 200 nm. **D** Western blotting of TSSK4 expression in *Tmem232*^*−/−*^ and *Tmem232*^*+/+*^ mouse testes. Western blotting bands quantified using Image J software are shown below. The α-Tubulin served as a loading control. The representative image of biological duplicates is shown. **E** Western blotting analysis for the comparison of Ac-α-Tubulin protein levels between *Tmem232*^*−/−*^ and *Tmem232*^*+/+*^ mice testis and sperm. Lamin-B1 served as a loading control. Te: testis; Sp: sperm. The representative image of biological duplicates is shown. **F** The co-immunoprecipitation assays indicated the interaction between mouse TMEM232 and ATAT1 in cells. Anti-Flag beads was used for immunoprecipitation. Anti-Flag and anti-GFP antibodies were used for western blotting analysis. The representative image of biological duplicates is shown. **G** RNA-sequencing revealed that ATAT1 mRNA expression was no significant difference between *Tmem232*^*−/−*^ (*n* = 4) and *Tmem232*^*+/+*^ (*n* = 4) mice testis. **H** The ATAT1 protein was downregulated in *Tmem232*^*−/−*^ (*n* = 2) mice sperm compared with that in *Tmem232*^*+/+*^ (*n* = 2) mice sperm. **I** Overexpression of Flag-TMEM232 promoted ATAT1 expression and α-Tubulin protein acetylation in cultured HeLa cells co-transfected with *pGFP-ATAT1* and *p-TMEM232×3FLAG-Myc-CMV-24* or *p×3FLAG-Myc-CMV-24* plasmid for 24 h. Cells were harvested for western blotting analysis with indicated antibodies. The results shown are representative of three independent experiments. The representative image of biological duplicates is shown. **J** Immunofluorescence staining of TMEM232 (green), Ac-α-Tubulin (red), and DAPI (nucleus, blue). HeLa cells were transfected with a *pEGFP-N2-TMEM232* plasmid. Scale bar: 5 µm.

The beating of sperm flagella and cilia depends on energy consumption and the integrated axonemal structure, which functions as a precise locomotor device. To elucidate the cause of immobility in *Tmem232*^*−/−*^ mouse sperm, we examined the ultrastructure of sperm flagella. TEM analysis of axonemal structure of *Tmem232*^*−/−*^ mouse sperm indicated the abnormal flagellum. Normally, the sperm flagellum displays a 9 + 2 axonemal configuration. In contrast, the *Tmem232-*null sperm flagellum exhibited a high-frequency deletion of MTD 4 at the midpiece, principal piece, and endpiece in all cross-sections, presenting an 8 + 2 conformation (Fig. 5A–C). The outer dynein arms and radial spokes (RS) attached to each of the remaining MTDs were observed. Considering that motile cilia and sperm flagella share common characteristics of axonemal structure, we additionally assessed the respiratory cilia of *Tmem232*^*−/−*^ mice. TEM analyses of motile cilia on the surface of tracheal epithelial cells demonstrated a normal 9 + 2 structure with coordinated directionality of the axonemes (Supplementary Fig. S5A). Furthermore, SEM analyses revealed that the cilia were shorter and fewer in *Tmem232*^*−/−*^ tracheal epithelium cells than in those of wild-type mice (Supplementary Fig. S5B).

To uncover the molecular mechanism underlying this variation, we first detected the expression of Tssk4, the absence of which led to a lack of axonemal MTDs and fusion of the sperm midpiece and principal piece. Western blotting of mouse testes showed that Tssk4 expression was decreased in *Tmem*232^−/−^ mice (Fig. 5D). Interestingly, α-Tubulin acetylation, which is implicated in regulating flagellar microtubule stability and function, was significantly reduced in *Tmem*232^−/−^ mice sperm compared to that in wild-type individuals (Fig. 5E). Previous studies have shown that α-Tubulin N-acetyltransferase 1 (ATAT1) is responsible for the acetylation of α-Tubulin [23, 24]. Our immunoprecipitation results showed that TMEM232 interacted with ATAT1 (Fig. 5F). TMEM232 was predicted to be a potential transmembrane proteins, and its 189-352 amino acid region between two transmembrane domains mainly mediatedly the interaction with ATAT1 (Supplementary Fig. S6A–C). The transmembrane region was necessary for the correct localization of TMEM232 (Supplementary Fig. S6D). RT-qPCR result indicated no significant difference in transcription level of *Atat1* in *Tmem232*^*−/−*^ and *Tmem232*^*+/+*^ mice testis (Fig. 5G, Supplementary Table S2), but ATAT1 was downregulated in protein level at *Tmem232*^*−/−*^ mice sperm (Fig. 5H). Overexpression of TMEM232 in HeLa cells led to an upregulation in the expression of the ATAT1 protein and an increase in the acetylation of α-Tubulin (Fig. 5I, J). Therefore, TMEM232 is necessary for the assembly of the axoneme complex and the stability of sperm flagella and respiratory cilia. TMEM232 can affect the α-Tubulin acetylation of sperm flagella-Tubulin through its interaction with ATAT1, thus modulating the stability of double sperm microtubules.

### Proteomic profiling of sperm from *Tmem232* KO mice

To elucidate the molecular mechanisms through which *Tmem232* KO aggravates abnormal sperm morphology, we conducted proteomic analyses of sperm from three *Tmem*232^−/−^ adult mice and three wild-type individuals. A total of 5199 proteins were identified, of which 4380 were quantified. The repeatability of quantified proteins was further compared by principal component analysis (PCA), and the samples were divided into two major categories (Supplementary Fig. S7A). A total of 343 DEPs were identified, including 88 upregulated and 255 downregulated (fold change ≥1.5, *P* < 0.05), with group comparisons presented in a volcano plot (Supplementary Fig. S7B, Supplementary Table S3). Proteins involved in cytoskeletal components, including the axoneme, annulus, outer dense fiber, MS, fibrous sheath, and calcium ion channels, as well as those implicated in energy metabolism, including the citrate cycle, glycolysis, and oxidative phosphorylation, were remarkably downregulated. To verify the proteomics results, we selected several significantly downregulated proteins for further investigation. Western blotting confirmed the decreased expression of structural proteins localized in the sperm flagellum (ODF1 and ODF2) and energy metabolism-associated proteins (PDHA2 and COX6B2) in *Tmem*232^−/−^ sperm (Supplementary Fig. S7C).

We then performed a KEGG enrichment analysis on these DEPs and identified 13 most enriched pathways (Supplementary Fig. S7D). These proteins were enriched in the citrate cycle, oxidative phosphorylation, carbon metabolism, and other pathways involved in energy metabolism, thus influencing sperm metabolism, motility, and survival. These enriched signaling pathways exhibited a high similarity with the enrichment pathways of downregulated proteins (Supplementary Fig. S8A). Next, a PPI network of DEPs was constructed using STRING v.11.5. The top 12 vital proteins, namely SOD2, GPX4, TXNRD3, NME8, RPL23, DNAIC1, COX6A1, SPAG6, COX4I1, COX5A, CCDC114, and WDR16, were determined using cytoNCA (Supplementary Fig. S7E). These proteins are important for maintaining the structural integrity of the central apparatus of the sperm tail, flagellar motility, and energy metabolism. Consistent results were obtained through GO analysis of differentially abundant proteins, which revealed significant enrichment in 30 terms, including 8, 8, and 14 terms of biological processes, cellular components, and molecular function, respectively (Supplementary Fig. S7F and Supplementary Table S4). Most of these terms were involved in germ cell development, including sperm flagellum, flagellated sperm motility, formation of microtubule bundles, motor activity of the microtubule, and pyruvate dehydrogenase activity, among others [25].

**Fig. 6:**
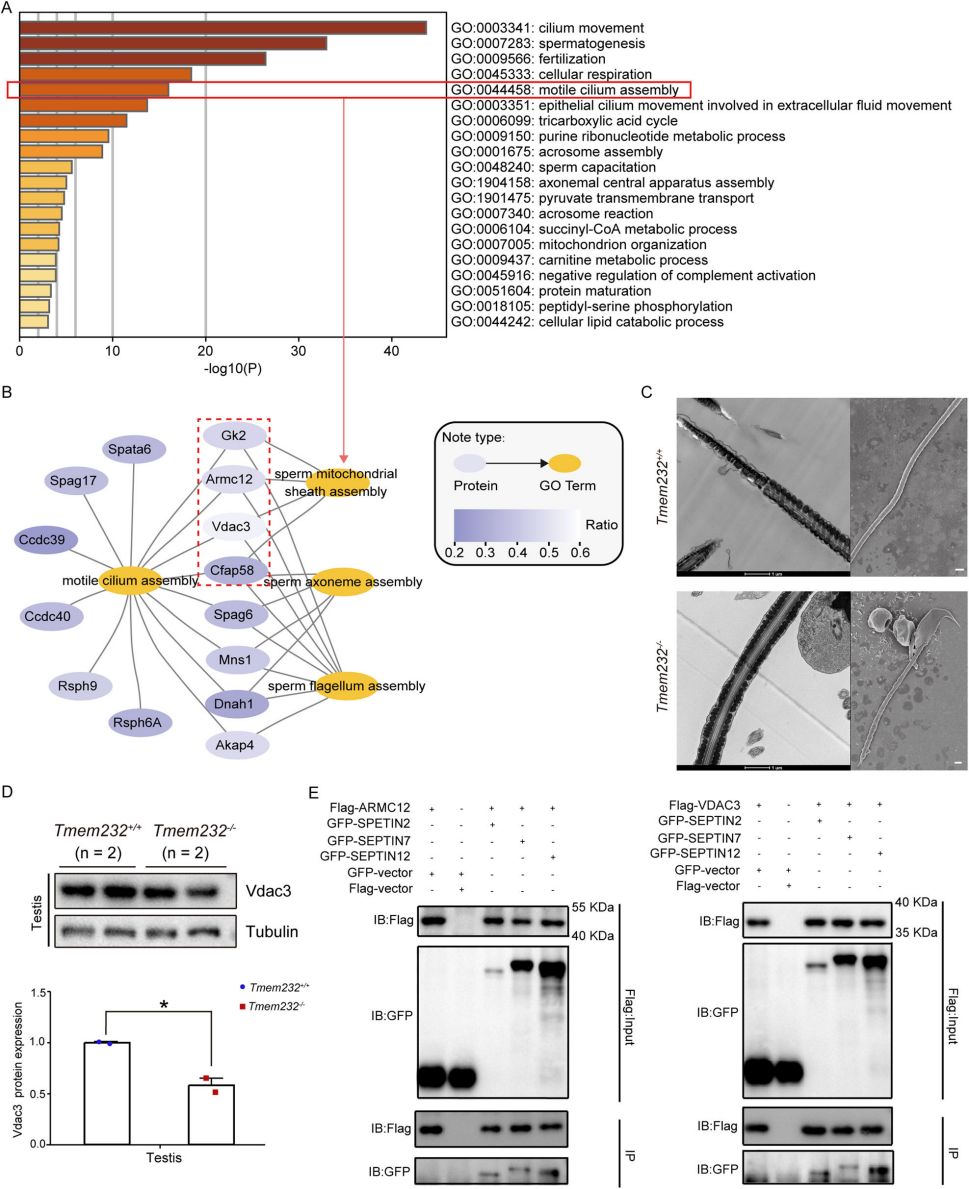
Differential expression of mitochondrial sheath structural proteins may contribute to the loss of annulus-MS junction in *Tmem232*^*−/−*^ sperm. **A** Bar graph of the pathway enrichment for downregulated proteins in *Tmem232*^*−/−*^ spermatozoa identified via proteomic analysis using the Metascape database. Only the significant GO terms (*P* < 0.05) are shown in rows. Terms with the prefix “GO” are from the Gene Ontology consortium. **B** Gene network of the sub-pathways for the significantly enriched pathway “motile cilium assembly.” **C** The MS of *Tmem232*^*+/+*^ and *Tmem232*^*−/−*^ mouse spermatozoa collected from the caudal epididymis was observed via TEM and SEM. Scale bars: 1 μm. **D** Western blotting analysis for the comparation of VDAC3 protein levels between *Tmem232*^*−/−*^ and *Tmem232*^*+/+*^ mouse testes. The α-Tubulin served as a loading control. The corresponding optical density readings for each image are shown below. The representative image of biological duplicates is shown. **E** The co-immunoprecipitation assay indicated the interaction between the ARMC12 (left) or VDAC3 (right) protein and SEPTIN 2, 7, or 12 in cells. Anti-Flag beads were used for immunoprecipitation. Anti-Flag and anti-GFP antibodies were used for western blotting analysis. The representative image of biological duplicates is shown.

Another, three sub-pathways of the significantly enriched pathway “motile cilium assembly” included “sperm MS assembly,” “sperm axoneme assembly,” and “sperm flagellum assembly” (Fig. 6A, B). This is consistent with the phenotypic variance observed in *Tmem*232^−/−^ mice, including an aberrant midpiece–principal piece junction, deletion of the fourth doublet microtubule, and abnormality of the MS (Fig. 6C). For MS, *Tmem232*^*−/−*^ mouse sperm exhibited irregular arrangement, and four proteins (*Outer Mitochondrial Membrane Protein Porin 3*, VDAC3; *Armadillo Repeat Containing 12*, ARMC12; GK2, and CFAP58) enriched in the “sperm MS assembly” pathway were considered the potentially responsible gene. Western blotting verified that VDAC3 was downregulated in the testes of *Tmem*232^−/−^ mice (Fig. 6D). Co-immunoprecipitation experiments showed that, as important components of the MS, ARMC12 and VDAC3 could interact with SEPTIN 2, 7, and 12, which may mediate the connection between the MS and the sperm annulus, and their downregulation led to the loss of connection (Fig. 6E). Our findings revealed that TMEM232 is required to maintain the normal structure and energy metabolism of sperm flagella by regulating the expression of key factors.

### TMEM232-deficient mice exhibit ribosomal protein accumulation in the testis

**Fig. 7:**
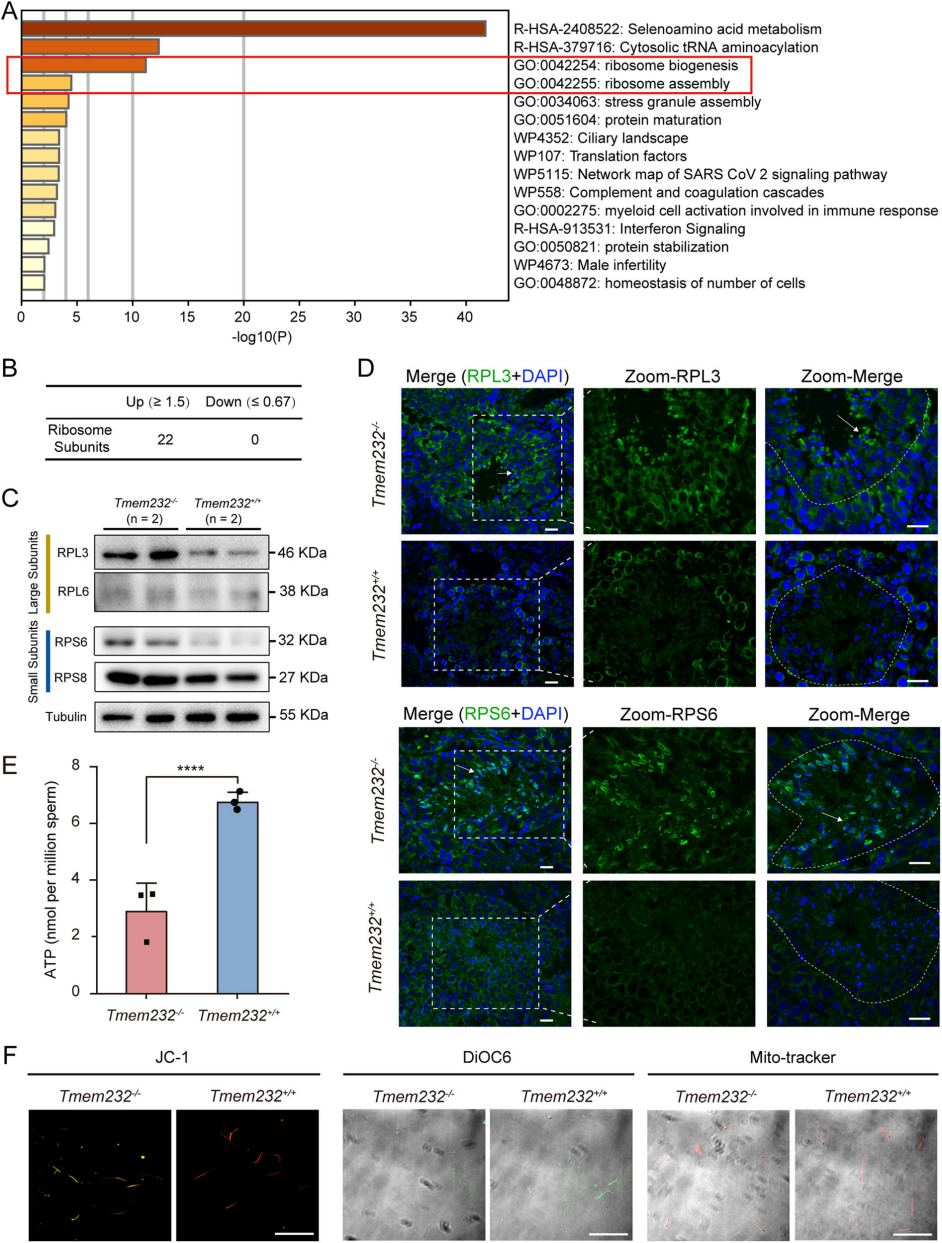
TMEM232 deficiency leads to the accumulation of ribosomal proteins in mouse testis. **A** Bar graph of the pathway enrichment for upregulated proteins in *Tmem232*^*−/−*^ spermatozoa identified via proteomic analysis using the Metascape database. Only significant GO terms (*P* < 0.05) are shown in rows. Terms with the prefix “R-HSA” are from the Reactome, the prefix “GO” from the Gene Ontology consortium, and the prefix “WP” from the WikiPathways. **B** Number of significantly upregulated or downregulated ribosomal subunit proteins identified using proteomic analysis. **C** Western blotting analysis revealed the protein levels of large and small ribosomal subunits, respectively, in the testes of *Tmem232*^*+/+*^ (*n* = 2) and *Tmem232*^*−/−*^ (*n* = 2) male mice at 2-months-old. α-Tubulin served as a loading control. The representative image of biological duplicates is shown. **D** Immunofluorescent staining (IF) of ribosomal proteins RPL3 and RPS6 on frozen sections of *Tmem232*^*+/+*^ (*n* = 3) and *Tmem232*^*−/−*^ (*n* = 3) male mice testis. Blue, DAPI; green, RPL3 or RPS6. A detailed explanation of the schematic diagram is given in the text. Scale bars, 20 μm. **E** ATP levels were significantly decreased in *Tmem232*^−/−^ mouse sperm compared with those in the controls. *Tmem232*^+/+^, 6.78 ± 0.18; Tmem232^−/−^, 2.92 ± 0.56. Data are presented as mean ± SEM. **** indicates *P* < 0.0001; *n* = 3 for each genotype. **F** Fluorescence microscopy observation of sperm obtained from *Tmem232*^*+/+*^ (*n* = 2) and *Tmem232*^*−/−*^ (*n* = 2) male mice after JC-1 staining, DiOC6, or Mito-Tracker staining. Scale bars, 100 μm.

Especially notable was, proteomics analysis revealed that multiple ribosomal components were specifically enriched in *Tmem*232^−/−^ mouse sperm (Fig. 7A, Supplementary Fig. S8B). The levels of 22 ribosomal subunits were upregulated, and none were downregulated in the sperm of *Tmem*232^−/−^ mice (Fig. 7B, Supplementary Table S3). During the maturation of spermatids from round spermatids to elongated sperm, unnecessary proteins, organelles, and bulk cytoplasm are eliminated through cytoplasmic droplet extrusion or autophagy. Therefore, testis lysates were collected and subjected to immunoblotting, and the results further confirmed the expression pattern of these ribosomal subunits (RPL3, RPL6, RPS6, and RPS8) in the testes of *Tmem232*^*−/−*^ mice (Fig. 7C). Immunofluorescence assay also revealed that RPL3 and RPS6 expression were specifically upregulated in round and elongated spermatids (indicated by arrow) in the seminiferous tubules of *Tmem232*^*−/−*^ mice (Fig. 7D). These results suggest that TMEM232 deficiency induced the enrichment of ribosomes in round spermatids and elongated sperm.

Considering that the elimination of cytosolic ribosomes during spermiogenesis provides energy for flagellar motility [7], we analyzed the energy status of sperm from TMEM232-deficient mice. The results indicated significantly decreased ATP content in *Tmem232*^*−/−*^ mouse sperm compared with that in WT mouse sperm (Fig. 7E). Additionally, compared with the active mitochondria (JC-1 color red) in WT mouse sperm, *Tmem232*^*−/−*^ mice exhibited a notably lower mitochondrial membrane potential (MMP) (JC-1 color green). Moreover, 3,3’-dihexyloxacarbocyanine iodide (DiOC6) and Mito-Tracker (red) staining further confirmed the low mitochondrial energy status in *Tmem232*^*−/−*^ mouse sperm (Fig. 7F). These results indicate that the absence of TMEM232 disrupted ribosomal quantity control during spermiogenesis and adversely affected sperm energy supply for its motility.

### TMEM232 functions in autophagy in mouse testis

**Fig. 8:**
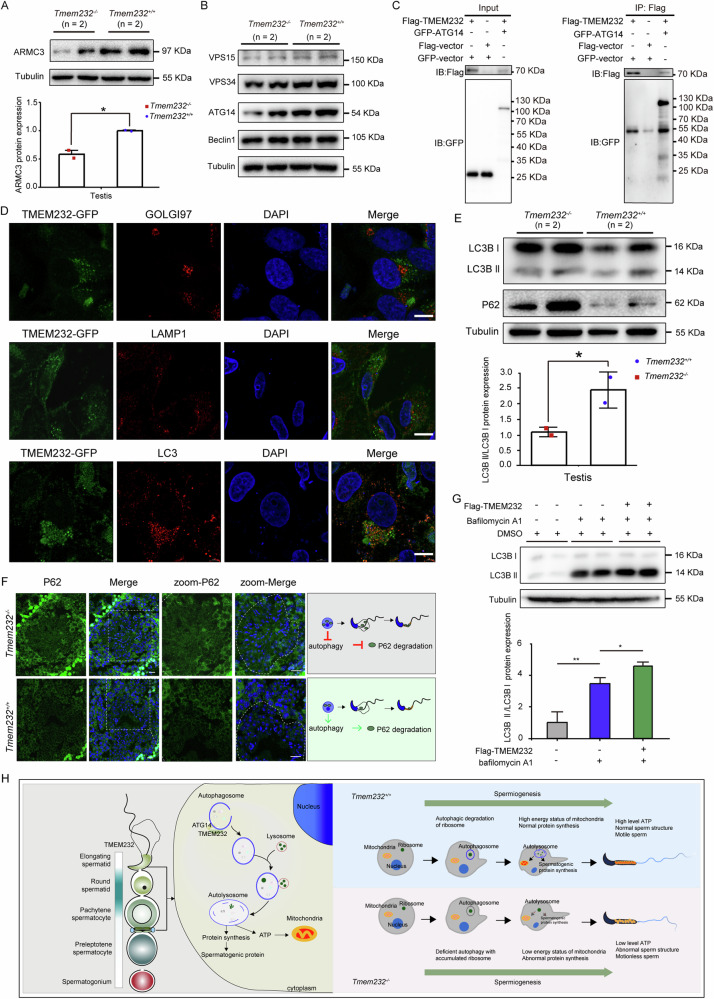
TMEM232 functions in autophagy in mouse testis. **A** Western blotting analysis for the comparison of ARMC3 protein levels between *Tmem232*^*−/−*^ (*n* = 2) and *Tmem232*^*+/+*^ (*n* = 2) mouse testes and sperms at 2-months-old. α-Tubulin served as a loading control. The corresponding optical density readings for each image are shown. The representative image of biological duplicates is shown. **B** Western blotting analysis revealed the protein levels of four subunits (VPS15, VPS34, ATG14, and Beclin1) of PIK3C3-C1 complex in the testes of 2-month-old *Tmem232*^*+/+*^ (*n* = 2) and *Tmem232*^*−/−*^(*n* = 2) male mice. α-Tubulin served as a loading control. The representative image of biological duplicates is shown. **C** Co-immunoprecipitation assay results revealed the interaction between the TMEM232 and ATG14 protein in cells. Anti-Flag beads were used for immunoprecipitation. Anti-Flag and anti-GFP antibodies were used for western blotting analysis. The results shown are representative of three independent experiments. The representative image of biological duplicates is shown. **D** TMEM232-GFP was partly colocalized with LC3 in HeLa cells. Subcellular localization of target proteins (red) was probed with an anti-GOLGI97 antibody (upper panel, marker of Golgi apparatus), an anti-LAMP1 antibody (middle panel, marker of lysosome), and an anti-LC3 antibody (lower panel, marker of autophagosome). Cell nuclei were counterstained with DAPI. Scale bars, 10 μm. **E** The absence of TMEM232 in mice resulted in the accumulation of P62 and LC3. *Tmem232*^*+/+*^ (*n* = 2) and *Tmem232*^*−/−*^ (*n* = 2) mouse testes were separated and prepared for western blotting analysis. α-Tubulin served as a loading control. The corresponding optical density readings for each image are shown. The representative image of biological duplicates is shown. **F** Immunofluorescence staining of the P62 protein performed on frozen sections of *Tmem232*^*+/+*^ (*n* = 2) and *Tmem232*^*−/−*^ (*n* = 2) mouse testes. Blue, DAPI; green, P62. Scale bars, 20 μm. **G** Western blotting analysis of LC3B protein levels in HeLa cells after Flag-TMEM232 overexpression treatment with or without bafilomycin A1 (BafA1, 50 nM) treatment. HeLa cells were pretreated with Baf-A1 for 1 h and transfected with *p-TMEM232×3FLAG-Myc-CMV-24* plasmid for 24 h. α-Tubulin served as a loading control. The corresponding optical density readings for each image are shown below. The results shown are representative of three independent experiments. The representative image of biological duplicates is shown. **H** A proposed model for the assumption that TMEM232 is involved in autophagy to regulate sperm formation in mice.

A previous study demonstrated that ribosome removal was disrupted in the elongated spermatids of *ARMC3* knockout mice, which led to ribosomal protein accumulation, immotile flagella, and complete male infertility [17]. Our proteomic analysis indicated that ARMC3 expression was significantly downregulated in the testis of *Tmem232*^*−/−*^ mice compared with those in *Tmem232*^*+/+*^ mice, a finding confirmed by immunoblotting (Fig. 8A). ARMC3 is involved in early autophagic events through interactions with the mammalian PIK3C3-C1 subunits VPS34/PIK3C3, VPS15/p150/PIK3R4, Beclin1, and ATG14 in mouse testes [17]. These proteins had relatively stable expression except ATG14 in the testis of *Tmem232*^*−/−*^ mice (Fig. 8B). Notably, TMEM232 also exhibited a binding capacity with ATG14 (Fig. 8C). Mammalian LC3 plays an essential role in autophagosome formation and is subject to autophagic degradation. SQSTM1/P62 serves as a receptor for targeting substrate cargos to autophagosomes and is degraded along with the cargos through autophagy [26]. In HeLa cells, exogenous TMEM232 exhibited the cellular localization consistent with some LC3, not with GOLGI97 and LAMP1 (Fig. 8D). The reduced ratio of active form of LC3B (LC3B-II) and the accumulation of P62 in TMEM232-deficient testes indicated a significant blockade of autophagy owing to TMEM232 deletion (Fig. 8E). Upregulated expression of P62 was also detected in the seminiferous tubules of *Tmem232*^*−/−*^ mice (Fig. 8F). Subsequently, autophagosome synthesis was monitored in cells treated with bafilomycin A1, a V-ATPase inhibitor that blocks the fusion of autophagosomes with lysosomes to inhibit their degradation. Western blotting results showed that TMEM232 overexpression increased the protein expression level of LC3B in bafilomycin A1-treated cells, suggesting that TMEM232 might involve in the regulation of autophagy (Fig. 8G). According to the transcriptome analysis of the 2-months-old testes from *Tmem232*^*−/−*^ and *Tmem232*^*+/+*^ mice, including 876 upregulated and 10 downregulated (fold change ≥2, *P* < 0.05) identified, the up-regulation or down-regulation of proteins were not specifically attributable to their transcription (Supplementary Fig. S9, Supplementary Table S2). It hints that TMEM232 may mainly affect protein expression through post-translational regulation, mirroring the possibility that TMEM232 is involved in autophagy to regulating the abundance of protein.

So, we speculated that TMEM232 might be involved in autophagy by interacting with autophagy-related proteins, such as ATG14, thereby affecting the abundance of proteins which contribute to the sperm phenotype in TMEM232 KO mouse. TMEM232-deficient male germ cells exhibited the dysregulation of autophagy flux, leading to the accumulation of ribosomal protein and consequent failure to provide energy and substrate resources for mitochondria or protein synthesis (Such as ATAT1, SEPTINs, ARMC3, et al.) during spermatogenesis. Owing to the essential role of TMEM232 during spermatogenesis, TMEM232-deficient mice exhibited abnormal sperm phenotypes, and insufficient energy supply, resulting in male infertility (Fig. 8H).

## Discussion

In this study, we explored the potential role of TMEM232 in fertility using *Tmem232* KO mice generated with the CRISPR-Cas9 technology. Our functional screenings demonstrated that *Tmem232* is essential for male fertility. Structural abnormalities in the *Tmem232*^*−/−*^ sperm flagella responsible for immobility and infertility occur at the axonemal MTDs and annulus between midpiece-principal piece junction.

The 9 + 2 microtubules of sperm flagella comprise a central pair of microtubules surrounded by nine peripheral MTDs in a specific order [27]. The T-shaped RSs structure connects the outer MTDs to the central pair of microtubules. The nine MTDs are numbered clockwise, and the MTD perpendicular to the central microtubules is defined as the first [28]. Each MTD comprises of a circular A tubule and a partial B tubule, and the lumen surface of MTD is highly decorated with microtubule-associated proteins, which are important for MTD stability [29–36]. For example, *Dnah17*, *Vdac3*, and *Pla2g3* loss induces destabilization of sperm MTDs 4–7, causing asthenozoospermia, whereas *Ccdc176* KO mouse sperm exhibit axonemal structure deficiency in the absence of *Dmt1* and/or 9 (missing *Dmt1*, *Dmt9*, or both). The deletion of *Tssk4* results in the loss of doublet microtubules 1–4 in the sperm flagellum and an abnormal sperm annulus, a phenotypic defect similar to that of *Tmem232*^*−/−*^ mouse sperm. Here, we found that lack of TMEM232 would result in absence of the fourth outer microtubule doublet. Previous study has reported that the sperm flagellum of CFAP97 domain-containing 1 (Cfap97d1)-KO mice also lacked the fourth doublet microtubule [32]. However, the underlying molecular mechanisms remain unknown till now [37].

As the motile cilium and sperm flagellum share the same 9 + 2 microtubular arrangement, we speculate that the genetic defect of *Tmem232* in sperm flagella may also affect motile cilia. However, T*mem232*-null mice exhibited no respiratory symptoms, such as coughing or sneezing. TEM analysis of motile cilia on the surface of tracheal epithelial cells demonstrated a normal 9 + 2 structure, coordinated directionality of the axonemes, and complete basal feet. SEM analyzes revealed that the cilia were shorter and fewer in *Tmem232-*null tracheal epithelium cells than in wild-type mice. The profile of microtubule proteins in mammalian sperm, which was recently identified by cryo-electron microscopy, confirmed the unidentical compositions of flagella and cilia microtubule protein [29, 37–41]. These findings indicated that the functional features of TMEM232 are tissue specific.

Furthermore, we identified that TMEM232 is a testes-enriched gene in both humans and mice. Tmem232 is expressed at P21 in mouse testes, when round post-meiotic spermatids are highly represented and flagella begins to form. Tmem232 plays a role in the formation of the axoneme and accessory structure in elongating spermatids through the interaction with ATAT1, SEPTINs, and ARMC3 along with other proteins. The degree of α-Tubulin acetylation, which has been implicated in the regulation of microtubule stability and function [42], was lower in the sperm of *Tmem232*^*−/−*^ mice compared to that in wild-type mice. Interestingly, TMEM232 can interact with the ATAT1 protein, which enters the lumen of the microtubule and promotes α-Tubulin acetylation [23, 24]. In neurons, ATAT1 is transported to the cytosolic side of neuronal vesicles moving along the axons, and the ATAT1-enriched vesicles are regarded as the predominant drivers of α-Tubulin acetylation in the axons [43]. In cultured cells, TMEM232 is localized in the plasma membrane and some vesicles. Therefore, TMEM232 may affect the distribution or local activity of intracellular ATAT1 to reduce the α-Tubulin acetylation level of -Tubulin, resulting in the instability of doublet microtubules of sperm. These results suggest that TMEM232, as a potential scaffold protein, involves in the correct assembly and distribution of certain functional complexes by recruiting key intracellular proteins, such as ATAT1, onto trafficking vesicles and is essential for the formation of a highly structured flagellum.

One of the most fascinating features of *Tmem232*^*−/−*^ mouse spermatozoa was the abnormal midpiece-principal piece junction. The sperm annulus is a ring structure connecting the midpiece and the principal piece of the sperm flagellum, which comprises a complex of SEPTIN 1, 2, 4, 6, 7, and 12 associated with the co-chaperone DNAJB13 [44–48]. Protein mass spectrometry data showed that DNAJB13 were downregulated in *Tmem232*^*−/−*^ mouse sperm. Subsequently, western blotting analysis and immunofluorescence staining demonstrated that SEPTIN 2, 6, 7, and 12 were also downregulated in *Tmem232*^*−/−*^ mouse sperm, which may be responsible for the loss of annulus-MS (mitochondrial sheath) junction. Moreover, the MS exhibited an irregular arrangement in *Tmem232*^*−/−*^ mouse sperm, which might affect the function of mitochondria [49, 50]. GO analysis of the downregulated proteins in *Tmem232*^*−/−*^ mouse sperm revealed that the DEPs, including ARMC12, GK2, VDAC3, and CFAP58, were enriched in flagellar assembly-related pathways, including the sperm MS assembly pathway. ARMC12 is a mitochondrial peripheral membrane protein that has been implicated in the abnormal arrangement of MS, causing infertility in mice [51]. Co-immunoprecipitation assays showed that ARMC12 could interact with SEPTIN 12, which was disappeared in *Tmem232*^*−/−*^ mouse sperm. Based on the flagellar structure of sperm, we speculated that the downregulation of ARMC12 and some SEPTINs might weaken the connection between the annulus ring and MS, aggravating the abnormalities at the midpiece-principal piece junction.

Autophagy is a catabolic recycling pathway responsible for degrading and recycling cytoplasmic components, such as ribosomes, to modulate various physiological and pathological events, including spermatogenesis [16, 17]. This study also revealed that TMEM232 was likely involved in autophagy to regulate the spermatogenesis for the following reasons: 1) TMEM232 interacted with the ATG14 protein, a member of the PIK3C3-C1 complex responsible for regulating autophagosome formation; 2) TMEM232 co-localized with LC3 in cultured cells, and TMEM232-deficient testes exhibited the accumulation of LC3 and P62, along with a decreased ratio of the active form of LC3; 3) TMEM232 exogenous overexpression induced an increase in the expression of LC3. The removal of cytoplasmic organelles during spermiogenesis by autophagy is essential for mature sperm formation [13, 15, 52]. ARMC3, an essential protein for sperm motility and fertility, has been identified as an autophagy regulator [17]. In the present study, *Tmem232*-knockout mice exhibited downregulated expression of ARMC3 and an accumulation of ribosomal proteins, which further support the possibility that TMEM232 is involved in the autophagic removal of excessive cytoplasmic materials, such as ribosomes, to provide energy resources for spermatids during the transition from round into elongated spermatids. Autophagy facilitates the removal of specific organelles, ribosomes, and protein aggregates through either non-selective or selective pathways [26, 53]. So, we speculated that the failure of ribosomal degradation resulting from TMEM232 deficiency would disrupt the steady-state balance of intracellular proteins. Another, TMEM232 could interact with septins and partly colocalized in some vesicles. Recently, Septins was discovered to contribute to autophagosome biogenesis, and several vesicular trafficking processes participate in autophagy flux [54–56]. So, we suggested that TMEM232, as a scaffold protein, could cooperate with septins to regulate the autophagosome formation. However, further experimental evidence is required to confirm this hypothesis.

Our study provided the phenotypic identification and molecular dissection of mouse infertility caused by *Tmem232* deficiency. Here, we show that the lack of *Tmem232* leads to the deletion of the fourth doublet microtubule, a midpiece-principal piece junction defect, and irregular MS arrangement in sperm, which finally cause infertility. TMEM232 could interact with ATAT1 to modulate the acetylation of α-Tubulin, which is responsible for the stability of microtubules. *Tmem232* KO would disrupt the interaction between SEPTINs with ARMC12 and VDAC3, which lead to the loss of connection between the annulus ring and MS. Furthermore, we also provided evidence that TMEM232 may be involved in selective autophagy by interacting with ATG14 during spermiogenesis to subsequently provide substrates for sperm protein synthesis and energy for flagellar movement. Our findings provide a better understanding of the function of TMEM232 in sperm formation, advancing the knowledge of male infertility. However, the lack of a specific TMEM232 antibody for IF prevented the determination of its endogenous location and functional complex in mouse testis. The molecular mechanism underlying the deletion of the fourth doublet microtubule in *Tmem232*^*−/−*^ mice also remains unclear. In the future, structural and dynamic analysis of TMEM232-associated protein complex may help to clarify its function during spermiogenesis.

## Materials and methods

### Experimental animals and ethics approval statement

The mice were housed and reared in specific pathogen-free conditions under a 12-h light/dark cycle with controlled temperature (25 ± 2 °C) and humidity (45-60%). Mice were provided autoclaved food and water. Guidelines from the Laboratory Animal Ethics Commission of Chinese Academy of Sciences were used for the animal procedures in this study, and all animal experiments were approved by the Animal Experimentation Ethics Committee of Anhui Medical University (Animal Ethics Committee approval no. 20190403). The experiments involving animals were performed in accordance with “Instructive Notions with Respect to Caring for Laboratory Animals” established by the Ministry of Science and Technology of the People’s Republic of China.

### Cell culture and transfection

GC-1, MGC803, HeLa, and HEK-293T cell lines were obtained from the Shanghai Institute of Cell and Tongpai Biotechnology Co., Ltd. (Shanghai). Regular authentication of cell lines was conducted through short tandem repeat profiling and mycoplasma testing. All cells were cultured in Dulbecco’s Modified Eagle’s Medium (DMEM) with 4.5 g/L D-glucose (Cat^#^ A4192101; Gibco, Thermo Fisher Scientific, Waltham, MA, USA) supplemented with 10% fetal bovine serum (Cat^#^C04001; VivaCell, Shanghai, China) and 1% penicillin-streptomycin (Cat^#^15140122; Gibco, USA). Transfection of plasmids into cells was conducted using Lipofectamine 2000 (Cat^#^ 11668019; Thermo Fisher Scientific, USA) when cell density reached approximately 85%. This experiment was performed in biological duplicates.

### Purification of GST-TMEM232 fusion protein and antibody testing

Recombinant GST-TMEM232 fusion proteins were prepared and expressed in *Escherichia coli* BL21 (DE3) cells. After being induced with isopropyl-β-D-thiogalactopyranoside, bacterial cultures were harvested and disrupted by sonication. The supernatants of cell lysates were mixed with 50% glutathione–Sepharose beads and incubated for 1 h at 4 °C with gentle rocking motion on a rotating platform. After washing three times with wash buffer, the proteins bound to the resin were eluted with elution buffer, mixed with sodium dodecyl sulfate (SDS) loading buffer, and boiled for 10 min. Finally, the samples were separated using SDS-polyacrylamide gel electrophoresis (SDS-PAGE) followed by western blotting with anti-TMEM232 antibody to evaluate its specificity and binding ability.

### Isolation of spermatogenic cells

Spermatogonia, pachytene spermatocytes, round spermatids, and elongated spermatids were isolated using the STA-PUT method. Sertoli cells and spermatogonia were isolated from mice on postnatal day 6-8, whereas pachytene spermatocytes and round spermatids were isolated from adult mice. Briefly, mouse testes were digested with collagenase IV (Cat^#^ C4-28-100MG, Merck & Co., Inc., Rahway, NJ, USA,). The Dulbecco’s Modified Eagle’s Medium (DMEM, Gibco, Grand Island, NY, USA) was used to wash dispersed seminiferous tubules and then centrifuged at 500 × *g*. Further digest the pellet with DNase I (Cat^#^ D7073; Beyotime, Shanghai, China) containing 0.25% trypsin and filter with 40 µm mesh screens to obtain a single-cell suspension. The separation apparatus (ProScience, Don Mills, Ontario, Canada) was used to isolate cells from single-cell suspension with 2–4% BSA gradient (2% BSA and 4% BSA in DMEM) loaded into the separation apparatus chamber. Cell fractions were harvested after 1.5–3 h of sedimentation. According to cell diameter, morphological characteristics and DAPI staining under a light microscope, different cell types were identified. The purity of round spermatids, pachytene spermatocytes and spermatogonia was approximately 90%, and then divided each type of cell into two parts, one for RT-qPCR experiments and the other for immunoblotting analysis.

### RNA extraction and quantitative real-time polymerase chain reaction (RT-qPCR)

Spermatogenic cells were isolated from postnatal day 6 and two-month-old wild-type mice. Tissues, including heart, spleen, liver, lung, kidney, ovary, trachea, skin, testis, and epididymis, were collected from two-month-old wild-type mice. Additionally, integral fresh mouse testes were obtained from P7, P14, P21, P28, P35, and P56 wild-type mice. Finally, integral fresh mouse testes were collected from wild-type and *Tmem232* KO mice at two months old. These samples were subjected to RNA extraction using TRIzol reagent (Thermo Fisher Scientific, Waltham, MA, USA) according to the manufacturer’s instructions. Various spermatogenic cells isolated from fresh mouse testis tissues at two months old using the STA-PUT method were rapidly resuspended in TRIzol after washing twice with phosphate-buffered saline (PBS). For fresh tissue samples, TRIzol was added after grinding on liquid nitrogen. RNA quantity and integrity were assessed using a NanoDrop One Microvolume UV-Vis Spectrophotometer (Thermo Fisher Scientific, Waltham, MA, USA) and by agarose gel electrophoresis, respectively. First-strand cDNA was synthesized using the RevertAid First-Strand cDNA Synthesis Kit (Thermo Fisher Scientific, Waltham, MA, USA) according to the manufacturer’s instructions. qPCR was performed on a Bio-RadCFX96 Real-Time System (Bio-Rad, Hercules, CA, USA) using qPCR SYBR SuperMix Plus (Novoprotein, Suzhou, China). All reactions were prepared in triplicate, and the relative amounts of mRNAs were calculated using the comparative 2^-ΔΔCT^ method with *Gapdh* as the endogenous control for normalization. The primers used for qPCR are listed in Supplementary Table S5. All experiments were performed in biological duplicates.

### Western blotting

Proteins from cultured cells and mouse tissues were extracted using radioimmunoprecipitation assay buffer (Cat^#^ 89901; Thermo Fisher Scientific, Waltham, MA, USA) containing a 1× phenylmethylsulfonyl fluoride protease inhibitor mixture (Cat^#^ 36978; Thermo Fisher Scientific, Waltham, MA, USA). The lysates were clarified by centrifugation (12,500 × *g* at 4 °C for 20 min) to remove insoluble material. The supernatant was collected, and the protein concentration was measured through a BCA assay following the manufacturer’s instructions. The protein fractions were heated at 100 °C for 10 min, separated using SDS-PAGE on 10% gel, and transferred to polyvinylidene difluoridemembranes (Cat^#^ 162–0177; Bio-Rad, Richmond, CA, USA). The membranes were blocked with 5% non-fat milk for 1 h at room temperature and incubated with primary antibodies overnight at 4 °C. After washing three times with phosphate buffered saline (PBS) containing 0.1% Tween-20 (PBST), the membranes were incubated with secondary antibodies at room temperature for 40 min. The signals from the detected proteins were visualized using SuperSignal West Femto Chemiluminescent Substrate (Cat^#^ 34094; Thermo Fisher Scientific, Waltham, MA, USA). The following primary antibodies were used: rabbit anti-Lamin B1 (Cat^#^ 12987-1-AP; Proteintech, Chicago, IL, USA, 1:5000), mouse anti-α-Tubulin (Cat^#^ 66031-1-lg; Proteintech, Chicago, IL, USA, 1:10000), rabbit anti-SEPTIN2 (Cat^#^ 11397-1-AP; Proteintech, USA, 1:2000), rabbit anti-SEPTIN4 (Cat^#^ 12476-1-AP; Proteintech, Chicago, IL, USA, 1:1000), rabbit anti-SEPTIN6 (Cat^#^ 12805-1-AP; Proteintech, Chicago, IL, USA, 1:1000), rabbit anti-SEPTIN7 (Cat^#^ 13818-1-AP; Proteintech, Chicago, IL, USA, 1:3000), rabbit anti-SEPTIN12 (Cat^#^ PA5-98593; Invitrogen, Waltham, MA, USA, 1:1000), rabbit anti-SEPTIN14 (Cat^#^ 24590-1-AP; Proteintech, Chicago, IL, USA, 1:2000), rabbit anti-TSSK4 (Cat^#^ A7861; ABclonal Biotechnology, Wuhan, China, 1:2000), rabbit anti-Ac-α-Tubulin (Cat^#^ 5335; CST, Danvers, Massachusetts, USA, 1:1000), rabbit anti-CatSper2 (Cat^#^orb354243; Biorbyt, Cambridge, UK, 1:2000), rabbit anti-ODF1 (Cat^#^ 24736-1-AP; Proteintech, Chicago, IL, USA, 1:1000), rabbit anti-ODF2 (Cat^#^ 12058-1-AP; Proteintech, Chicago, IL, USA, 1:2000), rabbit anti-PDHA2 (Cat^#^ 17134-1-AP; Proteintech, Chicago, IL, USA, 1:2000), rabbit anti-COX6B2 (Cat^#^ 11437-1-AP; Proteintech, Chicago, IL, USA, 1:2000), rabbit anti-VDAC3 (Cat^#^ 14451-1-AP; Proteintech, Chicago, IL, USA, 1:2000), rabbit anti-GFP (Cat^#^ 50430-2-AP; Proteintech, Chicago, IL, USA, 1:3000), mouse anti-FLAG (Cat^#^ 66008-3-Ig, Proteintech, Chicago, IL, USA, 1:2000), anti-GST tag (91G1) (Cat^#^ 2625; CST, Danvers, Massachusetts, USA, 1:1000), rabbit anti-TMEM232 (generated by ABclonal Biotechnology, Wuhan, China, 1:1000), anti-ATAT1 (Cat^#^28828-1-1-AP; ProteinTech Group, Chicago, IL, USA), anti-RPL3 (Cat^#^11005-1-AP; ProteinTech Group, Chicago, IL, USA), anti-RPL6 (Cat^#^A15094; ABclonal Biotechnology, Wuhan, China, 1:200), anti-RPS6 (Cat^#^A11874; ABclonal Biotechnology, Wuhan, China, 1:200), anti-RPS8 (Cat^#^A21124; ABclonal Biotechnology, Wuhan, China, 1:200), anti-LC3 (Cat^#^BM4827; Boster, Wuhan, China), anti-ARMC3 (Cat^#^28418-1-AP), ATG14 (Cat^#^19491-1-AP), anti-VPS15 (Cat^#^17894-1-AP), anti-VPS34 (Cat^#^12452-1-AP), anti-Beclin1 (Cat^#^17894-1-AP), and anti-P62 (Cat^#^29503-1-AP) (ProteinTech Group, Chicago, IL, USA). Secondary antibodies included horseradish peroxidase (HRP)-conjugated donkey anti-rabbit IgG (Cat^#^ 406401; Biolegend, Shanghai, China, 1:10,000) and HRP-conjugated goat anti-mouse IgG (Cat^#^ 405306; Biolegend, Shanghai, China, 1:10,000). All experiments were performed in biological duplicates.

### Immunofluorescence (IF)

Sperm samples were obtained from the caudal epididymis, washed twice with PBS and spread onto glass slides. Slides were fixed with 4% paraformaldehyde, permeabilized with 0.1% Triton X-100 for 10 min, blocked with 1% bovine serum albumin (BSA), and then incubated with Rhodamine-phalloidin (Cat^#^ R415; Invitrogen, Waltham, MA, USA, 1: 400), mouse anti-DMIA (Cat^#^ 3873; CST, Danvers, Massachusetts, USA, 1:1000), rabbit anti-SEPTIN7 (Cat^#^13818-1-AP, Proteintech, Chicago, IL, USA, 1:200), and rabbit anti-SEPTIN12 (Cat^#^ PA5-98593; Invitrogen, Waltham, MA, USA, 1:200), overnight at 4 °C. The samples were washed thrice with PBST before incubation with a secondary antibody (Cat^#^ 1927937; Thermo Fisher Scientific, Waltham, MA, USA, 1:400) at room temperature for 1 h. The nuclei were counterstained with 4’,6-diamidino-2-phenylindole (DAPI, Cat^#^ F6057; Sigma, Waltham, MA, USA). The acrosomes were identified using PNA (Cat^#^ RL-1072; Vector Laboratories, San Francisco, USA). All images were examined with a scanning laser confocal microscope (LSM700, Carl Zeiss, Jena, Germany).

Testis tissues from two-month-old *Tmem232*^*−/−*^ and *Tmem232*^*+/+*^ mice were fixed in 4% paraformaldehyde overnight at 4 °C. Subsequently, the tissues were dehydrated in a 25% sucrose-PBS solution for 48 h and embedded in optimal cutting temperature compound (Cat^#^ 4583; Sakura Finetek Inc., Torrance, CA, USA). Next, the testis tissues were sectioned into 8 µm slices using a freezing microtome (Leica Biosystems, Wetzlar, Germany). Frozen sections of the testis samples or cell slides were fixed in 4% paraformaldehyde, permeabilized with 0.2% Triton X-100, and blocked with 1% BSA buffer for 30 min at 20–30 °C. Subsequently, incubation was conducted using Rabbit anti HA-Tag pAb (Cat^#^AE036, ABclonal Biotechnology, Wuhan, China, 1:100), rabbit anti-RPL3 (Cat^#^11005-1-AP; ABclonal Biotechnology, Wuhan, China, 1:200), rabbit anti-RPS6 (Cat^#^A11874, ABclonal Biotechnology, Wuhan, China, 1:200), Golgin-97 (D8P2K) Rabbit mAb (Cat^#^13192S; CST, Danvers, Massachusetts, USA, 1:400), mouse anti-LAMP1 (Cat^#^55273-1-AP or 67300-1-Ig; ProteinTech Group, Chicago, IL, USA, 1:200), Anti-LC3B/MAP1LC3B Antibody (Cat^#^BM4827; Boster, Boster, Wuhan, China,1:150), and rabbit anti-P62 (Cat^#^18420-1-AP; ProteinTech Group, Chicago, IL, USA, 1:1000). The samples were washed thrice with PBST before incubation with a Alexa Fluor® 488 donkey anti-rabbit IgG (H + L) cross-adsorbed secondary antibody (Cat^#^A21206; Thermo Fisher Scientific, Waltham, MA, USA, 1:400), Donkey anti-Mouse IgG (H + L) Highly Cross-Adsorbed Secondary Antibody, Alexa Fluor™488 (Cat^#^A21202; Thermo Fisher Scientific, Waltham, MA, USA, 1:400), Alexa Fluor™555 Goat anti-Mouse IgG (H + L) cross-adsorbed secondary antibody (Cat^#^A-21422; Thermo Fisher Scientific, Waltham, MA, USA, 1:400) or Donkey anti-Rabbit IgG (H + L) Highly Cross-Adsorbed Secondary Antibody, Alexa Fluor™555 (Cat^#^A-31572; Thermo Fisher Scientific, Waltham, MA, USA, 1:400) at room temperature for 1 h. Subsequently, the nuclei were counterstained with DAPI. Images were examined using a Nikon Fully-Automated A1 Confocal Laser Microscope with a Nikon A1 LFOV camera (Nikon, Tokyo, Japan). All experiments were performed in biological duplicates.

### Construction of recombinant plasmids

Total RNA was extracted from the testes of 21-day-old mice or from HeLa cells using the TRIzol reagent (Cat^#^ 15596026; Invitrogen, Waltham, MA, USA). A PrimerScript RT reagent kit (Cat^#^ RR037B; Takara Bio Co., Ltd., Kusatsu City, Shiga Prefecture, Japan) was used to perform reverse transcription to obtain complementary DNA as a template for gene amplification. The primers used for PCR amplification and the vectors are listed in Supplementary Table S6. The recombinant plasmids were constructed using a ClonExpress II One-Step Cloning Kit (Cat^#^ C112; Vazyme, Nanjing, China).

### Generation and genotyping of *Tmem232* KO mice

*Tmem232* KO (*Tmem232*^−/−^) mice were purchased from the Nanjing Biomedical Research Institute of Nanjing University, China. *Tmem232* KO mice were generated using CRISPR-Cas9 technology. The mouse *Tmem232* gene (transcript *Tmem232-201*, ENSMUST00000062161.6) has 16 exons, with the ATG start codon in exon 4 and a TGA stop codon in exon 16. Cas9 mRNA and single guide RNAs (sgRNAs) targeting introns 4-5 and 8-9 was microinjected into the fertilized oocytes of C57BL/6 mice. A 9297-bp DNA fragment of *Tmem232* gene (chr17:65477831-65487127, genome reference mm10) was deleted, which resulted in a frameshift mutation in its open reading frame. Subsequently, the injected embryos were transferred to pseudo-pregnant host females and performed a full-term pregnancy. The genotypes of edited founders were identified by DNA sequence analysis. Genomic DNA was extracted from tail clippings and amplified by multiplex PCR using the Quick Genotyping Assay Kit for Mouse Tail (Cat^#^ D7283S; Beyotime Biotechnology, Shanghai, China). Genotyping primers for the wild-type allele were: FP: 5’-CTCCGTGTGGTGAGATTGTTATG-3ʹ and RP: 5ʹ-ATGAGAAGCTATCGAAGTGC-3’ with a 553-bp PCR product. Genotyping primers for the *Tmem232* KO allele were: FP: 5’-CGCTTCCATCTCCATTTGTCCGAC-3’ and RP: 5’- TGGGATCTGTCTCCTGCAAGTC-3’. The length of the PCR product was 610 bp.

### Fertility test

For fertility testing, randomly selected healthy 8~10-week-old wild-type (*n* = 6) or *Tmem232*-null male mice (*n* = 6) were housed with two sexually mature wild-type C57BL/6J females for 3 months. The vaginal plugs were checked every morning. The dates of birth and the number of pups of each female were recorded, with the average litter size considered as a measure of fertility. In this study, experimental animals are strictly grouped according to genotype, and a method of randomization was only used within a specific genotype mouse group to select the individuals for specific experiment.

### Sperm counting and motility assay

Sperm was obtained from the caudal epididymis of 8~10-week-old mice. The caudal parts were cut at various points, placed in 1 ml of PBS, and incubated for 30 min at 37 °C under 5% CO_2_. The sperm were transferred to a hemocytometer for cell counting after swimming out of the caudal epididymis. Sperm motility, including total motility, average path velocity, straight-line velocity, and linear velocity, was determined using a computer-assisted sperm analyzer (CASA; Hamilton Thorne, Beverly, USA). All experiments were performed in biological duplicates.

### Histological analysis

Immediately after euthanasia, at least three 2-month-old mice of each genotype were dissected to obtain samples of testes, caudal epididymides, and ovaries. All tissues were fixed in 10% (mass/vol) formalin for 24 h, dehydrated with increasing concentrations of ethanol, and embedded in paraffin. The tissue blocks were sectioned into 5-µm sections and then placed on glass slides. After deparaffinization, the slides were stained with H&E and periodic PAS for histological examination. The images were captured using a digital slide scanner (Pannoramic MIDI, 3D HISTECH, Hungary). All experiments were performed in biological duplicates.

### Electron microscopy

Scanning electron microscopy (SEM) and transmission electron microscopy (TEM) analyses were performed at Anhui Agricultural University, China and Anhui Medical University, China, respectively. For SEM, mouse sperm and trachea sections were fixed in 2.5% glutaraldehyde at 4 °C for 4 h. After being thoroughly washed in 0.1 mol/L phosphate buffer three times, the specimens were sequentially dehydrated in ascending grades of alcohol (30, 50, 70, 80, 90, and 100% for 20 min at 4 °C) and treated with 100% acetone for 20 min twice at 4 °C. The samples were fixed onto slides of 1 cm in diameter and dried using a CO_2_ critical-point dryer (Emitech K850, UK). Finally, the slides were sputter-coated with an ionic sprayer meter (Hitachi E1010, Japan) after mounted on aluminum stubs, and then analyzed by a scanning electron microscope (Hitachi S-4800, Japan). For TEM, mouse sperm, testis, and trachea sections were fixed with 2.5% glutaraldehyde overnight at 4 °C. The samples were then rinsed in 0.1 mol/L phosphate buffer three times, post-fixed in 1% osmium for 1 h at 4 °C, washed again, gradually dehydrated in ethanol (30, 50, 70, 80, 90, and 95% for 15 min each, and 100% for 20 min at 4 °C), and treated with 100% acetone for 20 min. After embedding in Epon 812, the samples were trimmed and sectioned using a diamond knife on an ultramicrotome (EM UC7, Leica, Germany), double-stained with uranyl acetate and lead citrate, and examined under a transmission electron microscope (Talos L120C G2, USA) at an accelerating voltage. This experiment was performed in biological duplicates.

### Co-immunoprecipitation assays

HEK-293T cells were cultured in DMEM (Gibco, Grand Island, NY, USA) with 10% fetal bovine serum (HyClone, Logan, UT, USA), 100 μg/ml streptomycin (Invitrogen) and 100 units/ml penicillin at 37 °C with 5% CO2. Cells were transfected with plasmids using a calcium phosphate transfection kit. After 24 h, cells were collected and lysed with lysis buffer (50 mM HEPES with pH 7.4, 150 mM NaCl, 2 mM MgCl2, 1 mM EGTA, 0.1% Triton X-100) supplemented with a protease inhibitor cocktail (Sigma) and then clarified by centrifugation at 16,000 × *g* for 15 min. The cleared cell lysate was then incubated with FLAG beads (Cat^#^ F2426, Millipore, Sigma, Waltham, MA, USA) overnight at 4 °C. After being washed three times with lysis buffer, bound proteins were resuspended in 1× SDS sample buffer and denatured at 95 °C for 10 min. Proteins were fractionated on SDS–polyacrylamide gels and transferred onto polyvinylidene difluoride membranes for western blotting analysis. The rabbit anti-FLAG antibody (Cat^#^ 20543-1-AP, Proteintech, Chicago, IL, USA, 1:4000) and rabbit anti-GFP antibody (Cat^#^ 50430-2-AP, Proteintech, Chicago, IL, USA, 1:4000) were used as primary antibodies. All experiments were performed in biological duplicates.

### Measurement of sperm ATP content

Minced caudal epididymes (separated from three two-month-old mice of each genotype) were incubated with M16 medium at 37 °C under 5% CO2 for 5 min. Sperm was immediately washed twice, resuspended at a concentration of 10^7^ cells/ml in lysis buffer, and placed on ice. Luminometric methods were used to measure ATP with an ATP assay kit (Cat^#^ S0027, Beyotime Biotechnology, Shanghai, China), according to the manufacturer’s instructions. All measurements were performed in duplicate for four animals in each genotype. The ATP content was calculated using a standard curve of ATP (range 1–1000 nM).

### RNA sequencing

The four *Tmem232*^*−/−*^ and four *Tmem232*^*+/+*^ mice testes at 2 months old were obtained to isolate total RNA using TRIzol (Cat^#^ 15596018, Thermo Fisher, Waltham, MA, USA) according to the manufacturer’s instructions. After assessing the quality of RNA by concentration determination and agarose gel electrophoresis without degradation, 1 μg of total RNA was then used to construct cDNA libraries using the TruSeq RNA Sample Preparation kit (Illumina, Inc, San Diego, California, USA). Paired-end sequencing was performed at BGIseq-50 platform (BGI, Shenzhen). An average of approximately 45 million clean reads were generated after removing low-quality reads, and then mapped to the mouse reference genome (GCF_000001635.26_GRCm38.p6; http://asia.ensembl.org/Mus_musculus/Info/Annotation/) using HISAT and Bowtie2 [57]. The StringTie [58] and featureCounts [59] were used to calculate the counts of all annotated genes. Then, differentially expressed genes (DEGs) were identified by the “DESeq2” R package.

### Proteomic analysis

Sperm was harvested from the caudal epididymes of 8-week-old *Tmem232*^*−/−*^ (*n* = 3) and wild-type adult male mice (*n* = 3) and then subjected to Liquid chromatography tandem-mass spectrometry (LC-MS/MS) analysis. The mouse sperm samples were dissolved in lysis buffer (8 M urea and 1% protease inhibitor cocktail) and sonicated on ice. After centrifugation, the supernatant was collected, and the protein concentration was determined using a BCA kit following the manufacturer’s instructions. The peptides were reconstituted in 0.5 M TEAB after trypsin digestion and purification and then processed using the TMT kit according to the manufacturer’s protocol. LC-MS/MS identification and quantification of released peptides were performed on tims-TOF Pro (Bruker Daltonics, Germany) coupled online to the ultra-performance liquid chromatography (UPLC).

The resulting sequences were searched against the UniProt mouse proteome database using the MaxQuant software (version 1.6.17.0) (mass error tolerances of 20 ppm and 0.02 Da) after processing raw MS/MS data. Carbamidomethylation on Cys was designated as a fixed modification, whereas acetylation and oxidation on Met were designated as variable modifications. False discovery rate estimation was performed by adjusting it to <1% at the peptide-spectrum match level for all experiments. The minimum score for the modified peptides was set at 40. The proteins that shared significant peptide evidence were grouped together. The average expression values of the replicates were used to determine the absolute protein abundance in a given sample. The T-test was used to compare differentially abundant proteins between the two samples. The ratio threshold for upregulation was set to 1.50 and reciprocally 0.67 for downregulation, with a *P-*value of <0.05. DEPs were further analyzed using bioinformatics methods. Principal component analyses were performed for all expressed proteins identified in each sample (*Tmem232*^*−/−*^ and wild-type) using the R package “pcaMethods.” Scatter plots showing the distribution of the samples in the two main components (PC1 and PC2) were generated. Protein pathways were annotated using the Kyoto Encyclopedia of Genes and Genomes (KEGG) database. A protein–protein interaction (PPI) network was derived from the STRING database version 11.5 (https://string-db.org) [60]. All DEPs were searched against the STRING database for PPIs, and interactions with a confidence score of >0.7 were obtained. The Cytoscape software (Version 3.8.2) was then used to visualize and analyze the interaction network to identify the key proteins [61]. ClueGo [62] was used for Gene Ontology (GO) enrichment. The clustering relationships of the DEPs were visualized using R software v.4.1.1.

### Detection of the mitochondrial membrane potential of sperm

Sperm samples were separated and numbered from three two-month-old mice of each genotype to measure sperm MMP. After washing twice with PBS, the sperm samples were stained with JC-1 (Cat^#^T3168, Thermo Fisher Scientific, Waltham, MA, USA, working concentration 1 μM), DiOC6 (Cat^#^D273, Thermo Fisher Scientific, Waltham, MA, USA, working concentration 175 nM), and mito-tracker (Cat^#^M7512, Thermo Fisher Scientific, Waltham, MA, USA, working concentration, 200 nM) for 30 min at 37 °C in the dark. The sperm samples were immediately washed twice, resuspended in 4% paraformaldehyde buffer, and fixed for 5 min. After washing with PBS and subsequent centrifugation, the sperm samples were spread onto glass slides. Images were examined using a Nikon Apochromat TIRF microscope (Nikon, Tokyo, Japan) and a Hamamatasu C9100-23B EMCCD camera (Hamamatasu, Nikon, Tokyo, Japan). All experiments were performed in biological duplicates.

### Statistical analysis

Data analyses were performed using GraphPad Prism 8.0 Software. Two-tailed unpaired Students’ *t* tests were used to compare the differences between two groups. All data met the assumptions of the tests and are presented as mean ± standard deviation. The data are derived from three independent experiments. Error bars in the figures indicate the standard error of the mean among *n* ≥ 2 biological replicates. A significance level of *P* < 0.05 was considered statistically significant. *P < 0.05, **P < 0.01, ***P < 0.001 and ****P < 0.0001 for all analyses.

## Supplementary information

Supplementary Table

Supplementary Figure

Supplementary Movie 1

Supplementary Movie 2

Supplementary Movie 3

Supplementary Movie 4

Related Manuscript File

## Data Availability

All data are available in the main text or in the supporting information materials. The datasets generated during proteomic analysis are deposited in the publicly available proteomics database ProteomeXchange (http://www.proteomexchange.org, accession number, PXD052758). RNA-seq data have been deposited to GEO database (http://www.ncbi.nlm.nih.gov/geo, accession number, GSE268908). The data that support the findings of this study are available from the corresponding author upon reasonable request.
