# Supplementary material for: TMEM232 is required for the formation of sperm flagellum and male fertility in mice
Source: Cell Death Dis. 2024 Nov 8;15(11):806. doi: 10.1038/s41419-024-07200-9 (PMC11549365; doi:10.1038/s41419-024-07200-9)
Supplement: Supplementary file 1 — Supplementary Table [file 41419_2024_7200_MOESM1_ESM.pdf]

Supplementary Table 1. Semen analysis of *Tmem232*$^{+/+}$, *Tmem232*$^{+/-}$ and *Tmem232*$^{-/-}$ male mice.

Semen parameters	*Tmem232*$^{+/+}$	*Tmem232*$^{+/-}$	*Tmem232*$^{-/-}$
Sperm concentration (10^6/ml)	51.90±17.70	39.40±0.90	18.10±0.80
motile sperm(%)	80.50±11.50	79.00±3.00	0.00±0.00
Progressive motility (PR)(%)	46.00±8.00	49.50±1.50	0.00±0.00
Curvilinear velocity (VCL) (μm/s)	52.59±8.30	56.26±0.20	0.17±0.17
Straight-line velocity (VSL)(μm/s)	16.82±2.18	20.12±0.20	0.00±0.00
Average path velocity (VAP) (μm/s)	27.83±4.33	30.99±0.50	0.05±0.05
Amplitude of lateral head displacement (ALH) (μm)	7.87±1.43	7.92±0.03	0.14±0.14
Beat-cross frequency (BCF) (Hz)	5.93±0.94	6.17±0.21	0.00±0.00
Linearity (LIN)	0.26±0.03	0.26±0.02	0.00±0.00
Wobble (WOB, = VAP/VCL)	0.44±0.06	0.44±0.02	0.01±0.01
Straightness (STR, = VSL/VAP)	0.46±0.06	0.46+0.11	0.00±0.00

Supplementary Table 2. The differentially expressed genes in *Tmem232*$^{-/-}$ and *Tmem232*$^{+/+}$ mice testis.

No.	Gene Symbol	Gene ID	Fold-change	*P*-value	style
1	Gm21454	100862075	-21.02	1.66E-08	down
2	Prn	111368	-5.25	6.15E-03	down
3	LOC108168551	108168551	-4.86	2.51E-02	down
4	LOC108168557	108168557	-1.72	2.44E-02	down
5	Gm13305	100042555	-1.61	4.81E-02	down
6	Fam78b	226610	-1.48	4.12E-02	down
7	Stc1	20855	-1.28	1.78E-04	down
8	Mapk13	26415	-1.15	3.34E-03	down
9	Adamts8	30806	-1.15	3.63E-02	down
10	Kcnk3	16527	-1.09	4.61E-04	down
11	Gm43263	108167571	1.00	3.06E-06	up
12	Map2	17756	1.00	8.05E-13	up
13	Celf3	78784	1.00	2.63E-09	up
14	Gm38564	102641557	1.00	8.32E-16	up
15	Fam122c	73866	1.00	3.09E-05	up
16	1700024J04Rik	105244158	1.01	5.93E-07	up
17	D11Wsu47e	276852	1.01	4.91E-20	up
18	Gm8700	667552	1.01	3.07E-04	up
19	Fam217a	71864	1.01	4.14E-10	up
20	Slc35g3	56293	1.02	1.35E-09	up
21	Arl13a	74448	1.02	3.59E-04	up
22	4921501E09Rik	74042	1.02	1.22E-13	up
23	Klkb1	16621	1.02	1.66E-04	up
24	Muc16	73732	1.02	1.54E-10	up
25	Unc80	329178	1.02	1.69E-04	up
26	Pdzk1ip1	67182	1.02	4.76E-04	up
27	1700016D06Rik	76413	1.02	3.04E-07	up
28	Arhgef2	16800	1.02	2.95E-24	up
29	Luzp1	269593	1.02	6.45E-26	up
30	Grin3b	170483	1.02	1.01E-02	up
31	Gm6744	627311	1.02	3.11E-07	up
32	Otop1	21906	1.02	2.90E-02	up
33	Cd59b	333883	1.02	2.66E-02	up
34	Grm1	14816	1.03	7.84E-05	up
35	Pde1a	18573	1.03	3.78E-11	up
36	Tcp11x2	71841	1.03	3.70E-12	up
37	Amph	218038	1.03	7.33E-05	up
38	Prox2	73422	1.03	1.52E-12	up
39	Gm12206	105246868	1.03	9.11E-10	up
40	Gm14781	629542	1.03	2.15E-06	up

41	1700017N19Rik	66605	1.03	2.05E-09	up
42	Gm9495	670593	1.03	1.97E-08	up
43	Cfhr1	50702	1.03	1.15E-02	up
44	Cib4	73259	1.03	1.29E-05	up
45	Samt2	434881	1.03	1.15E-02	up
46	Klk1b21	16616	1.03	2.19E-02	up
47	Spaca4	69363	1.03	3.31E-06	up
48	Cpsf4l	52670	1.04	2.78E-03	up
49	Myh15	667772	1.04	5.32E-06	up
50	Fam187a	66784	1.04	1.40E-04	up
51	LOC108167340	108167340	1.04	2.33E-04	up
52	Stox2	71069	1.04	2.68E-11	up
53	Gpr15	71223	1.04	6.19E-03	up
54	Myo18b	74376	1.04	4.50E-12	up
55	4930578I06Rik	67750	1.04	1.15E-06	up
56	Tmc5	74424	1.04	4.02E-08	up
57	Serp2	72661	1.05	3.84E-03	up
58	LOC108168945	108168945	1.05	3.82E-03	up
59	Necab3	56846	1.05	5.36E-06	up
60	Tex45	76406	1.05	2.58E-07	up
61	Cdkl5	382253	1.05	2.31E-12	up
62	Pnmal2	434128	1.05	5.11E-21	up
63	Lipe	16890	1.05	3.02E-22	up
64	Scube2	56788	1.05	1.13E-10	up
65	Isg20	57444	1.05	5.92E-08	up
66	Lyzl4	69032	1.05	5.19E-06	up
67	Cpeb3	208922	1.05	6.64E-16	up
68	Dnmt3l	54427	1.05	9.81E-07	up
69	Tssk1	22114	1.06	8.18E-12	up
70	Satl1	73809	1.06	2.13E-06	up
71	Gm5941	546347	1.06	3.09E-05	up
72	4930415O20Rik	73863	1.06	1.28E-08	up
73	Adgrl3	319387	1.06	1.87E-12	up
74	Ankef1	319196	1.06	3.11E-20	up
75	Tex38	75173	1.06	9.20E-06	up
76	Nipsnap3a	73398	1.06	2.98E-09	up
77	Mgam	232714	1.06	4.70E-05	up
78	Theg	21830	1.06	1.81E-10	up
79	Gm21095	100861637	1.07	1.48E-05	up
80	Pkp1	18772	1.07	3.24E-02	up
81	Atp6v1b1	110935	1.07	3.69E-04	up
82	Frat1	14296	1.07	4.22E-11	up
83	4921511M17Rik	667256	1.07	4.50E-04	up
84	Samt1	78092	1.07	4.50E-04	up

85	Cast	12380	1.07	1.68E-13	up
86	Gm5945	546368	1.07	4.73E-02	up
87	Tssk4	71099	1.07	1.13E-10	up
88	Gm21242	100861817	1.07	2.03E-05	up
89	Aox3	71724	1.07	9.05E-14	up
90	Olfr701	66786	1.07	4.20E-08	up
91	Ell2	192657	1.07	1.16E-16	up
92	Sox5	20678	1.07	1.20E-11	up
93	Ank2	109676	1.08	1.76E-20	up
94	Mfap3l	71306	1.08	1.50E-11	up
95	4930480E11Rik	74910	1.08	6.68E-05	up
96	LOC102635990	102635990	1.08	1.94E-25	up
97	Amer2	72125	1.08	2.54E-09	up
98	1110017D15Rik	73721	1.08	1.69E-05	up
99	Pkhd1l1	192190	1.08	1.12E-02	up
100	Fam71e1	75538	1.08	3.23E-07	up
101	Ntrk3	18213	1.08	2.10E-05	up
102	Slc44a5	242259	1.08	1.83E-05	up
103	LOC108168625	108168625	1.08	1.76E-08	up
104	Azin2	242669	1.08	3.49E-05	up
105	Tex36	73808	1.08	2.11E-11	up
106	Cnbd2	70873	1.08	1.12E-19	up
107	Il31	76399	1.08	6.02E-09	up
108	Catsper4	329954	1.08	4.39E-10	up
109	Arl11	219144	1.08	9.52E-04	up
110	Nsun4	72181	1.09	5.29E-19	up
111	Wdr86	269633	1.09	2.90E-04	up
112	Cldn34-ps	628893	1.09	6.92E-03	up
113	Tex26	75860	1.09	9.39E-09	up
114	Btg1-ps1	436199	1.09	1.91E-03	up
115	Fbxl13	320118	1.09	5.41E-21	up
116	Kcnj6	16522	1.09	5.06E-09	up
117	Skp2	27401	1.09	3.97E-15	up
118	Gm4907	236749	1.10	2.56E-07	up
119	Efcab9	69306	1.10	4.61E-08	up
120	Spaca1	67652	1.10	3.59E-14	up
121	Ell3	269344	1.10	8.94E-04	up
122	Spaca7	78634	1.10	2.50E-06	up
123	Atp6v1e2	74915	1.10	1.13E-10	up
124	Kif17	16559	1.10	4.86E-19	up
125	Itga11	319480	1.10	4.19E-05	up
126	Prr27	73779	1.10	5.70E-16	up
127	Ace	11421	1.10	3.39E-25	up
128	Fam196b	574403	1.11	5.63E-03	up

129	Pfn3	75477	1.11	1.89E-03	up
130	Olfr1383	404337	1.11	7.90E-03	up
131	4930558K02Rik	75368	1.11	6.08E-06	up
132	Gm7358	664831	1.11	1.36E-09	up
133	Mroh7	381538	1.12	1.38E-12	up
134	Cldnd2	74276	1.12	2.95E-06	up
135	Usp25	30940	1.12	2.89E-23	up
136	Efhc2	74405	1.12	1.03E-12	up
137	Pcdhgc5	93708	1.12	1.16E-02	up
138	Muc4	140474	1.12	1.39E-11	up
139	Tssk5	73542	1.12	2.65E-07	up
140	Gpx6	75512	1.12	1.14E-05	up
141	Kcne3	57442	1.12	1.14E-08	up
142	Spata7	104871	1.13	2.64E-16	up
143	Phlpp1	98432	1.13	1.08E-19	up
144	Ddi1	71829	1.13	6.34E-19	up
145	Ankrd61	66729	1.13	4.15E-24	up
146	Dgkh	380921	1.13	3.15E-18	up
147	Smim9	434800	1.13	2.06E-02	up
148	Ccer1	66716	1.13	2.94E-09	up
149	1700067P10Rik	68224	1.13	2.53E-07	up
150	Gm5681	435509	1.13	1.12E-04	up
151	Odf3l1	382075	1.13	1.31E-11	up
152	Gm32970	102635699	1.13	1.23E-02	up
153	Spata48	73862	1.13	2.76E-09	up
154	Phospho1	237928	1.13	4.17E-06	up
155	Tmem269	75180	1.13	2.66E-10	up
156	Tescl	69301	1.13	4.61E-07	up
157	Cdh12	215654	1.13	7.71E-05	up
158	Proca1	216974	1.13	6.05E-07	up
159	Mapt	17762	1.13	9.88E-03	up
160	Arhgef6	73341	1.13	8.12E-12	up
161	Cpa6	329093	1.14	1.64E-03	up
162	Vmn2r122	22308	1.14	2.36E-03	up
163	Gm7356	664821	1.14	1.66E-06	up
164	Fam110a	73847	1.14	1.11E-15	up
165	Btg1-ps2	194735	1.14	1.97E-06	up
166	Aldoart2	79459	1.14	6.89E-13	up
167	Ccdc81	70884	1.14	3.86E-26	up
168	Dusp4	319520	1.14	1.92E-06	up
169	Asb9	69299	1.14	9.04E-17	up
170	Plekha6	240753	1.14	2.85E-20	up
171	Gstt4	75886	1.14	1.04E-08	up
172	Klc3	232943	1.15	2.69E-02	up

173	P2rx3	228139	1.15	3.59E-06	up
174	Fam131b	76156	1.15	3.25E-02	up
175	Btbd35f5	100040533	1.15	1.59E-03	up
176	Ccdc63	330188	1.15	1.36E-16	up
177	Vwa5b1	75718	1.15	1.15E-13	up
178	Gm10634	100039674	1.15	2.04E-12	up
179	Adam26b	382007	1.15	5.77E-06	up
180	4430402I18Rik	381218	1.15	1.57E-17	up
181	Tmem249	666504	1.15	3.76E-04	up
182	Lrrc72	71156	1.15	5.83E-09	up
183	Tmem190	78052	1.15	1.55E-07	up
184	Slit1	20562	1.15	1.70E-02	up
185	Zfp474	66758	1.15	1.73E-13	up
186	Ttc39a	230603	1.16	3.77E-18	up
187	Ppef1	237178	1.16	1.19E-09	up
188	Scn7a	20272	1.16	4.99E-03	up
189	Gm732	213450	1.16	3.62E-09	up
190	Gm7168	635895	1.16	4.10E-08	up
191	Zp3r	22789	1.16	1.33E-17	up
192	Apoh	11818	1.16	8.97E-11	up
193	Efcab12	212516	1.16	3.79E-11	up
194	Tmem144	70652	1.16	1.50E-09	up
195	Spam1	20690	1.16	5.85E-16	up
196	Rai2	24004	1.17	3.75E-05	up
197	Fer1l6	631797	1.17	6.91E-03	up
198	Btbd35f23	100861966	1.17	1.28E-03	up
199	Cfhr3	624286	1.17	2.53E-05	up
200	Llcfc1	76606	1.17	3.10E-08	up
201	Myo1h	231646	1.17	3.57E-02	up
202	Spata46	76925	1.17	5.57E-11	up
203	Adtrp	109254	1.17	1.13E-04	up
204	Samt4	75185	1.17	2.56E-04	up
205	Olfr128	383243	1.18	1.06E-02	up
206	Gm16445	635580	1.18	4.16E-05	up
207	Fam221b	242408	1.18	6.50E-18	up
208	Gm46500	108168219	1.18	8.54E-07	up
209	1700061G19Rik	78625	1.19	2.60E-18	up
210	Izumo1	73456	1.19	1.03E-09	up
211	Fam229a	68233	1.19	5.06E-06	up
212	Triml1	244448	1.19	8.81E-15	up
213	Acsl1	14081	1.19	4.68E-28	up
214	Adam39	546055	1.19	3.09E-18	up
215	Dnah9	237806	1.19	8.15E-04	up
216	Gm21118	100861667	1.19	2.07E-02	up

217	Slc7a11	26570	1.19	5.05E-05	up
218	Adam26a	13525	1.19	1.70E-12	up
219	Sh3kbp1	58194	1.19	2.04E-07	up
220	Znrf4	20834	1.19	8.40E-10	up
221	Tmod1	21916	1.20	2.82E-06	up
222	Arrdc5	76920	1.20	1.34E-11	up
223	Col22a1	69700	1.20	4.50E-10	up
224	4933405O20Rik	243996	1.20	7.81E-14	up
225	Ppm1j	71887	1.20	5.49E-14	up
226	Ms4a5	269063	1.20	1.72E-12	up
227	Selenov	280621	1.20	6.89E-14	up
228	Spata20	217116	1.20	2.59E-25	up
229	Srcin1	56013	1.20	5.04E-22	up
230	Prox1	19130	1.20	3.13E-09	up
231	Acp4	100503991	1.20	1.51E-13	up
232	Ttc23l	75777	1.20	1.79E-15	up
233	Casp1	12362	1.20	4.99E-02	up
234	4930522H14Rik	67646	1.20	5.24E-08	up
235	BC049762	193286	1.20	2.66E-13	up
236	LOC108168946	108168946	1.21	1.44E-02	up
237	Klk1b27	16619	1.21	3.14E-03	up
238	Btbd35f21	100862329	1.21	1.89E-04	up
239	Tbata	65971	1.21	9.73E-16	up
240	Eva1c	70967	1.21	2.91E-07	up
241	Hist1h4a	326619	1.22	1.08E-04	up
242	Ston1	77057	1.22	2.94E-09	up
243	Cldn34c4	73934	1.22	2.45E-06	up
244	Pknox2	208076	1.22	1.21E-21	up
245	Ace3	217246	1.22	1.82E-15	up
246	Actg2	11468	1.22	1.83E-10	up
247	Fam209	76426	1.22	5.58E-08	up
248	Cep295nl	58251	1.22	2.41E-11	up
249	Paqr9	75552	1.22	3.35E-19	up
250	Creb3l4	78284	1.22	1.45E-11	up
251	H2al1g	100042943	1.22	1.79E-03	up
252	Acrv1	11451	1.22	4.59E-14	up
253	Spata21	329972	1.22	3.62E-24	up
254	Tsga13	116732	1.22	2.46E-08	up
255	Cabyr	71132	1.23	7.52E-20	up
256	1700113H08Rik	76640	1.23	1.96E-22	up
257	Adam20	384806	1.23	4.12E-23	up
258	Fam183b	75429	1.23	4.32E-07	up
259	Lyzl1	67328	1.23	5.80E-11	up
260	Glrp1	14659	1.23	2.92E-02	up

261	Zcchc13	75064	1.23	4.92E-15	up
262	Izumo3	69314	1.23	4.50E-15	up
263	Tmem262	433215	1.23	1.01E-05	up
264	Armc12	67645	1.23	4.19E-10	up
265	Tas1r1	110326	1.23	1.73E-09	up
266	1700011L22Rik	67687	1.23	1.04E-13	up
267	Trim17	56631	1.23	2.77E-17	up
268	Tex13a	67944	1.23	1.15E-15	up
269	Ccdc70	67929	1.23	8.74E-07	up
270	4930505A04Rik	75087	1.23	7.01E-07	up
271	Eya4	14051	1.23	8.55E-20	up
272	Cfap100	243538	1.23	1.62E-09	up
273	Dpysl3	22240	1.23	4.44E-29	up
274	Txndc8	67402	1.23	3.31E-08	up
275	Adam25	23793	1.23	1.46E-18	up
276	Spta1	20739	1.23	5.05E-06	up
277	1700019N19Rik	67507	1.24	5.11E-11	up
278	Gm8765	667693	1.24	6.99E-06	up
279	Gpat3	231510	1.24	7.83E-06	up
280	Serpina3n	20716	1.24	2.83E-02	up
281	2900092C05Rik	73090	1.24	5.67E-07	up
282	Iqcm	71831	1.24	9.51E-13	up
283	Nt5c1b	70881	1.24	8.66E-22	up
284	Zdhhc19	245308	1.24	2.71E-09	up
285	Gzmn	245839	1.24	1.25E-08	up
286	Nyx	236690	1.24	3.02E-02	up
287	Acoxl	74121	1.24	8.76E-06	up
288	P2ry14	140795	1.24	4.92E-02	up
289	1700013G24Rik	69380	1.24	9.43E-19	up
290	Tmem56	99887	1.25	1.60E-10	up
291	Plcz1	114875	1.25	8.63E-21	up
292	Lrrc8b	433926	1.25	2.00E-32	up
293	1700013F07Rik	75504	1.25	7.66E-07	up
294	Fam24b	69318	1.25	6.32E-13	up
295	Iqcf5	75470	1.25	3.30E-06	up
296	Ankrd9	74251	1.25	8.22E-05	up
297	Grip1	74053	1.26	1.62E-19	up
298	Nectin4	71740	1.26	7.38E-03	up
299	Tex33	73376	1.26	3.31E-11	up
300	Gm5346	384813	1.26	1.70E-05	up
301	4930523C07Rik	67647	1.26	4.09E-12	up
302	Trim66	330627	1.26	1.01E-16	up
303	Fam83e	73813	1.26	4.74E-16	up
304	A630023A22Rik	105518	1.26	1.23E-03	up

305	1700003F12Rik	75480	1.26	1.76E-07	up
306	Lpcat2b	70902	1.26	1.29E-24	up
307	Tex43	67343	1.26	1.00E-05	up
308	Bcl2l14	66813	1.26	2.79E-19	up
309	Lrrc37a	237954	1.26	9.14E-16	up
310	4931428F04Rik	74356	1.26	1.90E-14	up
311	Dcst1	77772	1.27	3.21E-16	up
312	Apobec4	71281	1.27	5.39E-12	up
313	Cylc2	74914	1.27	2.21E-03	up
314	Dusp13	27389	1.27	5.60E-11	up
315	St8sia3	20451	1.27	1.75E-14	up
316	Spag4	245865	1.27	6.99E-29	up
317	Slc22a14	382113	1.27	1.70E-13	up
318	Dnah3	381917	1.28	5.91E-18	up
319	Adipoq	11450	1.28	1.34E-04	up
320	Sun5	76407	1.28	7.17E-11	up
321	Stat4	20849	1.28	1.35E-24	up
322	Cylc1	67407	1.28	1.34E-08	up
323	2810408A11Rik	70419	1.28	8.98E-11	up
324	Fabp12	75497	1.28	8.64E-07	up
325	Prkcd	18753	1.28	7.64E-12	up
326	Aldoart1	353204	1.28	2.87E-15	up
327	Crispld1	83691	1.28	1.08E-02	up
328	Sgms2	74442	1.28	1.15E-16	up
329	1700029I15Rik	75641	1.28	1.04E-05	up
330	Zdhhc25	70073	1.29	2.24E-10	up
331	Pcdh9	211712	1.29	4.86E-02	up
332	1700057G04Rik	78459	1.29	1.25E-19	up
333	Acsbg2	328845	1.29	2.81E-16	up
334	Gm20929	100042578	1.29	1.08E-04	up
335	Catsper3	76856	1.29	8.66E-22	up
336	Tex48	75524	1.29	3.30E-08	up
337	Fam71e2	243822	1.29	4.56E-11	up
338	1700015F17Rik	381716	1.29	2.64E-11	up
339	Wscd2	320916	1.30	3.80E-04	up
340	Prm3	19120	1.30	4.70E-04	up
341	Pmfbp1	56523	1.30	2.98E-20	up
342	Ubqln3	244178	1.30	4.33E-45	up
343	Gm20903	100042110	1.30	2.14E-05	up
344	Spata9	75571	1.30	1.41E-08	up
345	Irgc1	210145	1.30	2.70E-12	up
346	LOC102638085	102638085	1.31	4.30E-17	up
347	Rbm43	71684	1.31	1.10E-12	up
348	Actl10	70362	1.31	1.76E-08	up

349	Tssk3	58864	1.31	7.00E-08	up
350	Adam1a	280668	1.31	3.82E-15	up
351	Lrrc74a	627607	1.31	1.22E-13	up
352	Cpa5	74649	1.31	8.10E-19	up
353	Tex13c1	637093	1.31	5.82E-13	up
354	Ankrd53	75305	1.31	1.49E-24	up
355	Actrt1	73360	1.31	8.35E-08	up
356	Stambpl1	76630	1.31	1.11E-13	up
357	Pitx2	18741	1.31	6.13E-03	up
358	Ms4a14	383435	1.31	6.66E-23	up
359	Gm20153	100504292	1.31	2.25E-10	up
360	Ccdc33	382077	1.31	2.18E-12	up
361	Ccdc183	77058	1.31	1.69E-09	up
362	Dydc2	71200	1.31	1.50E-03	up
363	Nxph1	18231	1.31	4.90E-02	up
364	Igsf9	93842	1.32	2.76E-07	up
365	1700109H08Rik	77036	1.32	5.35E-08	up
366	Csrnp3	77771	1.32	4.34E-10	up
367	Fam135b	70363	1.32	2.02E-12	up
368	1700010D01Rik	76386	1.33	3.98E-07	up
369	4931409K22Rik	231045	1.33	1.19E-09	up
370	Arl9	384185	1.33	5.48E-10	up
371	Olfr112	258096	1.33	2.29E-07	up
372	Adam24	13526	1.33	8.63E-21	up
373	Gm6729	627035	1.33	8.45E-12	up
374	Sqor	59010	1.33	5.91E-17	up
375	Allc	94041	1.34	7.88E-18	up
376	Cypt2	245566	1.34	1.27E-07	up
377	Chrnb4	108015	1.34	3.45E-02	up
378	Btbd35f25	100862274	1.34	6.29E-05	up
379	Eqtn	67753	1.34	2.13E-14	up
380	1700012B07Rik	69324	1.34	6.73E-16	up
381	Nim1k	245269	1.34	2.24E-04	up
382	Apol7b	278679	1.34	1.31E-03	up
383	Ctag2	70062	1.34	2.86E-11	up
384	Tmem210	78217	1.34	8.74E-09	up
385	Odf1	18285	1.35	1.97E-16	up
386	Pdilt	71830	1.35	3.90E-19	up
387	Rab3il1	74760	1.35	6.02E-41	up
388	Ccdc91	67015	1.35	8.80E-19	up
389	G6pd2	14380	1.35	6.07E-14	up
390	Catsper1	225865	1.35	3.37E-26	up
391	Izumo2	75510	1.36	5.10E-15	up
392	Gtsf1l	68236	1.36	5.91E-14	up

393	Cpxcr1	382239	1.36	3.67E-12	up
394	4930564C03Rik	75341	1.36	4.98E-10	up
395	Prkar2a	19087	1.36	7.37E-20	up
396	Cldn34c2	625591	1.36	2.81E-03	up
397	4921539E11Rik	70941	1.36	5.23E-25	up
398	Tmco5	67356	1.36	1.71E-12	up
399	Gpr158	241263	1.36	2.39E-04	up
400	Lyzl6	69444	1.37	1.05E-21	up
401	Akap3	11642	1.37	9.64E-34	up
402	Dcdc2c	68511	1.37	7.14E-08	up
403	Best1	24115	1.37	1.54E-13	up
404	Sectm1a	209588	1.37	2.79E-02	up
405	Gm5646	434879	1.37	1.80E-06	up
406	Wnt3a	22416	1.37	1.53E-02	up
407	Tmem247	78469	1.38	8.25E-10	up
408	4933402J07Rik	330820	1.38	2.14E-11	up
409	Lrrc18	67580	1.38	3.35E-23	up
410	Cd37	12493	1.38	1.20E-03	up
411	Chl1	12661	1.38	4.23E-25	up
412	Chd5	269610	1.38	8.99E-30	up
413	H2al1e	547160	1.38	1.41E-04	up
414	BC048671	243535	1.38	2.17E-11	up
415	Actl7a	11470	1.38	7.91E-24	up
416	Pla2g10	26565	1.38	1.87E-07	up
417	Asb15	78910	1.39	1.31E-15	up
418	Prkcq	18761	1.39	1.26E-25	up
419	Smcp	17235	1.39	1.94E-04	up
420	Dnajc5b	66326	1.39	8.08E-16	up
421	Cdyl	12593	1.39	5.06E-43	up
422	Otub2	68149	1.40	1.20E-20	up
423	Clip4	78785	1.40	3.35E-29	up
424	1700009N14Rik	75471	1.40	1.31E-15	up
425	Lrrc52	240899	1.40	8.78E-13	up
426	Gm15104	333588	1.40	1.89E-08	up
427	Ccsap	73420	1.40	5.42E-11	up
428	Ceacam2	26367	1.40	8.99E-12	up
429	Fat3	270120	1.40	8.35E-08	up
430	Abhd2	54608	1.40	1.04E-36	up
431	Klf5	12224	1.40	3.35E-05	up
432	Gadl1	73748	1.40	1.46E-03	up
433	4933436I01Rik	66780	1.41	1.15E-06	up
434	Ropn1	76378	1.41	1.37E-23	up
435	Tbc1d21	74286	1.41	8.97E-12	up
436	4930404N11Rik	432479	1.41	4.56E-07	up

437	Gm9831	101056032	1.41	4.18E-25	up
438	4933417A18Rik	66761	1.41	9.43E-14	up
439	Cngb3	30952	1.41	1.21E-07	up
440	Gsg1	14840	1.41	4.40E-29	up
441	Mbd3l1	73503	1.41	3.68E-07	up
442	Ube2dnl1	237009	1.42	5.36E-04	up
443	Dsg1b	225256	1.42	6.14E-06	up
444	Usp50	75083	1.42	1.46E-14	up
445	Ttll10	330010	1.42	2.43E-14	up
446	Iqcn	637079	1.42	8.57E-18	up
447	Spz1	79401	1.42	9.43E-14	up
448	Vav3	57257	1.42	4.54E-08	up
449	Aqp7	11832	1.42	9.43E-14	up
450	Gpr39	71111	1.42	1.18E-03	up
451	Pp2d1	110332	1.43	5.77E-20	up
452	4930596D02Rik	239036	1.43	2.68E-06	up
453	Ces1d	104158	1.43	6.45E-03	up
454	Pdzk1	59020	1.44	3.12E-21	up
455	Gm7073	631784	1.44	4.84E-14	up
456	Rhox11	194738	1.44	2.97E-03	up
457	Tsks	22116	1.44	3.53E-12	up
458	Chrna9	231252	1.44	2.91E-04	up
459	1700014D04Rik	102638268	1.44	9.53E-27	up
460	4930568D16Rik	75859	1.44	4.12E-05	up
461	1700001P01Rik	72215	1.44	4.20E-06	up
462	Cypt3	69361	1.44	5.93E-11	up
463	Tuba4a	22145	1.45	6.66E-17	up
464	Apoc1	11812	1.45	9.65E-03	up
465	Cypt12	75439	1.45	1.70E-06	up
466	Gm5347	384814	1.45	5.76E-04	up
467	Olfr1508	57270	1.45	3.46E-02	up
468	St6galnac2	20446	1.45	7.60E-21	up
469	Atp8a2	50769	1.45	3.21E-21	up
470	Spata31	78124	1.45	2.37E-19	up
471	Kif2b	73470	1.45	2.45E-24	up
472	Prr30	76627	1.45	1.83E-28	up
473	Capza3	12344	1.46	3.39E-20	up
474	Odf3	69287	1.46	4.76E-10	up
475	Cypt1	66742	1.47	2.18E-16	up
476	Sept4	18952	1.47	6.38E-32	up
477	Rnf151	67504	1.47	7.65E-17	up
478	4930548H24Rik	67656	1.47	1.01E-06	up
479	4930451I11Rik	78118	1.47	6.14E-04	up
480	Iqcf4	67320	1.47	5.01E-09	up

481	Fam187b	76415	1.47	3.74E-15	up
482	Spaca9	69987	1.47	8.35E-13	up
483	Akap1	11640	1.48	6.53E-36	up
484	Gm10057	625650	1.48	3.86E-06	up
485	Sh3d21	66938	1.48	1.20E-17	up
486	4930513O06Rik	75125	1.48	9.38E-06	up
487	Tmem191c	224019	1.49	1.17E-10	up
488	Tspan6	56496	1.49	9.05E-14	up
489	Gm4275	102636907	1.49	1.35E-08	up
490	Tmco2	69469	1.49	8.23E-11	up
491	Brinp3	215378	1.50	4.96E-06	up
492	Erich3	209601	1.50	6.91E-32	up
493	Hsfy2	71066	1.50	6.50E-14	up
494	Stk-ps1	545204	1.50	4.38E-07	up
495	Gm4984	245347	1.50	2.59E-05	up
496	Sncaip	67847	1.50	1.16E-15	up
497	Btbd35f11	668958	1.50	9.69E-05	up
498	Lmntd1	74071	1.51	1.70E-16	up
499	Gapdhs	14447	1.51	3.48E-15	up
500	Trem1	58217	1.51	4.74E-04	up
501	Hils1	54388	1.51	1.72E-21	up
502	Dusp10	63953	1.51	5.03E-12	up
503	Btbd35f22	100862323	1.51	1.69E-02	up
504	Pwwp2b	101631	1.51	1.35E-40	up
505	Vmn2r5	667060	1.51	1.23E-02	up
506	Zscan5b	170734	1.51	2.04E-02	up
507	Hyal5	74468	1.52	1.48E-28	up
508	Gm33242	102636069	1.52	1.98E-03	up
509	Egr3	13655	1.52	1.07E-16	up
510	Fscb	623046	1.53	2.14E-32	up
511	Cd96	84544	1.53	6.96E-06	up
512	Gpd2	14571	1.53	9.50E-36	up
513	2610318N02Rik	70458	1.53	1.83E-29	up
514	Bco1	63857	1.53	2.09E-11	up
515	Hdac11	232232	1.54	5.02E-14	up
516	Pkib	18768	1.54	1.10E-15	up
517	Pcdhgb5	93702	1.54	1.94E-02	up
518	Adam34	252866	1.55	5.62E-10	up
519	Adam29	244486	1.55	3.11E-19	up
520	Camk4	12326	1.55	3.71E-10	up
521	Adam21	56622	1.55	2.16E-14	up
522	Xirp1	22437	1.55	1.15E-02	up
523	Proc	19123	1.55	5.64E-03	up
524	Spata19	75469	1.55	3.36E-15	up

525	Dpp6	13483	1.56	4.47E-04	up
526	Rassf9	237504	1.56	7.22E-09	up
527	BC048507	408058	1.56	1.54E-10	up
528	Fam205c	277773	1.56	1.64E-20	up
529	Gm11780	622019	1.57	3.74E-17	up
530	Csnka2ip	224291	1.57	5.64E-23	up
531	Antxrl	239029	1.57	4.13E-19	up
532	4930474N05Rik	218921	1.57	2.51E-07	up
533	2200002J24Rik	69147	1.58	5.27E-05	up
534	Gm382	211208	1.58	4.59E-14	up
535	Slc25a41	103775	1.58	2.26E-04	up
536	Gm20852	100040786	1.58	2.35E-05	up
537	Gm6657	626215	1.58	1.76E-12	up
538	Fam81b	238726	1.58	2.96E-15	up
539	Fhdc1	229474	1.58	1.86E-14	up
540	Gm21119	100861668	1.58	1.14E-10	up
541	1700093K21Rik	67358	1.58	3.39E-17	up
542	Gm5645	434874	1.59	5.93E-04	up
543	Cntnap5c	620292	1.59	1.30E-02	up
544	Chrm4	12672	1.59	3.71E-05	up
545	Tssk2	22115	1.59	2.20E-24	up
546	Ccdc27	381580	1.60	1.16E-09	up
547	Klf17	75753	1.60	1.13E-11	up
548	Stpg3	74472	1.60	4.31E-09	up
549	Cdc14a	229776	1.60	7.88E-20	up
550	Samt3	73495	1.60	2.20E-04	up
551	1700019D03Rik	67080	1.60	3.60E-12	up
552	Cep85l	100038725	1.60	4.19E-44	up
553	4930488N24Rik	75011	1.60	5.98E-09	up
554	1700023F06Rik	69441	1.60	1.58E-20	up
555	Dsg1a	13510	1.62	6.95E-12	up
556	Gm5947	546397	1.62	2.64E-04	up
557	Fam71d	70897	1.62	5.42E-39	up
558	Cntn5	244682	1.62	2.84E-02	up
559	Srp54b	665155	1.62	7.65E-17	up
560	Oaz3	53814	1.63	2.06E-14	up
561	Gm6812	627927	1.63	1.60E-10	up
562	Aif1	11629	1.64	3.40E-11	up
563	1700056E22Rik	73363	1.64	1.96E-10	up
564	Spata18	73472	1.64	1.92E-43	up
565	B4galnt4	330671	1.65	1.12E-04	up
566	1700020N15Rik	75509	1.65	6.94E-10	up
567	Dlx5	13395	1.65	1.71E-02	up
568	Wdr49	213248	1.66	1.83E-02	up

569	Pkd1l3	244646	1.66	1.23E-12	up
570	Ccr4	12773	1.66	7.92E-03	up
571	Cypt4	235067	1.66	1.86E-06	up
572	Vwa3a	233813	1.66	2.23E-15	up
573	Ifi209	236312	1.67	2.70E-08	up
574	Ccdc187	329366	1.67	2.43E-16	up
575	Pcsk2	18549	1.67	1.67E-02	up
576	Sstr3	20607	1.67	3.82E-03	up
577	Gm36807	102640833	1.67	3.18E-04	up
578	Fbxo2	230904	1.67	3.65E-06	up
579	Ccdc54	69339	1.68	5.89E-28	up
580	H2al1a	100042922	1.68	1.19E-06	up
581	4931429L15Rik	74361	1.68	1.18E-28	up
582	Tex29	75528	1.68	1.39E-03	up
583	Scgn	214189	1.68	2.87E-04	up
584	Ccdc169	320604	1.68	8.05E-13	up
585	Pax5	18507	1.69	9.55E-12	up
586	Plb1	665270	1.69	6.21E-14	up
587	Akap4	11643	1.69	1.60E-36	up
588	Gm6713	626858	1.69	1.94E-19	up
589	Heatr9	629303	1.69	6.57E-16	up
590	Traf1	22029	1.70	9.94E-05	up
591	Morn3	74890	1.70	8.44E-12	up
592	Trim36	28105	1.71	2.32E-33	up
593	Gm35586	102639227	1.71	2.27E-03	up
594	H2al1b	100042927	1.72	1.95E-08	up
595	Fam71f1	330277	1.73	9.68E-28	up
596	Slco5a1	240726	1.73	3.34E-03	up
597	Gm14725	628053	1.73	4.78E-07	up
598	Galntl5	67909	1.73	4.22E-32	up
599	Samd4	74480	1.73	2.43E-16	up
600	1700029H14Rik	66501	1.74	3.04E-18	up
601	Prss58	232717	1.74	4.06E-12	up
602	Osgin1	71839	1.76	1.08E-18	up
603	Fbxo24	71176	1.76	1.06E-24	up
604	Gm14147	381390	1.78	6.94E-05	up
605	Sgip1	73094	1.78	1.81E-05	up
606	Etv2	14008	1.78	5.35E-03	up
607	1700122O11Rik	76651	1.79	4.08E-07	up
608	1700013D24Rik	76921	1.79	3.63E-05	up
609	Mgat4e	71001	1.79	9.87E-18	up
610	Wnt3	22415	1.79	1.81E-04	up
611	Gm20894	108168547	1.80	3.67E-09	up
612	Fam71f2	245884	1.80	2.96E-16	up

613	LOC108168567	108168567	1.80	2.01E-02	up
614	BC048562	434439	1.80	5.48E-09	up
615	Noxred1	71275	1.81	3.33E-06	up
616	Iqcf3	68265	1.81	3.56E-14	up
617	Scp2d1	66328	1.81	1.08E-14	up
618	Gm5932	546268	1.81	3.89E-03	up
619	Slc5a4b	64454	1.82	2.76E-09	up
620	4930571K23Rik	75861	1.83	6.32E-19	up
621	Gm4994	245600	1.83	1.39E-11	up
622	4930503B20Rik	75015	1.84	1.27E-17	up
623	1700001C19Rik	75462	1.84	3.22E-11	up
624	Pnp2	667034	1.84	2.21E-05	up
625	Pabpc1l	381404	1.84	3.05E-03	up
626	LOC100504180	100504180	1.85	2.30E-06	up
627	Gm39566	105243794	1.86	1.36E-33	up
628	Ndufa11b	239760	1.86	2.34E-02	up
629	Cd46	17221	1.86	5.90E-11	up
630	Fam243	75328	1.87	5.56E-27	up
631	Actl11	67722	1.87	1.02E-33	up
632	Spert	67926	1.87	1.82E-16	up
633	Runx2	12393	1.87	1.63E-05	up
634	Micalcl	100504195	1.88	9.97E-16	up
635	Acot10	64833	1.88	4.44E-09	up
636	Cst8	13012	1.88	6.64E-16	up
637	Tmem156	243025	1.89	7.02E-06	up
638	Spata31d1d	238663	1.89	1.13E-15	up
639	Kctd16	383348	1.90	1.35E-04	up
640	Vmn2r7	319217	1.91	2.35E-03	up
641	Tmem270	76629	1.91	4.08E-09	up
642	Saxo1	75811	1.92	1.59E-14	up
643	Atp1a4	27222	1.92	1.46E-37	up
644	Cfap97d1	75437	1.93	2.79E-10	up
645	Car2	12349	1.93	1.56E-08	up
646	4930453H23Rik	100503481	1.93	3.67E-12	up
647	Tex52	71149	1.94	6.74E-03	up
648	Spata25	75642	1.94	7.93E-08	up
649	Agpat2	67512	1.94	1.68E-13	up
650	1700024P04Rik	69382	1.95	1.24E-09	up
651	Gm3012	105245684	1.95	1.26E-05	up
652	Aknad1	329738	1.95	4.81E-15	up
653	Crb1	170788	1.96	1.19E-04	up
654	Zic5	65100	1.96	4.32E-06	up
655	Hypm	67334	1.96	7.55E-12	up
656	Actrt2	73353	1.97	1.21E-15	up

657	Ppp2r2b	72930	1.97	3.59E-07	up
658	Gm35083	102638546	1.97	4.13E-08	up
659	Kcnh8	211468	1.97	5.21E-07	up
660	Lemd1	213409	1.98	6.34E-20	up
661	Gm4787	214321	1.98	3.93E-29	up
662	Pcdhb21	93892	1.98	2.80E-02	up
663	Gm31309	102633498	1.99	5.68E-05	up
664	Dnajb7	57755	1.99	2.73E-06	up
665	Nwd2	319807	2.00	5.78E-04	up
666	Fam166a	68222	2.01	4.02E-17	up
667	Grid1	14803	2.01	1.65E-06	up
668	Hmgb4	69317	2.01	9.88E-08	up
669	Cdrt4	66338	2.03	1.83E-04	up
670	Ubl4b	67591	2.03	1.10E-15	up
671	D930020B18Rik	216393	2.04	3.75E-05	up
672	Vmn2r4	637053	2.05	2.07E-03	up
673	1700092M07Rik	74307	2.05	3.63E-07	up
674	Grm6	108072	2.06	6.07E-04	up
675	Cyp4f37	677156	2.06	1.70E-02	up
676	H1fnt	70069	2.06	4.77E-07	up
677	Chn2	69993	2.06	1.60E-20	up
678	Gck	103988	2.07	3.53E-06	up
679	Faiml	623459	2.07	6.40E-05	up
680	4933411K16Rik	66765	2.08	3.04E-28	up
681	Clmn	94040	2.09	1.74E-30	up
682	Tex35	73435	2.10	2.22E-07	up
683	Gm13547	433416	2.10	9.36E-05	up
684	2610528A11Rik	70045	2.11	6.48E-09	up
685	Fhl5	57756	2.11	2.64E-62	up
686	Iqcf1	74267	2.11	2.27E-09	up
687	Dusp21	73547	2.12	6.29E-12	up
688	Ly6g6c	68468	2.13	6.82E-08	up
689	Gm14151	433486	2.13	2.71E-19	up
690	Trh	22044	2.15	3.51E-02	up
691	Adam6b	238405	2.15	1.64E-23	up
692	Hspa1l	15482	2.15	4.96E-10	up
693	Eno1b	433182	2.15	1.32E-16	up
694	Hecw1	94253	2.15	1.61E-21	up
695	Ccdc159	67119	2.16	7.43E-23	up
696	Tepp	73407	2.17	1.51E-14	up
697	Tctex1d1	67344	2.18	1.04E-17	up
698	Mroh5	268816	2.18	1.15E-06	up
699	Cd109	235505	2.19	3.93E-29	up
700	Gm609	208166	2.20	5.78E-04	up

701	1700001K19Rik	66323	2.21	2.03E-26	up
702	1700016H13Rik	74218	2.21	5.47E-15	up
703	Slfnl1	194219	2.21	7.60E-21	up
704	Adam4	11498	2.22	4.14E-21	up
705	Calr3	73316	2.23	2.25E-24	up
706	Gm46270	108167883	2.23	1.75E-03	up
707	Cct8l1	242891	2.24	4.01E-18	up
708	Gm21759	102633023	2.26	1.24E-07	up
709	Gm6760	627470	2.26	4.39E-10	up
710	Smok3c	622486	2.26	1.01E-05	up
711	Btbd35f15	100040698	2.27	1.14E-07	up
712	Ccin	442829	2.27	5.68E-30	up
713	Adam6a	238406	2.28	4.31E-41	up
714	Cdhr1	170677	2.28	1.48E-02	up
715	Lgr5	14160	2.28	7.24E-05	up
716	Gm4916	237030	2.28	2.37E-22	up
717	Cypt15	78631	2.29	1.61E-03	up
718	4921528I07Rik	77049	2.30	4.38E-25	up
719	Cldn34c3	382265	2.30	4.76E-07	up
720	Ccdc188	102638083	2.30	5.15E-15	up
721	Asb17	66772	2.30	7.35E-08	up
722	Pcdha1	116731	2.31	1.27E-04	up
723	Adora3	11542	2.31	1.90E-07	up
724	Txndc2	213272	2.31	1.56E-11	up
725	Tnfsf15	326623	2.32	2.92E-02	up
726	Smok2a	27263	2.32	2.10E-09	up
727	Sh3rf2	269016	2.33	2.47E-07	up
728	Rnf148	71300	2.33	2.36E-12	up
729	Cyp2e1	13106	2.34	2.90E-02	up
730	Ccdc168	102636082	2.34	6.08E-29	up
731	Lrrc69	73314	2.34	2.52E-15	up
732	Gm35174	102638664	2.35	3.55E-02	up
733	Rnf133	386611	2.36	6.06E-17	up
734	Dnajb8	56691	2.37	3.99E-07	up
735	Tmigd3	69296	2.37	1.05E-20	up
736	Spata3	70060	2.37	1.74E-08	up
737	Dscaml1	114873	2.38	3.80E-05	up
738	Hrh2	15466	2.38	1.01E-02	up
739	Spem2	108803	2.38	1.96E-17	up
740	Trim80	432613	2.39	3.72E-17	up
741	Banf2	403171	2.39	1.69E-07	up
742	LOC101056115	101056115	2.39	2.97E-14	up
743	4930567H17Rik	619303	2.40	5.15E-09	up
744	Klrb1	100043861	2.40	5.96E-10	up

745	Prss55	71037	2.41	1.56E-17	up
746	Klk1b26	16618	2.43	4.84E-02	up
747	Adra1d	11550	2.43	2.51E-03	up
748	F5	14067	2.44	1.75E-22	up
749	Cntnap5b	241175	2.44	4.63E-16	up
750	Pex5l	58869	2.44	7.60E-15	up
751	Tex50	100502590	2.46	3.63E-11	up
752	Ttll2	100216474	2.46	2.41E-13	up
753	Gapt	238875	2.46	1.05E-03	up
754	4930435E12Rik	74663	2.49	2.84E-24	up
755	Ano2	243634	2.49	2.41E-03	up
756	Trpm8	171382	2.49	1.99E-04	up
757	Gm21379	100861987	2.51	3.61E-15	up
758	Rbm31y	74484	2.51	3.61E-15	up
759	4921509C19Rik	381393	2.52	2.81E-21	up
760	LOC108168521	108168521	2.53	2.02E-02	up
761	Lelp1	69332	2.53	1.84E-25	up
762	Sppl2c	237958	2.55	6.47E-32	up
763	Fndc8	78919	2.58	4.53E-07	up
764	Wdr64	75820	2.58	1.44E-23	up
765	Lrrc4c	241568	2.58	1.13E-02	up
766	1700034E13Rik	78414	2.59	1.60E-15	up
767	Lrrd1	242838	2.61	3.77E-15	up
768	Gm614	245536	2.62	3.80E-09	up
769	Dyrk4	101320	2.64	6.95E-21	up
770	Paqr5	74090	2.64	2.00E-13	up
771	H2al2a	68231	2.65	3.28E-05	up
772	Adig	246747	2.65	1.35E-09	up
773	Atp6v1fnb	668210	2.66	2.75E-10	up
774	4930407I10Rik	328573	2.68	1.24E-35	up
775	Gm29825	102631503	2.68	9.44E-08	up
776	Smim23	69351	2.70	2.15E-16	up
777	4921507P07Rik	70821	2.70	4.19E-44	up
778	Ccdc185	433386	2.70	6.45E-07	up
779	Tppp2	219038	2.72	3.47E-13	up
780	Ccr7	12775	2.72	6.33E-03	up
781	Wbp2nl	74716	2.72	7.30E-25	up
782	Tssk6	83984	2.74	3.74E-17	up
783	Spatc1	74281	2.79	5.51E-22	up
784	Spata32	328019	2.79	5.47E-15	up
785	Pcdhb4	93875	2.82	2.89E-02	up
786	LOC108168667	108168667	2.84	3.44E-03	up
787	H2al3	385317	2.84	1.94E-12	up
788	Cabs1	70977	2.89	3.48E-12	up

789	Spem1	74288	2.92	5.61E-09	up
790	LOC108168552	108168552	2.93	4.35E-04	up
791	Smok3a	545814	2.93	1.36E-15	up
792	Gm28269	100043180	2.93	5.79E-15	up
793	Ubqln5	70980	2.96	6.08E-39	up
794	Gm30083	102631856	2.96	5.64E-23	up
795	Vsig8	240916	2.96	2.47E-02	up
796	Nmur1	14767	2.97	1.44E-04	up
797	Gm5460	432838	2.98	1.49E-13	up
798	4930544D05Rik	668433	3.01	7.59E-11	up
799	H2al1n	385328	3.02	6.15E-12	up
800	Tex44	71863	3.02	6.00E-20	up
801	Lexm	242602	3.04	4.34E-10	up
802	Slc36a3	215332	3.05	1.39E-24	up
803	H2al1k	547154	3.05	1.12E-08	up
804	4933406M09Rik	240755	3.07	1.20E-19	up
805	Plbd1	66857	3.07	8.23E-32	up
806	Smok2b	236574	3.07	1.21E-11	up
807	Tuba8	53857	3.08	3.84E-12	up
808	H2al1m	76383	3.09	2.11E-09	up
809	Pax1	18503	3.09	7.59E-03	up
810	4921530L21Rik	66732	3.10	2.12E-19	up
811	Gm5901	100503879	3.10	1.20E-12	up
812	Fbxo39	628100	3.14	3.11E-22	up
813	Bmp3	110075	3.14	4.85E-05	up
814	Cntn1	12805	3.21	1.83E-02	up
815	1700015G11Rik	100503036	3.21	8.83E-07	up
816	Tmco5b	75275	3.25	1.13E-21	up
817	Pcdha12	192164	3.25	2.10E-03	up
818	Tex37	74221	3.26	1.93E-16	up
819	Trp53tg5	73603	3.27	3.20E-25	up
820	Abhd16b	241850	3.28	5.07E-17	up
821	Ubqlnl	244179	3.36	1.28E-29	up
822	Padi6	242726	3.37	2.11E-04	up
823	1700020A23Rik	75656	3.37	1.11E-10	up
824	Iqcf6	100041096	3.38	6.60E-09	up
825	Tmem239	66766	3.40	1.46E-10	up
826	Prss37	67690	3.46	1.39E-11	up
827	Klk1b8	16624	3.50	1.21E-05	up
828	Cst13	69294	3.53	2.82E-33	up
829	Tnp1	21958	3.56	2.32E-13	up
830	Gm38958	105242869	3.57	4.20E-06	up
831	4930402F06Rik	74854	3.59	1.75E-04	up
832	Zc2hc1b	75122	3.64	6.98E-03	up

833	Odf3b	70113	3.64	3.23E-07	up
834	Fam71b	432552	3.67	1.35E-19	up
835	Gm8674	667507	3.67	2.95E-04	up
836	Spata31d1b	238662	3.71	1.60E-13	up
837	Gm30302	102632152	3.73	1.59E-16	up
838	Mtnr1a	17773	3.76	4.03E-04	up
839	1700042G07Rik	67323	3.78	8.09E-09	up
840	Hemgn	93966	3.80	3.27E-29	up
841	Actrt3	76652	3.82	1.24E-21	up
842	Unc93a2	667055	3.83	1.47E-03	up
843	Ttc24	214191	3.85	7.90E-11	up
844	Tnp2	21959	3.87	5.42E-11	up
845	Gm6468	624049	3.88	4.43E-02	up
846	Prss52	73382	3.89	4.24E-25	up
847	4921517D22Rik	70900	3.92	3.56E-14	up
848	Gm5767	436336	3.95	1.21E-10	up
849	Gm8672	667503	3.95	1.95E-16	up
850	Fscn3	56223	3.99	9.23E-22	up
851	Fam71a	619288	4.00	2.29E-42	up
852	Unc93a	381058	4.02	2.68E-06	up
853	Defb33	654453	4.02	7.15E-07	up
854	Tex46	67663	4.05	2.13E-06	up
855	Cldn13	57255	4.11	1.23E-03	up
856	Gm6637	625931	4.14	1.03E-02	up
857	Oxct2b	353371	4.14	8.15E-30	up
858	Pip5kl1	227733	4.16	4.36E-11	up
859	1700012A03Rik	76382	4.22	6.66E-23	up
860	1700071K01Rik	237880	4.25	2.00E-08	up
861	Grin2b	14812	4.29	9.67E-06	up
862	1700016C15Rik	69428	4.30	5.69E-21	up
863	Spata31d1c	238683	4.31	4.27E-43	up
864	LOC102636989	102636989	4.31	6.74E-15	up
865	Pcdhb1	93872	4.32	3.10E-07	up
866	Glt6d1	71103	4.37	1.35E-19	up
867	Prss51	100504162	4.40	1.69E-10	up
868	Ces3b	13909	4.41	3.07E-02	up
869	Prm1	19118	4.41	6.02E-15	up
870	Gm136	214568	4.43	6.21E-25	up
871	Prm2	19119	4.45	1.60E-13	up
872	Trim42	78911	4.49	2.43E-22	up
873	4931408C20Rik	210940	4.54	5.13E-28	up
874	Gad2	14417	4.58	9.16E-03	up
875	Oxct2a	64059	4.64	1.53E-26	up
876	Gm8777	667712	4.71	1.16E-10	up

877	Bpifa3	73388	4.80	2.32E-14	up
878	Bpifa6	545477	4.82	1.02E-03	up
879	1700080E11Rik	73532	4.89	1.81E-15	up
880	Cyp2a12	13085	4.95	4.20E-03	up
881	1700008P02Rik	69347	5.09	8.13E-11	up
882	Cstl1	228756	5.22	1.60E-10	up
883	4933402N03Rik	233918	5.44	1.58E-05	up
884	LOC108168540	108168540	5.44	6.45E-05	up
885	LOC108168586	108168586	5.45	3.04E-03	up
886	Klk1b9	13648	5.74	1.37E-04	up

Supplementary Table 3. The differentially expressed proteins in *Tmem232*$^{-/-}$ and wild type mice sperms.

No.	Gene Symbol	Protein accession	Fold-change	style	*P*-value
1	Myh7	Q91Z83	0.035	down	3.85E-02
2	Dnah12	Q3V0Q1	0.117	down	2.30E-03
3	Dnah2	P0C6F1	0.123	down	3.37E-02
4	Tcte3	P11985	0.145	down	2.40E-03
5	Tssk4	Q9D411	0.190	down	3.83E-02
6	Ccdc42	Q5SV66	0.190	down	4.60E-02
7	Hspb9	Q9DAM3	0.200	down	1.92E-03
8	Ccdc81	Q9D5W4	0.204	down	1.66E-03
9	Ccdc114	Q3UX62	0.208	down	1.74E-02
10	Mmel1	Q9JLI3	0.215	down	3.40E-03
11	Ccdc39	Q9D5Y1	0.230	down	3.49E-02
12	Armc3	A2AU72	0.242	down	4.22E-03
13	Gm136	Q3V037	0.243	down	1.95E-02
14	Ccdc116	Q80X53	0.246	down	3.42E-02
15	---	Q9DAA7	0.247	down	1.58E-02
16	Kif9	Q9WV04	0.255	down	1.06E-03
17	---	Q3U1D9	0.262	down	3.01E-02
18	Wfdc15a	Q8BH89	0.265	down	4.89E-02
19	Dnah1	E9Q8T7	0.277	down	2.59E-02
20	Tmem89	Q9DA04	0.282	down	2.03E-02
21	Prss54	Q7M756	0.285	down	1.45E-02
22	---	Q9CQT6	0.286	down	5.16E-03
23	Iqcd	Q9D3V1	0.290	down	4.10E-02
24	Defb22	Q8BVC1	0.298	down	8.64E-03
25	Cpa5	Q8R4H4	0.300	down	1.18E-02
26	Ptchd3	Q0EEE2	0.303	down	4.08E-03
27	Zan	O88799	0.305	down	4.28E-03
28	Adam3	F8VQ03	0.307	down	7.66E-03
29	Dusp21	Q9D9D8	0.309	down	2.26E-02
30	Slc25a40	Q8BGP6	0.311	down	1.03E-02
31	Drc3	Q9D5E4	0.314	down	1.48E-03
32	Prss45	Q8K4I7	0.315	down	1.94E-02
33	Izumo4	D3Z690	0.317	down	8.98E-03
34	Lrrc34	Q9DAM1	0.317	down	9.98E-03
35	Tex55	A6X8Z9	0.319	down	3.98E-03
36	Fam205c	Q80YD3	0.321	down	6.42E-03
37	Efhb	Q8CDU5	0.321	down	2.20E-02
38	Lyzl6	Q9DA11	0.321	down	4.29E-02

39	Cfap58	B2RW38	0.322	down	4.66E-03
40	Rsph6a	Q8CDR2	0.322	down	9.24E-03
41	Ccdc183	A2AJB1	0.324	down	6.06E-03
42	Cfap57	Q9D180	0.326	down	4.09E-02
43	Eqtn	Q9D9V2	0.329	down	3.48E-03
44	Enkur	Q6SP97	0.333	down	1.12E-02
45	Ccdc173	A0JLY1	0.335	down	1.24E-02
46	Catsper2	A2ARP9	0.336	down	2.24E-03
47	---	Q4KKZ1	0.340	down	3.72E-03
48	Zpbp	Q62522	0.341	down	5.46E-03
49	Spata6	Q3U6K5	0.341	down	3.83E-02
50	Samd15	F6XZJ7	0.342	down	3.80E-02
51	Gas8	Q60779	0.345	down	1.80E-03
52	Pacrg	Q9DAK2	0.345	down	5.12E-03
53	Cd46	O88174	0.349	down	7.34E-03
54	Ccdc40	Q8BI79	0.351	down	2.08E-03
55	Ppef1	O35655	0.351	down	3.09E-02
56	Zp3r	Q60736	0.354	down	4.74E-03
57	Trim69	Q80X56	0.354	down	1.35E-02
58	Ppp1r32	Q148A4	0.354	down	4.41E-02
59	Spa17	Q62252	0.355	down	2.36E-03
60	Acrbp	Q3V140	0.355	down	3.72E-03
61	Izumo1	Q9D9J7	0.355	down	7.68E-03
62	Ttc25	Q9D4B2	0.356	down	3.94E-03
63	Efcab6	Q6P1E8	0.356	down	5.94E-03
64	Adam24	Q9R160	0.356	down	7.02E-03
65	Nsun4	C4P6S0	0.357	down	5.14E-03
66	Mtfp1	Q9CRB8	0.357	down	9.74E-03
67	Spag6	Q9JLI7	0.358	down	1.69E-02
68	Cyct	P00015	0.359	down	1.30E-02
69	Spag17	Q5S003	0.360	down	3.24E-02
70	Il4i1	O09046	0.361	down	2.42E-03
71	Fscb	A1EGX6	0.361	down	2.58E-02
72	Spata48	Q5NC83	0.362	down	2.40E-02
73	Lrrc23	O35125	0.364	down	8.50E-03
74	Ment	Q569E4	0.365	down	3.98E-03
75	Cfap126	Q6P8X9	0.365	down	4.90E-02
76	Actl7a	Q9QY84	0.366	down	6.22E-03
77	Spaca4	Q80ZQ0	0.367	down	3.48E-03
78	Spaca1	Q9DA48	0.368	down	4.15E-02
79	Bpi	Q67E05	0.371	down	5.38E-03
80	Tepp	Q6IMH0	0.371	down	6.86E-03
81	Zpbp2	Q6X786	0.373	down	5.22E-03
82	Tuba3a	P05214	0.374	down	3.31E-02

83	Gm11492	Q5ND19	0.377	down	7.14E-03
84	Rsph3b	Q9DA80	0.377	down	4.29E-02
85	Hyal5	Q812F3	0.378	down	4.08E-03
86	Acr	P23578	0.378	down	4.54E-03
87	Dnah8	Q91XQ0	0.378	down	8.38E-03
88	Tekt4	Q149S1	0.380	down	2.86E-02
89	Dnajb13	Q80Y75	0.381	down	2.52E-02
90	Pfn3	Q9DAD6	0.382	down	6.16E-03
91	Phospho1	Q8R2H9	0.383	down	6.90E-03
92	Slc16a7	O70451	0.384	down	1.58E-02
93	Cfap53	Q9D439	0.386	down	2.02E-02
94	Ace3	D0G895	0.389	down	2.48E-03
95	Sept4	P28661	0.389	down	2.73E-02
96	Efhc2	Q9D485	0.392	down	2.17E-02
97	Smrp1	Q2MH31	0.393	down	2.71E-02
98	Slc2a5	Q9WV38	0.393	down	4.75E-02
99	Dnai1	Q8C0M8	0.394	down	7.24E-03
100	Dnai2	A2AC93	0.395	down	1.29E-02
101	Insl6	Q9QY05	0.396	down	2.20E-03
102	Dnah17	Q69Z23	0.396	down	9.44E-03
103	Tekt5	G5E8A8	0.397	down	3.61E-02
104	Lcn12	Q6JVL5	0.398	down	2.17E-02
105	Fam166a	Q9D4K5	0.399	down	4.14E-03
106	Ccdc105	Q9D4K7	0.400	down	2.60E-02
107	Pebp4	Q9D9G2	0.401	down	2.24E-03
108	Capza3	P70190	0.402	down	1.30E-02
109	Spaca5	A2AE20	0.403	down	4.14E-03
110	Mns1	Q61884	0.404	down	2.90E-03
111	Ccdc136	Q3TVA9	0.404	down	9.00E-03
112	Satl1	Q9D5N8	0.404	down	1.07E-02
113	Spesp1	Q9D5A0	0.404	down	3.66E-02
114	Odf1	Q61999	0.405	down	1.37E-02
115	Efhc1	Q9D9T8	0.408	down	4.42E-03
116	Ccdc63	Q8CDV6	0.408	down	7.62E-03
117	Oaz3	Q9R109	0.408	down	8.70E-03
118	Cfap52	Q5F201	0.410	down	9.24E-03
119	Odf2	A3KGV1	0.411	down	7.18E-03
120	Ribc2	Q9D4Q1	0.411	down	2.07E-02
121	Man2b2	O54782	0.414	down	7.12E-03
122	Ropn1	Q9ESG2	0.415	down	2.20E-02
123	Mroh2b	Q7M6Y6	0.416	down	4.58E-02
124	Atp8b3	Q6UQ17	0.416	down	4.84E-02
125	Akap3	O88987	0.419	down	6.82E-03
126	Fndc8	Q9D2H8	0.419	down	1.17E-02

127	---	Q9D5Y0	0.420	down	1.98E-02
128	Rsph9	Q9D9V4	0.422	down	3.74E-03
129	Gpx4	O70325	0.422	down	1.47E-02
130	---	Q9DAS2	0.423	down	7.12E-03
131	Lyzl4	Q9D925	0.423	down	7.30E-03
132	Irgc	Q8C262	0.424	down	1.30E-02
133	Ccin	Q8CDE2	0.425	down	8.28E-03
134	Spink2	Q8BMY7	0.426	down	4.44E-02
135	Lelp1	Q9DAE3	0.427	down	1.32E-02
136	Pdha2	P35487	0.427	down	4.28E-02
137	Odf3	Q920N1	0.429	down	9.88E-03
138	Prss52	Q9D9M0	0.434	down	3.84E-02
139	Mpc2	Q9D023	0.439	down	3.04E-03
140	Tekt2	Q922G7	0.443	down	9.90E-03
141	Tssk1b	Q61241	0.443	down	1.09E-02
142	Tex37	Q9DAG4	0.443	down	1.21E-02
143	Tekt1	Q9DAJ2	0.444	down	1.72E-02
144	Fam71b	Q5STT6	0.445	down	7.12E-03
145	Spaca3	Q9D9X8	0.450	down	4.18E-03
146	Tex43	Q9D9I1	0.450	down	2.76E-02
147	Prss39	O70169	0.450	down	3.64E-02
148	Gk2	Q9WU65	0.452	down	1.34E-02
149	Nme8	Q715T0	0.452	down	2.37E-02
150	Nmes1	Q810Q5	0.453	down	3.39E-02
151	Armc4	B2RY50	0.461	down	2.31E-02
152	Cpt1b	Q924X2	0.466	down	4.44E-02
153	Adam1b	Q8R534	0.467	down	1.63E-02
154	Prcp	Q7TMR0	0.468	down	4.28E-03
155	Akap4	Q60662	0.468	down	1.26E-02
156	Spam1	P48794	0.471	down	1.36E-02
157	Thnsl1	Q8BH55	0.473	down	1.20E-02
158	Armc12	Q80X86	0.476	down	3.67E-02
159	Slc2a3	P32037	0.477	down	4.46E-02
160	Adam2	Q60718	0.481	down	1.25E-02
161	Glipr1l2	Q9CQ35	0.483	down	1.14E-02
162	Nme7	Q9QXL8	0.483	down	3.56E-02
163	Arsa	P50428	0.486	down	1.30E-02
164	Hk1	P17710	0.487	down	6.94E-03
165	Plbd1	Q8VCI0	0.488	down	6.10E-03
166	Rsph1	Q8VIG3	0.490	down	3.02E-03
167	Adam5	Q3TTE0	0.492	down	1.28E-02
168	Tmco2	P0C1V4	0.493	down	3.16E-02
169	Cox6b2	Q80ZN9	0.494	down	9.68E-03
170	Hdhd5	Q91WM2	0.495	down	4.02E-02

171	Glipr1l1	Q9DAG6	0.496	down	1.50E-02
172	Slc25a31	Q3V132	0.500	down	9.52E-03
173	Dynlrb2	Q9DAJ5	0.504	down	2.28E-02
174	Cd59b	P58019	0.507	down	5.82E-03
175	Ptpmt1	Q66GT5	0.508	down	1.11E-02
176	Spata18	Q0P557	0.509	down	1.98E-02
177	Camk4	P08414	0.509	down	2.09E-02
178	Ropn1l	Q9EQ00	0.511	down	5.10E-03
179	Dlat	Q8BMF4	0.512	down	1.88E-02
180	---	Q8BPC6	0.512	down	2.65E-02
181	Tmprss12	Q3V0Q7	0.514	down	2.73E-02
182	Sucla2	Q9Z2I9	0.517	down	9.12E-03
183	L2hgdh	Q91YP0	0.517	down	1.97E-02
184	Ak2	Q9WTP6	0.521	down	2.17E-02
185	Crat	P47934	0.526	down	1.27E-02
186	Dnali1	Q8BVN8	0.527	down	1.70E-02
187	Gpd2	Q64521	0.527	down	1.96E-02
188	Gsto2	Q8K2Q2	0.528	down	1.97E-02
189	Sccpdh	Q8R127	0.528	down	2.84E-02
190	Mtch2	Q791V5	0.530	down	2.32E-02
191	Oxsm	Q9D404	0.531	down	7.38E-03
192	Ranbp17	Q99NF8	0.535	down	2.61E-02
193	Rab2a	P53994	0.541	down	2.68E-03
194	Tmem177	Q8BPE4	0.543	down	4.94E-02
195	Glb1l	Q8VC60	0.545	down	2.50E-02
196	Emc7	Q9EP72	0.546	down	3.76E-02
197	Hscb	Q8K3A0	0.548	down	2.98E-02
198	Prss21	Q9JHJ7	0.548	down	3.25E-02
199	Isca1	Q9D924	0.550	down	4.87E-02
200	Fmo3	P97501	0.551	down	1.30E-02
201	Vdac3	Q60931	0.551	down	1.44E-02
202	Fahd2	Q3TC72	0.552	down	1.39E-02
203	Cptp	Q8BS40	0.554	down	4.20E-02
204	Oxct2a	Q9JJN4	0.557	down	1.77E-02
205	Atp1a4	Q9WV27	0.558	down	1.48E-02
206	Nup188	Q6ZQH8	0.565	down	1.61E-02
207	Bspry	Q80YW5	0.566	down	5.48E-03
208	Cuzd1	P70412	0.567	down	1.87E-02
209	Pdhb	Q9D051	0.569	down	2.31E-02
210	Cox5a	P12787	0.570	down	4.71E-02
211	Dnajc11	Q5U458	0.571	down	5.38E-03
212	Dmac2l	Q9CRA7	0.572	down	9.16E-03
213	Spata19	Q9DAQ9	0.572	down	3.82E-02
214	Scamp2	Q9ERN0	0.579	down	3.04E-03

215	Pdzk1	Q9JIL4	0.579	down	4.41E-02
216	Sdhc	Q9CZB0	0.580	down	8.46E-03
217	Tamm41	Q3TUH1	0.580	down	4.59E-02
218	Lypd4	Q8BVP6	0.586	down	3.80E-02
219	Suclg1	Q9WUM5	0.587	down	4.38E-03
220	Mrpl53	Q9D1H8	0.588	down	1.69E-02
221	Defb11	Q8R2I7	0.590	down	2.11E-02
222	Sod2	P09671	0.591	down	7.96E-03
223	C4bpa	P08607	0.591	down	1.26E-02
224	Mpc1	P63030	0.592	down	4.72E-02
225	Cox5b	P19536	0.597	down	5.74E-03
226	Cisd1	Q91WS0	0.597	down	9.76E-03
227	Cox6a1	P43024	0.598	down	2.05E-02
228	Htra2	Q9JIY5	0.603	down	6.25E-05
229	Mtco1	P00397	0.604	down	5.16E-04
230	Enpp2	Q9R1E6	0.604	down	3.61E-02
231	Csnk2a2	O54833	0.608	down	1.15E-02
232	Atp2b4	Q6Q477	0.614	down	7.24E-03
233	Vdac2	Q60930	0.615	down	6.38E-03
234	Csnk1g2	Q8BVP5	0.624	down	2.37E-02
235	Dld	O08749	0.625	down	1.92E-02
236	Cox4i1	P19783	0.626	down	2.64E-03
237	Uqcrc2	Q9DB77	0.629	down	9.10E-03
238	Cs	Q9CZU6	0.635	down	9.36E-03
239	Acot9	Q9R0X4	0.636	down	2.94E-03
240	Atp6v0a4	Q920R6	0.636	down	9.30E-03
241	Lipg	Q9WVG5	0.637	down	2.64E-02
242	Cpt2	P52825	0.638	down	4.86E-03
243	Mycbp	Q9EQS3	0.638	down	2.43E-02
244	Ndufb7	Q9CR61	0.640	down	3.64E-03
245	Prkaca	P05132	0.642	down	2.35E-02
246	Wfdc15b	Q9JHY4	0.643	down	3.56E-02
247	Ogdh	Q60597	0.648	down	5.06E-03
248	Sdha	Q8K2B3	0.649	down	8.14E-03
249	Chchd6	Q91VN4	0.649	down	1.58E-02
250	Spink10	Q8CAC8	0.653	down	2.15E-02
251	Ndufab1	Q9CR21	0.658	down	8.78E-03
252	Angel2	Q8K1C0	0.660	down	2.60E-02
253	Abhd11	Q8K4F5	0.662	down	3.39E-02
254	Ckmt1	P30275	0.665	down	7.00E-03
255	Gsk3a	Q2NL51	0.666	down	2.33E-02
256	Naa15	Q80UM3	1.504	up	1.47E-02
257	Tmf1	B9EKI3	1.504	up	4.16E-02
258	Pcsk1n	Q9QXV0	1.506	up	2.32E-02

259	Ctsz	Q9WUU7	1.512	up	8.16E-03
260	Rpl18a	P62717	1.514	up	2.77E-02
261	Txnrd3	Q99MD6	1.517	up	1.50E-02
262	Rps6	P62754	1.520	up	2.42E-03
263	Eif4a2	P10630	1.522	up	1.71E-02
264	Rpl10	Q6ZWV3	1.532	up	1.89E-02
265	Sorbs3	Q9R1Z8	1.536	up	1.74E-03
266	Aimp2	Q8R010	1.536	up	7.66E-03
267	Pdlim2	Q8R1G6	1.536	up	3.39E-02
268	Cdc42ep4	Q9JM96	1.545	up	4.55E-02
269	Pa2g4	P50580	1.554	up	2.49E-02
270	Rpl7a	P12970	1.558	up	6.62E-03
271	Rps23	P62267	1.560	up	4.21E-02
272	Pyroxd2	Q3U4I7	1.564	up	3.37E-02
273	Smap	Q9R0P4	1.564	up	3.38E-02
274	Rpl38	Q9JJI8	1.564	up	4.38E-02
275	Rps8	P62242	1.582	up	7.63E-04
276	Znf512	Q69Z99	1.582	up	3.13E-02
277	Fuom	Q8R2K1	1.593	up	4.16E-02
278	Gdpd1	Q9CRY7	1.598	up	2.69E-02
279	Ubxn7	Q6P5G6	1.602	up	2.33E-02
280	Hcls1	P49710	1.605	up	3.18E-03
281	Myd88	P22366	1.618	up	3.78E-02
282	Tti1	Q91V83	1.622	up	1.13E-02
283	H2-Aa	P14438	1.622	up	2.98E-02
284	Mbnl2	Q8C181	1.625	up	1.94E-02
285	Impad1	Q80V26	1.627	up	4.81E-02
286	Farsa	Q8C0C7	1.628	up	9.36E-03
287	Nup88	Q8CEC0	1.631	up	2.03E-02
288	Ifi35	Q9D8C4	1.637	up	3.10E-02
289	Kars	Q99MN1	1.638	up	1.44E-03
290	Iars	Q8BU30	1.671	up	6.70E-03
291	Ubap2l	Q80X50	1.687	up	1.22E-02
292	Rps24	P62849	1.692	up	8.03E-04
293	Aimp1	P31230	1.700	up	6.96E-03
294	Mcm6	P97311	1.716	up	7.28E-03
295	Creld2	Q9CYA0	1.729	up	1.50E-03
296	Itih3	Q61704	1.752	up	1.15E-02
297	Irgq	Q8VIM9	1.756	up	3.27E-02
298	Eef1d	P57776	1.760	up	5.39E-04
299	Fut11	Q8BHC9	1.772	up	1.32E-02
300	Hist1h4a	P62806	1.776	up	4.68E-02
301	Cherp	Q8CGZ0	1.799	up	3.61E-04
302	Vars	Q9Z1Q9	1.810	up	3.86E-03

303	Rpl21	O09167	1.817	up	1.89E-02
304	Arih2	Q9Z1K6	1.821	up	4.25E-02
305	Rpl15	Q9CZM2	1.856	up	4.38E-03
306	Nelfe	P19426	1.878	up	3.32E-02
307	Arfgap3	Q9D8S3	1.878	up	4.35E-02
308	Rps25	P62852	1.889	up	2.45E-02
309	Pex14	Q9R0A0	1.904	up	1.26E-03
310	Cars	Q9ER72	1.920	up	2.04E-04
311	Btf3l4	Q9CQH7	1.931	up	3.10E-02
312	Naa25	Q8BWZ3	1.935	up	1.62E-03
313	Rpl17	Q9CPR4	1.985	up	5.38E-03
314	Cadm1	Q8R5M8	1.993	up	3.00E-02
315	Rpl4	Q9D8E6	1.996	up	1.36E-02
316	Czib	Q8BHG2	2.010	up	4.38E-03
317	Abcb6	Q9DC29	2.053	up	1.77E-02
318	Rps28	P62858	2.111	up	1.77E-02
319	Spert	Q32MG2	2.118	up	1.85E-02
320	Tarbp2	P97473	2.168	up	6.22E-03
321	H3f3a	P84244	2.168	up	1.81E-02
322	Rpl23	P62830	2.178	up	1.03E-02
323	Dazap1	Q9JII5	2.200	up	1.70E-03
324	Rpl14	Q9CR57	2.205	up	2.63E-02
325	Atxn2	O70305	2.208	up	6.48E-03
326	Rpl3	P27659	2.222	up	1.52E-02
327	Rpl32	P62911	2.244	up	2.24E-06
328	Galnt11	Q921L8	2.412	up	2.74E-02
329	Rpl13	P47963	2.462	up	1.40E-03
330	Mfge8	P21956	2.466	up	2.41E-02
331	Rpl36	P47964	2.515	up	1.20E-03
332	Hist1h1e	P43274	2.519	up	4.91E-02
333	Ociad2	Q9D8W7	2.612	up	2.94E-03
334	Rpl6	P47911	2.672	up	1.56E-02
335	Ero1b	Q8R2E9	2.932	up	4.06E-03
336	Eef1g	Q9D8N0	3.515	up	7.56E-04
337	Clu	Q06890	3.542	up	1.97E-04
338	Rpl27a	P14115	3.623	up	1.10E-02
339	Clca3a1	Q9QX15	3.950	up	4.04E-04
340	Crisp2	P16563	4.132	up	2.04E-03
341	Pdilt	Q9DAN1	5.054	up	9.21E-04
342	Plau	P06869	5.349	up	1.06E-02
343	Mmp7	Q10738	5.350	up	4.24E-03

Supplementary Table 4. The significant GO terms of differentially expressed proteins between *Tmem232*$^{-/-}$ and wild type mice sperms.

Ontology	Term	ID	Input number	*P*-Value	Input genes
Cellular Component	sperm part	GO:0097223	60	4.9029E-44	Ak2, Spaca1,Dnah1, Spesp1, Atp8b3, Prss39, Tekt5, Dnah8, Prkaca, Ace3, Rsph1, Odf2, Gas8, Odf1, Dld, Nme8, Ropn1, Tekt4, Enkur, Spag6, Spaca3, Spink2, Hyal5, Tekt1, Dnai2, Tssk4, 44808, Arsa, Spata6, Hk1, Spam1, Ccdc136, Tcte3, Csnk2a2, Acr, Fscb, Cd46, Dnajb13, Zpbp, Akap4, Catsper2, Eqtn, Pacrg, Acrbp, Zp3r, Atp2b4, Tssk1b, Slc2a3, Zpbp2, Izumo1, Mroh2b, Glipr1l1, Spaca4, Tekt2, Odf3, Akap3, Ptchd3, Mns1, Spa17, Spink10
Cellular Component	motile cilium	GO:0031514	43	1.6645E-31	Ak2, Dnah1, Tekt5, Dnah8, Spag17, Prkaca, Rsph1, Odf2, Gas8, Odf1, Dld, Nme8, Ropn1, Tekt4, Enkur, Spag6, Tekt1, Actl7a, Dnai2, Tssk4, 44808, Spata6, Hk1, Ropn1l, Tcte3, Slc25a31, Fscb, Dnajb13, Akap4, Catsper2, Pacrg, Atp2b4, Tssk1b, Mroh2b, Glipr1l1, Tekt2, Rsph9, Odf3, Akap3, Ptchd3, Mns1, Spa17, Dnah2
Cellular Component	sperm flagellum	GO:0036126	34	2.12465E-24	Ak2, Dnah1, Tekt5, Dnah8, Prkaca, Rsph1, Odf2, Gas8, Odf1, Nme8, Ropn1, Tekt4, Enkur, Spag6, Tekt1, Dnai2, 44808, Spata6, Hk1, Tcte3, Fscb, Dnajb13, Akap4, Catsper2, Pacrg, Atp2b4, Mroh2b, Glipr1l1, Tekt2, Odf3, Akap3, Ptchd3, Spa17, Mns1
Cellular Component	9+2 motile cilium	GO:0097729	34	2.12465E-24	Ak2, Dnah1, Tekt5, Dnah8, Prkaca, Rsph1, Odf2, Gas8, Odf1, Nme8, Ropn1, Tekt4, Enkur, Spag6, Tekt1, Dnai2, 44808, Spata6, Hk1, Tcte3, Fscb, Dnajb13, Akap4, Catsper2, Pacrg, Atp2b4, Mroh2b, Glipr1l1, Tekt2, Odf3, Akap3, Ptchd3, Spa17, Mns1

Cellular Component	acrosomal vesicle	GO:0001669	31	1.3319E-23	Csnk2a2, Acr, Spaca1, Spesp1, Atp8b3, Prss39, Cd46, Zpbp, Eqtn, Prkaca, Ace3, Acrbp, Zp3r, Tssk1b, Dld, Slc2a3, Zpbp2, Izumo1, Enkur, Mroh2b, Glipr1l1, Spaca3, Spink2, Spaca4, Hyal5, Tssk4, Arsa, Akap3, Ccdc136, Spam1, Spink10
Cellular Component	ciliary plasm	GO:0097014	23	2.23155E-14	Dnah1, Armc4, Dnajb13, Dnah8, Spag17, Gas8, Drc3, Nme8, Ccdc63, Spag6, Dnai1, Dnali1, Efhc1, Dnai2, Dnah17, Rsph9, Ccdc39, Ccdc114, Dnah12, Mns1, Dnah2, Ccdc183, Ccdc40
Cellular Component	axoneme	GO:0005930	23	2.23155E-14	Dnah1, Armc4, Dnajb13, Dnah8, Spag17, Gas8, Drc3, Nme8, Ccdc63, Spag6, Dnai1, Dnali1, Efhc1, Dnai2, Dnah17, Rsph9, Ccdc39, Ccdc114, Dnah12, Mns1, Dnah2, Ccdc183, Ccdc40
Cellular Component	ciliary part	GO:0044441	47	1.39888E-13	Ak2, Dnah1, Armc4, Dnah8, Spag17, Prkaca, Rsph1, Odf2, Gas8, Odf1, Nme8, Ccdc63, Ropn1, Tekt4, Enkur, Spag6, Efhc1, Dnai2, Dnah17, 44808, Spata6, Hk1, Ccdc39, Ccdc114, Dnah12, Nme7, Tuba3a, Ccdc183, Fscb, Dnajb13, Akap4, Pacrg, Drc3, Atp2b4, Mroh2b, Dnai1, Glipr1l1, Dnali1, Cfap126, Rsph9, Odf3, Akap3, Ptchd3, Mns1, Spa17, Dnah2, Ccdc40
Molecular Function	structural constituent of ribosome	GO:0003735	20	9.29523E-06	Rpl32, Rpl13, Rps8, Rpl6, Rps24, Rpl4, Rpl17, Rpl27a, Rps28, Rps6, Rpl3, Rpl21, Rps23, Rpl23, Rpl38, Rpl14, Rpl36, Rpl15, Rpl18a, Rpl10
Molecular Function	microtubule motor activity	GO:0003777	7	0.000166077	Dnah1, Dnah12, Kif9, Dnah8, Dnai2, Dnah2, Dnah17
Molecular Function	electron carrier activity	GO:0009055	10	0.000166188	Cox4i1, Cyct, Cox5a, Cox6a1, Txnrd3, Sdhc, Mtco1, Cox6b2, Cox5b, Sdha
Molecular Function	peptidoglycan muralytic activity	GO:0061783	4	0.000233096	Spaca5, Spaca3, Lyzl6, Lyzl4
Molecular	pyruvate dehydrogenase	GO:0004738	4	0.000233096	Pdhb, Pdha2, Dlat, Dld

Function	activity				
Molecular Function	lysozyme activity	GO:0003796	4	0.000233096	Spaca5, Spaca3, Lyzl6, Lyzl4
Molecular Function	motor activity	GO:0003774	12	0.000263127	Tcte3, Dnah1, Dnai1, Dnali1, Dnah8, Dnai2, Dnah17, Dnah12, Kif9, Myh7, Dynlrb2, Dnah2
Molecular Function	metalloendopeptidase activity	GO:0004222	8	0.000365073	Mmel1, Adam1b, Mmp7, Adam5, Uqcrc2, Adam24, Adam2, Adam3
Biological Process	cilium movement	GO:0003341	20	5.47954E-17	Dnah1, Tekt5, Armc4, Spag17, Gas8, Ccdc63, Tekt4, Dnai1, Dnali1, Tekt1, Dnai2, Tekt2, Rsph9, Ccdc114, Cfap53, Ccdc39, Ropn1l, Nme7, Ccdc40, Ccdc183
Biological Process	fertilization	GO:0009566	28	6.6317E-17	Acr, Spesp1, Atp8b3, Cd46, Zpbp, Adam24, H3f3a, Catsper2, Eqtn, Insl6, Adam3, Zp3r, Zan, Zpbp2, Izumo1, Atp1a4, Tarbp2, Glipr1l1, Spaca3, Spink2, Hyal5, Mfge8, Arsa, Spa17, Adam2, Spam1, Ccdc136, Ubap2l
Biological Process	male gamete generation	GO:0048232	48	2.35034E-13	Spaca1, Spata18, Adam24, Capza3, Insl6, Prkaca, Rsph1, Txnrd3, Odf2, Adam1b, Odf1, Dld, Nme8, Ccdc63, Ropn1, Cadm1, Prss21, Spag6, Tarbp2, Oaz3, Spink2, Tssk4, 44808, Spata6, Ropn1l, Ccdc136, Ccin, Csnk2a2, Spata19, H3f3a, Zpbp, Catsper2, Eqtn, Pacrg, Ccdc42, Acrbp, Pdilt, Tmf1, Dazap1, Atp2b4, Tssk1b, Smrp1, Gpx4, Zpbp2, Mroh2b, Atp1a4, Odf3, Mns1
Biological Process	gamete generation	GO:0007276	49	2.49689E-13	Spaca1, Spata18, Adam24, Capza3, Insl6, Prkaca, Rsph1, Txnrd3, Odf2, Adam1b, Odf1, Dld, Nme8, Ccdc63, Ropn1, Cadm1, Prss21, Spag6, Tarbp2, Oaz3, Spink2, Tssk4, 44808, Spata6, Ropn1l, Ccdc136, Ccin, Csnk2a2, Spata19, H3f3a, Zpbp, Catsper2, Eqtn, Pacrg, Ccdc42, Acrbp, Pdilt, Tmf1, Dazap1, Atp2b4, Tssk1b, Smrp1, Gpx4, Zpbp2,

					Rps6, Mroh2b, Atp1a4, Odf3, Mns1
Biological Process	single fertilization	GO:0007338	24	2.92433E-13	Acr, Spesp1, Atp8b3, Cd46, Zpbp, Adam24, H3f3a, Eqtn, Adam3, Zp3r, Zan, Zpbp2, Izumo1, Tarbp2, Glipr1l1, Spaca3, Hyal5, Mfge8, Arsa, Spa17, Spam1, Ccdc136, Adam2, Ubap2l
Biological Process	sperm motility	GO:0097722	19	4.40536E-13	Dnah1, Tekt5, Akap4, Catsper2, Insl6, Gas8, Tmf1, Atp2b4, Nme8, Ropn1, Tekt4, Atp1a4, Spag6, Dnai1, Tekt1, Tekt2, Ccdc39, Ropn1l, Ccdc40
Biological Process	flagellated sperm motility	GO:0030317	19	4.40536E-13	Dnah1, Tekt5, Akap4, Catsper2, Insl6, Gas8, Tmf1, Atp2b4, Nme8, Ropn1, Tekt4, Atp1a4, Spag6, Dnai1, Tekt1, Tekt2, Ccdc39, Ropn1l, Ccdc40
Biological Process	cilium organization	GO:0044782	28	4.51972E-13	Dnah1, Tekt5, Armc4, Dnajb13, Spag17, Akap4, Rsph1, Gas8, Nme8, Ccdc63, Ropn1, Tekt4, Spag6, Dnai1, Tekt1, Cfap126, Dnai2, Tekt2, Rsph9, Galnt11, Spata6, Ccdc39, Cfap53, Ccdc114, Mns1, Nme7, Ccdc183, Ccdc40
Biological Process	germ cell development	GO:0007281	27	1.79157E-12	Spaca1, Zpbp, H3f3a, Capza3, Catsper2, Eqtn, Insl6, Pacrg, Prkaca, Rsph1, Ccdc42, Acrbp, Odf2, Tmf1, Pdilt, Tssk1b, Dld, Ccdc63, Zpbp2, Rps6, Ropn1, Spag6, Tarbp2, Spink2, 44808, Ropn1l, Ccdc136
Biological Process	cilium assembly	GO:0060271	24	7.35609E-11	Dnah1, Tekt5, Armc4, Dnajb13, Spag17, Akap4, Rsph1, Gas8, Nme8, Ccdc63, Tekt4, Spag6, Dnai1, Tekt1, Dnai2, Tekt2, Rsph9, Galnt11, Spata6, Ccdc39, Ccdc114, Cfap53, Ccdc183, Ccdc40
Biological Process	microtubule bundle formation	GO:0001578	16	1.06851E-10	Ccdc63, Dnah1, Spag6, Dnai1, Armc4, Dnajb13, Spag17, Dnai2, Tekt2, Rsph9, Ccdc39, Rsph1, Ccdc114, Gas8, Ccdc40, Ccdc183
Biological Process	cilium or flagellum-dependent cell motility	GO:0001539	8	1.2397E-07	Tekt4, Dnah1, Tekt5, Tekt1, Rsph9, Tekt2, Ccdc39, Gas8

Biological Process	cilium-dependent cell motility	GO:0060285	8	1.2397E-07	Tekt4, Dnah1, Tekt5, Tekt1, Rsph9, Tekt2, Ccdc39, Gas8
Biological Process	epithelial cilium movement	GO:0003351	8	1.2397E-07	Dnah1, Dnai1, Spag17, Cfap53, Ccdc39, Gas8, Nme7, Ccdc40

Supplementary Table 5. PCR primers for quantitative real-time PCR.

No.	Primer name	Sequence (5'-3')	Amplicon (bp)	Annealing temperature (℃)
1	Septin2-FP	AGAAACTCCTGGCTATGTTGGA	214	60
	Septin2-RP	AGCCTCTATCTGGACAGTTCTTT		
2	Septin4-FP	TGAGCTGAGCAAGTTCGTGAA	152	60
	Septin4-RP	ACAAGGAGCCTCTAAACTCCAC		
3	Septin5-FP	GCAGTACGTTGGCTTCGCC	132	60
	Septin5-RP	AGAAAGAGGCTATGGACAAGGG		
4	Septin6-FP	TGTAGGCTTTGGGGATCAGAT	132	60
	Septin6-RP	GAGTCATGGTAGGAGTGCAGT		
5	Septin7-FP	AACAGGGTGCAGTGTTGTTTA	146	60
	Septin7-RP	CATTCCTCTGGTGTAAGTGTGTC		
6	Septin8-FP	CAGTCAAGTCAGTCACTCAGGG	108	60
	Septin8-RP	GTCTCAAAGGTCGTGTTGAAGA		
7	Septin10-FP	GCCTCATGCGACGAGATAAAA	103	60
	Septin10-RP	GCTCAATGGATCTATCGACCAG		
8	Septin11-FP	GTGGGGAGACCGAGTAATGAA	102	60
	Septin11-RP	TTGAGAAGTGGATTTGTTGACCA		
9	ATAT1-FP	CAACTGGCCATTGACCGAC	116	60
	ATAT1-RP	GCAAAGAAGCCTTCAAAGATGA		

Supplementary Table 6. PCR primers and vectors for plasmid construction.

No.	Primer name	Sequence (5'-3')	Amplicon (bp)	Vector	Annealing temperature (℃)
1	TMEM232-FP	TACAAGGATGACGATGACAAG CTTATGAATATGCCTGTTAAC	1974	*p3×FLAG-Myc-CMV-24*	50
	TMEM232-RP	TGTTCGGATCCTCTAGAGTCGA CTTAAATTACTT CCTTCCTATA			
2	TMEM232-FP	GCGCTACCGGACTCAGATCTCG AGATGAATATGC CTGTTAAC	1974	*GFP-N2*	50
	TMEM232-RP	GGTACCGTCGACTGCAGAATTC AATTACTTCCTTCCTAT			
3	TMEM232-FP	AGGGGCCCCTGGGATCCCCGG AATTCATGAATATGCCTGTTAA	2026	*pGEX-6P-1*	45
	TMEM232-RP	AGTCAGTCACGATGCGGCCGCT CGAGTTAAATTACTTCCTTCCT			
4	SEPTIN2-FP	GACAAGCTTGCGGCCGCGAAT TCAATGTCTAAGC AACAGCCA	1086	*p3×FLAG-Myc-CMV-24*	58
	SEPTIN2-RP	TGTTCGGATCCTCTAGAGTCGA CTTACACGTG GTGCCCGAGA			
5	SEPTIN2-FP	CGCTACCGGACTCAGATCTCGA GATGTCTAAGCA ACAGCCA	1086	*GFP-N2*	55
	SEPTIN2-RP	GGTACCGTCGACTGCAGAATTC CACGTGGTGC CCGAG			
6	SEPTIN4-FP	AAGCTTGCGGCCGCGAATTCA ATGGACCGTTCAC TG	1187	*p3×FLAG-Myc-CMV-24*	53
	SEPTIN4-RP	TTGTTCGGATCCTCTAGAGTCG ACTTAATAGT TCTCCTTCAT			
7	SEPTIN4-FP	CGCTACCGGACTCAGATCTCGA GATGGACCGTTCACTGGGA	1187	*GFP-N2*	52
	SEPTIN4-RP	GCGGTACCGTCGACTGCAGAA TTCATAGTTCTCCTTCATCTG			
8	SEPTIN6-FP	CAAGCTTGCGGCCGCGAATTCA ATGGCAGCGACC GATA	1284	*p3×FLAG-Myc-CMV-24*	50
	SEPTIN6-RP	TTGTTCGGATCCTCTAGAGTCG ACTTAATTTTTCTTCTCTTT			
9	SEPTIN6-FP	GCTACCGGACTCAGATCTCGAG ATGGCAGCGACCGATATA	1284	*GFP-N2*	52
	SEPTIN6-RP	CGGTACCGTCGACTGCAGAATT CATTTTTCTT CTCTTTGTC			
10	SEPTIN7-FP	CAAGCTTGCGGCCGCGAATTCA ATGTCGGTCAGTGCGAGA	1342	*p3×FLAG-Myc-CMV-24*	55

	SEPTIN7-RP	GTTCGGATCCTCTAGAGTCGAC TTAAAAGATCTTCCCTTT			
11	SEPTIN7-FP	GCTACCGGACTCAGATCTCGAG ATGTCGGTCAGTGCGAGA	1342	*GFP-N2*	55
	SEPTIN7-RP	CGGTACCGTCGACTGCAGAATT CAAAGATCTTCCCTTTCTT			
12	SEPTIN11-FP	CAAGCTTGCGGCCGCGAATTCA ATGGAGGAGAGGAAACCA	1320	*p3×FLAG-Myc-CMV-24*	52
	SEPTIN11-RP	GTTCGGATCCTCTAGAGTCGAC TTATGTGAAGCTTGCATT			
13	SEPTIN11-FP	CGCTACCGGACTCAGATCTCGA GATGGAGGAGAGGAAACCA	1320	*GFP-N2*	54
	SEPTIN11-RP	GCGGTACCGTCGACTGCAGAAT TCTGTGAAGCTTGCATTTTT			
14	SEPTIN12-FP	CAAGCTTGCGGCCGCGAATTCA ATGGACCCCCTGAGG	1077	*p3×FLAG-Myc-CMV-24*	50
	SEPTIN12-RP	TTGTTCGGATCCTCTAGAGTCG ACTCAGAACTCATCATCAGA			
15	SEPTIN12-FP	GCTACCGGACTCAGATCTCGAG ATGGACCCCCTGAGGCGC	1077	*GFP-N2*	51
	SEPTIN12-RP	CGGTACCGTCGACTGCAGAATT CGAACTCATCATCAGAATC			
16	SEPTIN14-FP	GACAAGCTTGCGGCCGCGAAT TCAATGGCAGAAAGAACAATG	1299	*p3×FLAG-Myc-CMV-24*	50
	SEPTIN14-RP	GTTCGGATCCTCTAGAGTCGAC TTATTTCTTACGATGTTT			
17	SEPTIN14-FP	GCTACCGGACTCAGATCTCGAG ATGGCAGAAAGAACAATG	1299	*GFP-N2*	50
	SEPTIN14-RP	CGGTACCGTCGACTGCAGAATT CTTTCTTACGATGTTTGTC			
18	TMEM232(1-168)-FP	GCTACCGGACTCAGATCTCGAG ATGAATATGCCTGTTAACAA	504	*GFP-N2*	50
	TMEM232(1-168)-RP	GGTACCGTCGACTGCAGAATTC AATCTTTGCTAGCTTTATTT			
19	TMEM232(1-352)-FP	GCTACCGGACTCAGATCTCGAG ATGAATATGCCTGTTAACAA	1056	*GFP-N2*	50
	TMEM232(1-352)-RP	GTACCGTCGACTGCAG AATTCGGAAAAATCAT CTACCTTGC			
20	TMEM232(168-657)-FP	ACCGGACTCAGATCTCG AGATGATTGGCTATTTG GTCTTCTT	1473	*GFP-N2*	53
	TMEM232(16	CGGTACCGTCGACTGCAGAATT			

	8-657)-RP	CAATTACTTCCTTCCTATAAG			
21	TMEM232(189-352)-FP	CTACCGGACTCAGATCTCGAGATGAAACAACATTTACTTAGGCT	495	*GFP-N2*	54
	TMEM232(189-352)-RP	GGTACCGTCGACTGCAGAATTCGGAAAAATCATCTACCTTGC			
22	TMEM232(374-657)-FP	CTACCGGACTCAGATCTCGAGATGACTTCTGATTTGCGAAAAAC	855	*GFP-N2*	52
	TMEM232(374-657)-RP	GCGGTACCGTCGACTGCAGAATTCAATTACTTCCTTCCTATAAG			
23	ARMC12-FP	AAGGATGACGATGACAAGCTTATGGGCAAGAGCATC	1023	*p3×FLAG-Myc-CMV-24*	52
	ARMC12-RP	TTCGGATCCTCTAGAGTCGACTTATTCCGTGTTTTTAAAGTAGGAAC			
24	ARMC12-FP	GTCCCGGACTATGCAGGATCCATGGGCAAGAGCATC	1023	*pcDNA3-3×HA-tag*	52
	ARMC12-RP	AACAGATCTATCGATCTCGAGTTATTCCGTGTTTTTAAAGTAGGAAC			
25	VDAC3-FP	AAGGATGACGATGACAAGCTTATGTGTAACACACCAACGTAC	852	*p3×FLAG-Myc-CMV-24*	55
	VDAC3-RP	TTCGGATCCTCTAGAGTCGACTTAAGCTTCCAGTTCAAATCCC			
26	VDAC3-FP	GTCCCGGACTATGCAGGATCCATGTGTAACACACCAACGTAC	852	*pcDNA3-3×HA-tag*	55
	VDAC3-RP	AACAGATCTATCGATCTCGAGTTAAGCTTCCAGTTCAAATCCC			
27	ATG14-FP	CTTCGAATTCTGCAGTCGACATGGCGTCTCCCAGTGGGAA	1521	*GFP-N2*	60
	ATG14-RP	ACCATGGTGGCGATGGATCCACGGTGTCCAGTGTAAGCTTTAAACC			
