# Supplementary material for: TMEM232 is required for the formation of sperm flagellum and male fertility in mice
Source: Cell Death Dis. 2024 Nov 8;15(11):806. doi: 10.1038/s41419-024-07200-9 (PMC11549365; doi:10.1038/s41419-024-07200-9)
Supplement: Supplementary file 2 — Supplementary Figure [file 41419_2024_7200_MOESM2_ESM.pdf]

Supplementary Figure legends

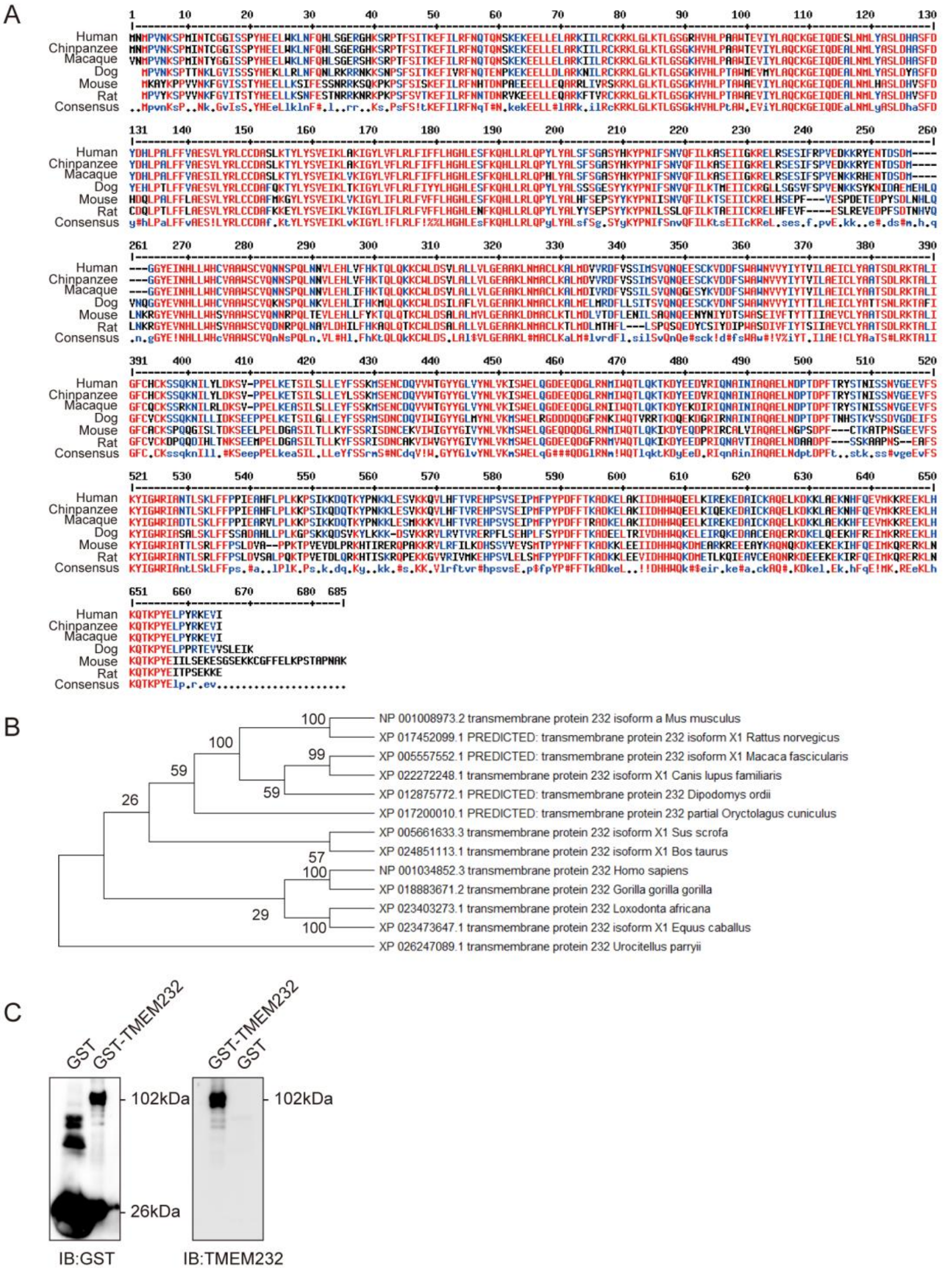

Figure S1. Conservative analysis of TMEM232 and verification of customized anti-Tmem232. (A) The high homology of the TMEM232 protein sequence in several species. Residues that are identical between human and chimpanzee, macaque, dog, mouse or rat TMEM232 are marked in red and as uppercase letters in the consensus line. Residues highly similar between human and other species TMEM232 are indicated by red symbols (! is any one of I and V, $ is any one of L and M, % is any one of F and Y, # is any one of N, D, Q, E, B, and Z). Unconserved residues appear in blue or as asterisks in the consensus line. The alignment was performed using the

online software MultAlin (http://multalin.toulouse.inra.fr/multalin/multalin.html). (B) Evolutionary constraint of the TMEM232. The TMEM232 protein is present in all sequenced vertebrates and is conserved in mammals. The data were retrieved from the Ensemble browser (http://asia.ensembl.org/index.html). (C) The eukaryotic expression vectors of TMEM232 with GST label were constructed, and the recombinant proteins of TMEM232 were obtained by the overexpression in Escherichia coli. TMEM232 recombinant protein was analyzed by Western blot respectively with anti-GST and anti-Tmem232 to verify the specificity of the antiserum.

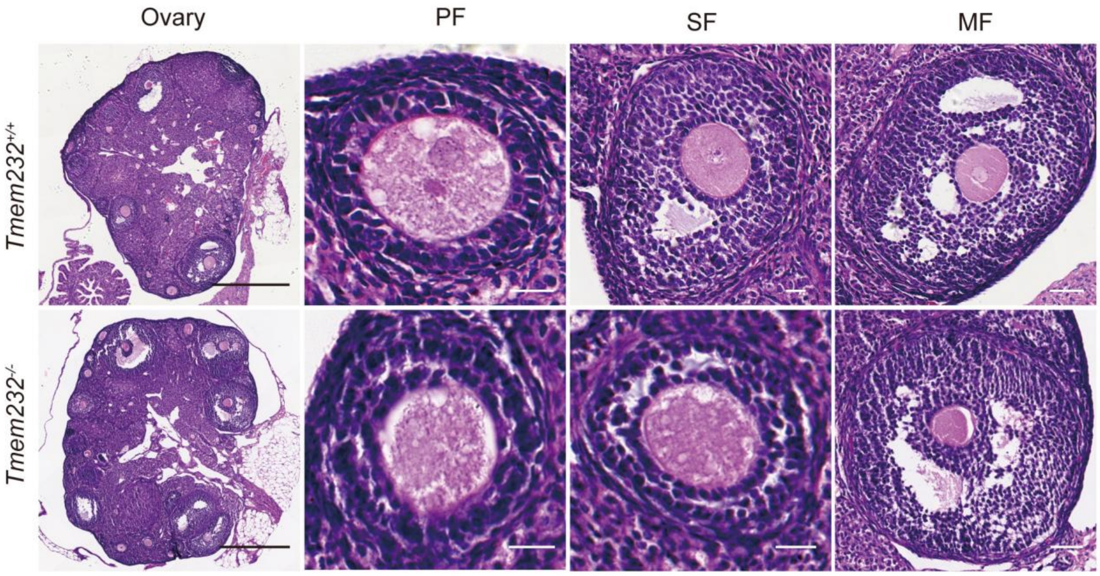

Figure S2. The hematoxylin and eosin (H&E) staining of ovary tissue sections of *Tmem232*$^{+/+}$ and *Tmem232*$^{-/-}$ mouse. PF: primary follicles, SF: secondary follicles, and MF: mature follicles. Scale bar: 500 µm (1st column) or 20 µm (2nd - 4th columns) .

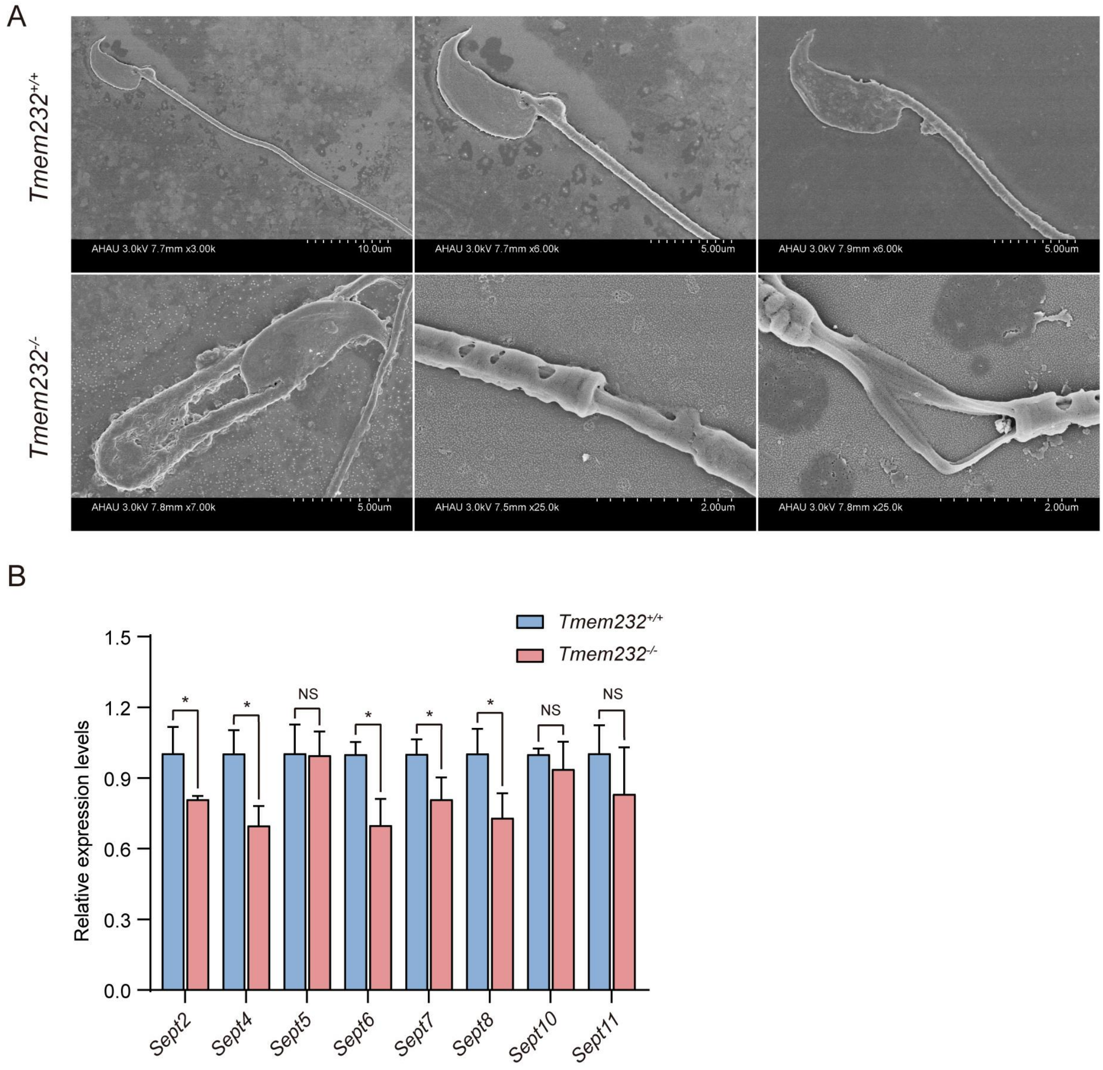

Figure S3. Morphological defects of the sperm flagella in *Tmem232*$^{-/-}$ mice. (A) Longitudinal section of a scanning electron micro-graph showing the abnormal sperm flagella of *Tmem232*$^{-/-}$ mice. Scale bar: 2 μm, 5 μm or 10 μm. (B) Quantitative real-time PCR (qPCR) showed that the septin-based structure composed of septin family members 2, 4, 5, 6, 7, 8, 10 and 11 mRNA expression level in testes of *Tmem232*$^{-/-}$ and wild-type mouse. *indicates $P < 0.05$. NS indicates no significant (Student's t test). n=3.

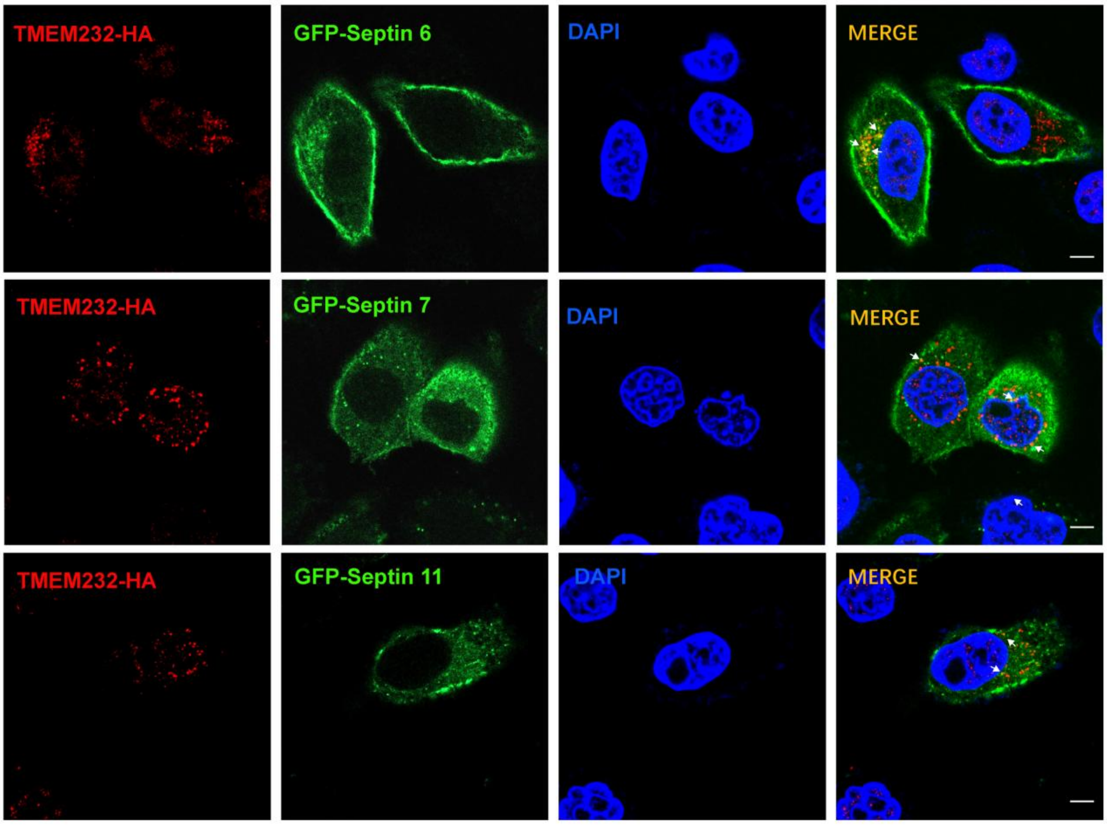

Figure S4. Subcellular localization of intracellular TMEM232 and Septins. Arrowhead indicated the co-localization region of TMEM232 and septins. Scale bar: 10 μm.

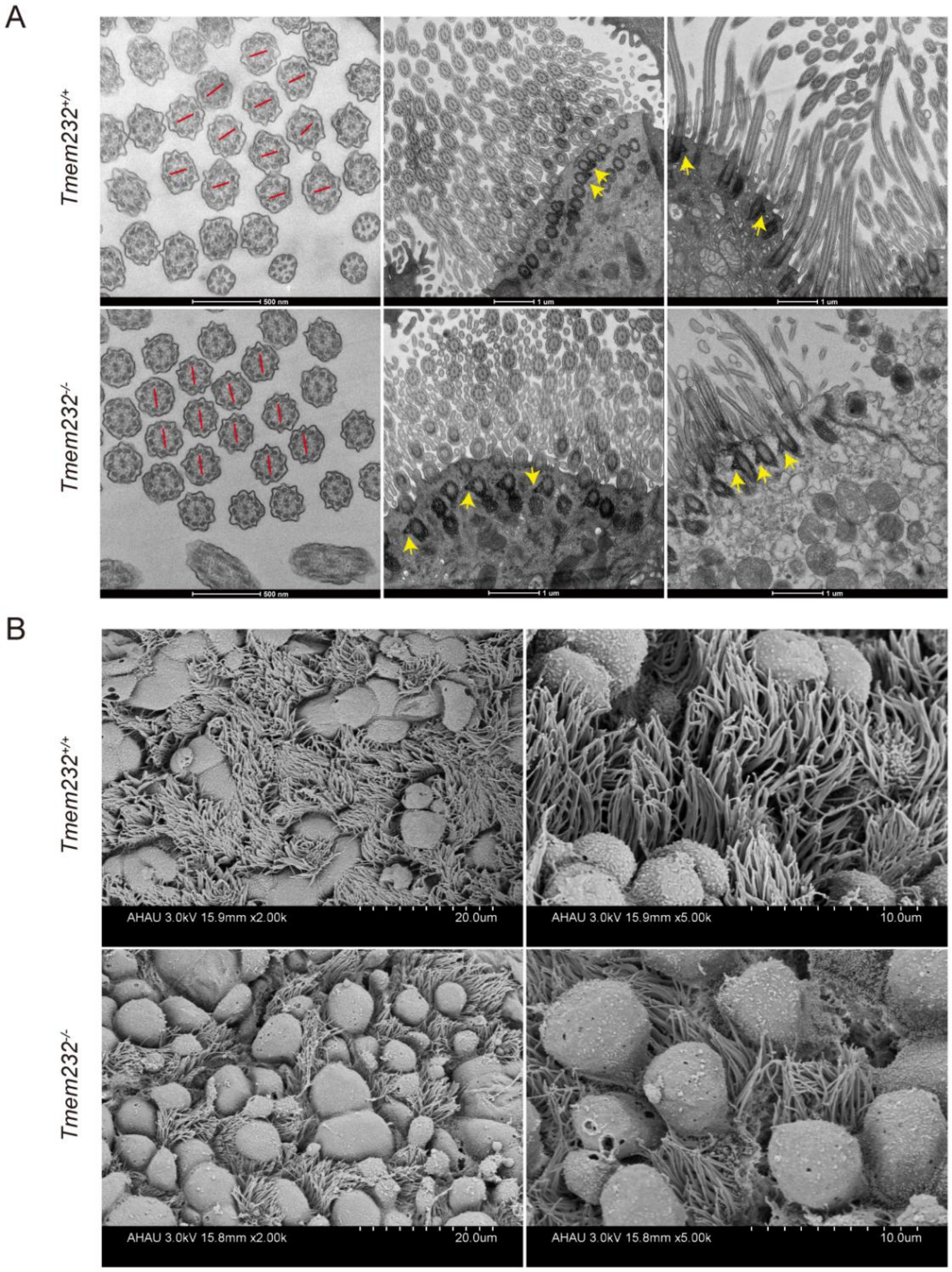

Figure S5. The ultrastructure of respiratory cilia in *Tmem232*$^{+/+}$ and *Tmem232*$^{-/-}$ mice. (A) TEM analysis revealed normal "9 + 2" structure and coordinated directionality of axonemes in *Tmem232*$^{-/-}$ mice. The rotational polarity of each axoneme was evaluated by the angle of the red line connecting the central pair. The arrangement of basal bodies of *Tmem232*$^{-/-}$ tracheal cilia was regular and the basal feet (yellow arrow) were normal in both cross section and longitudinal section. (B) SEM analysis revealed shorter and fewer cilia in *Tmem232*$^{-/-}$ tracheal epithelium cells compared with *Tmem232*$^{+/+}$ mice.

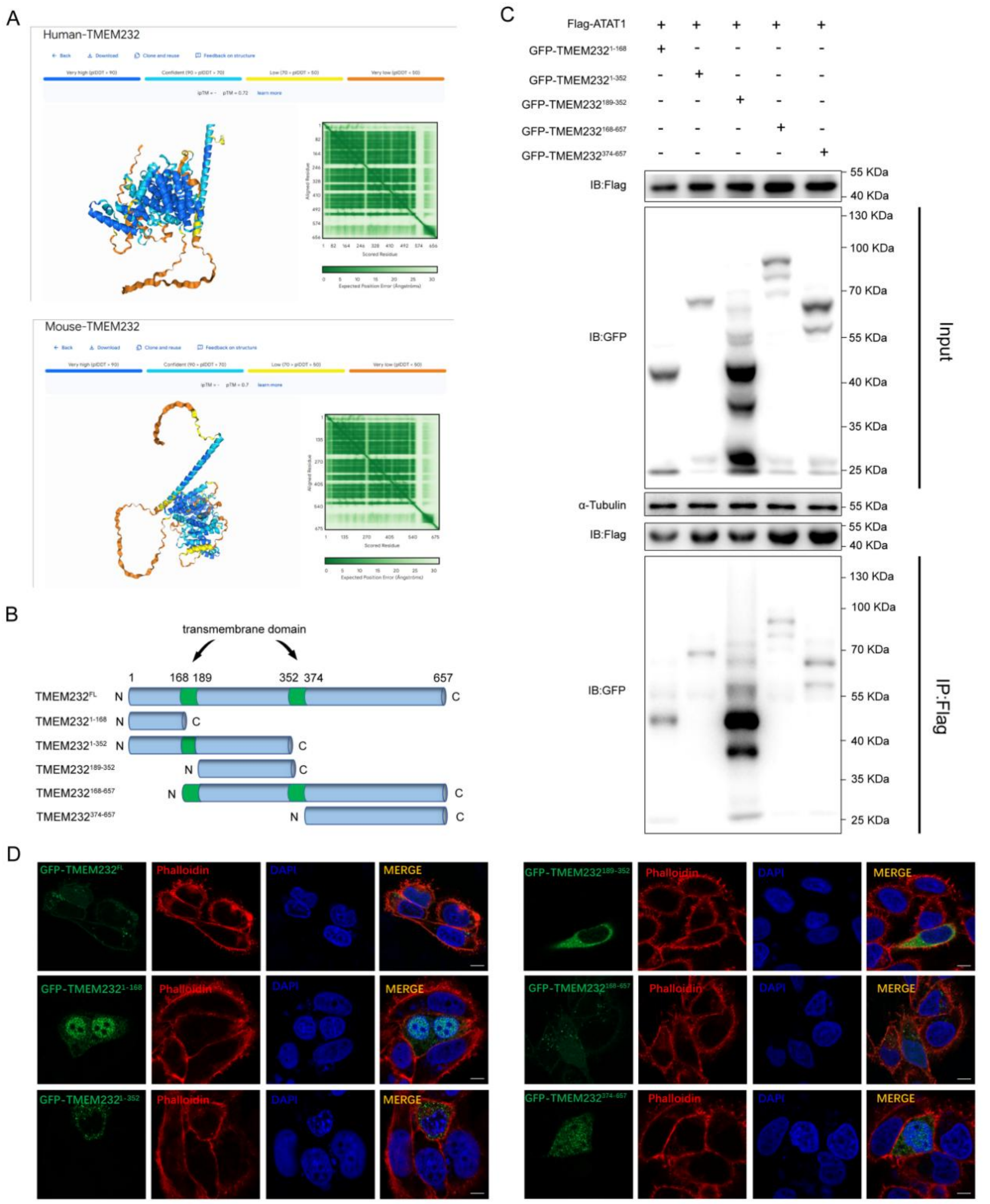

Figure S6. TMEM232 is a typical transmembrane protein of intracellular vesicle. (A) The three-dimensional structure of human and mouse TMEM232 protein. (B) Schematic diagram showing multiple different segments of TMEM232 protein. (C) Western blotting analysis of TMEM232 and its truncated mutants. The representative image of biological duplicates is shown. Immunoprecipitation experiments were performed using FLAG-M2 beads and the lysates of HEK293T cells co-transfected GFP-TMEM232^{FL}, GFP-TMEM232$^{1\text{-}168}$, GFP-TMEM232$^{1\text{-}352}$, GFP-TMEM232$^{189\text{-}352}$,

GFP-TMEM232$^{168-657}$, GFP-TMEM232$^{374-657}$, with FLAG-ATAT1, respectively. The isolated proteins were then analyzed via western blotting with anti-FLAG and anti-GFP antibodies. The representative image of biological duplicates is shown. (D) Immunofluorescent staining of TMEM232 and its truncated mutant (green), phalloidin (F-actin, red), and DAPI (Nucleus, blue) in HeLa cells. Scale bar:10 μm.

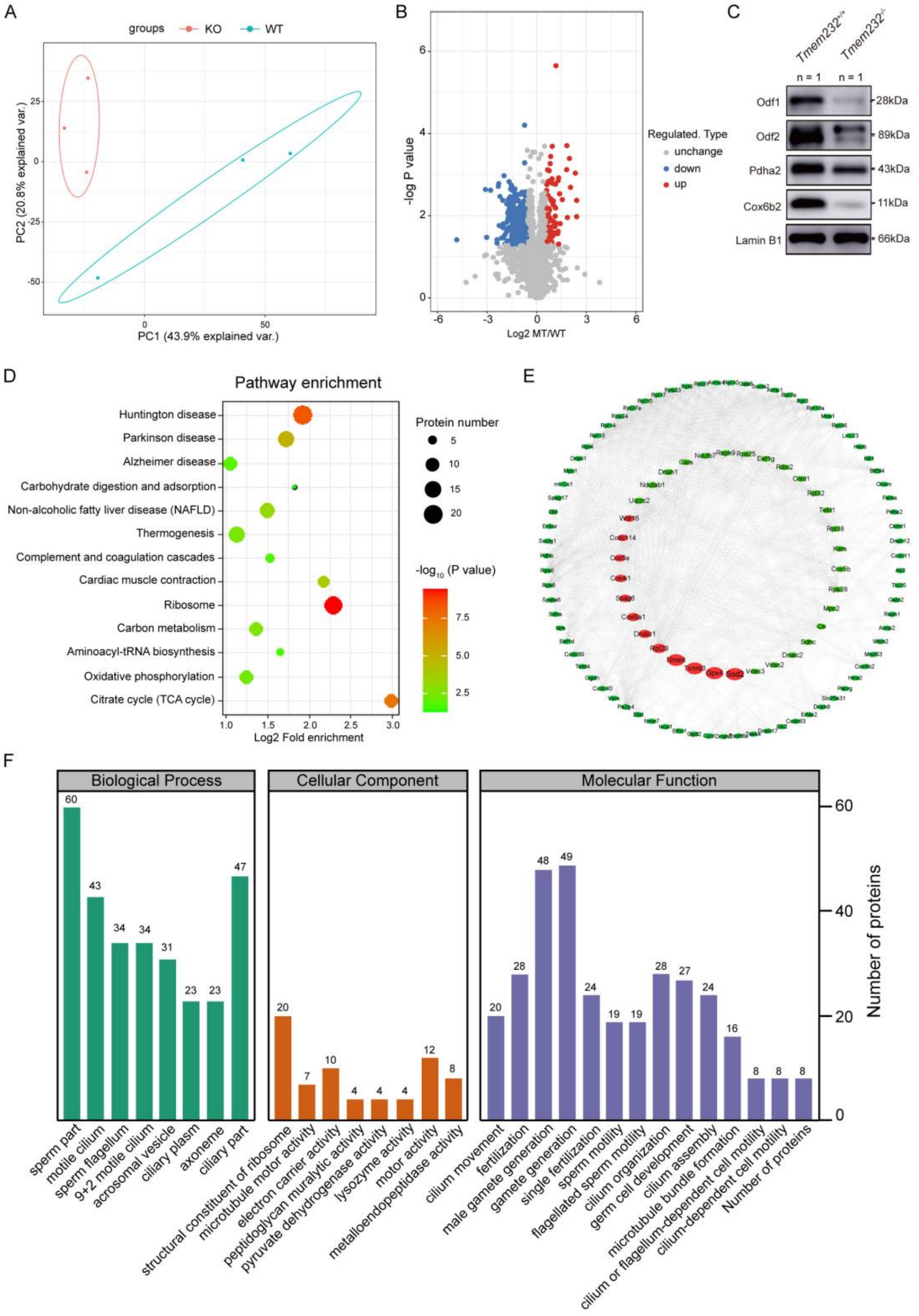

Figure S7. Proteomic identification and bioinformatic analysis of sperms from *Tmem232*$^{-/-}$ and wild-type mice. (A) Principal component analysis (PCA). The 2D plot of PCA between *Tmem232*$^{-/-}$ and wild-type mouse sperm samples. Differentially expressed proteins (DEPs) were determined based on a threshold of FC (fold change) > 1.5 and $P < 0.05$. PCA1 and PCA2 represent the first and second principal components, respectively. (B) Volcano plot of DEPs. Volcano plots showing differentially enriched proteins between *Tmem232*$^{-/-}$ (n = 3) and wild-type mouse

sperms (n = 3). Red symbols ($P < 0.05$ and >1.5-FC) represent upregulated proteins enriched in the *Tmem232*$^{-/-}$ mouses sperms, blue points indicate downregulation, and gray points indicate no change. For group-wise comparisons, see Table S3. (C) Western blotting confirmed the DEPs identified via proteomic analysis of *Tmem232*$^{-/-}$ and *Tmem232*$^{+/+}$mouse sperm. Lamin B1 served as a loading control. The representative image of biological duplicates is shown. (D) KEGG enrichment of differentially abundant proteins. The scatter plot of 13 significantly enriched KEGG pathways based on the DEPs between *Tmem232*$^{-/-}$ and wild-type mouse sperms. The y-axis indicates different KEGG pathways. The x-axis indicates the enrichment factors, calculated using log2 (fold enrichment), that is, the extent to which differentially abundant proteins are enriched in the pathway. The color of the circles indicates the P-value of the pathway ($P < 0.05$). The size of the circles indicates the number of proteins. The significantly enriched pathways are shown in this figure. (E) Protein-protein interaction network and the hub gene module of the overlapping DEPs. Red circles indicate the most significant module selected from MCODE in Cytoscape. The larger the circle, the higher the interaction degree identified. (F) Significant GO terms of DEPs between *Tmem232*$^{-/-}$and wild-type mouse sperm. The x-axis indicates different GO terms. The y-axis indicates the number of DEPs enriched in the pathway.

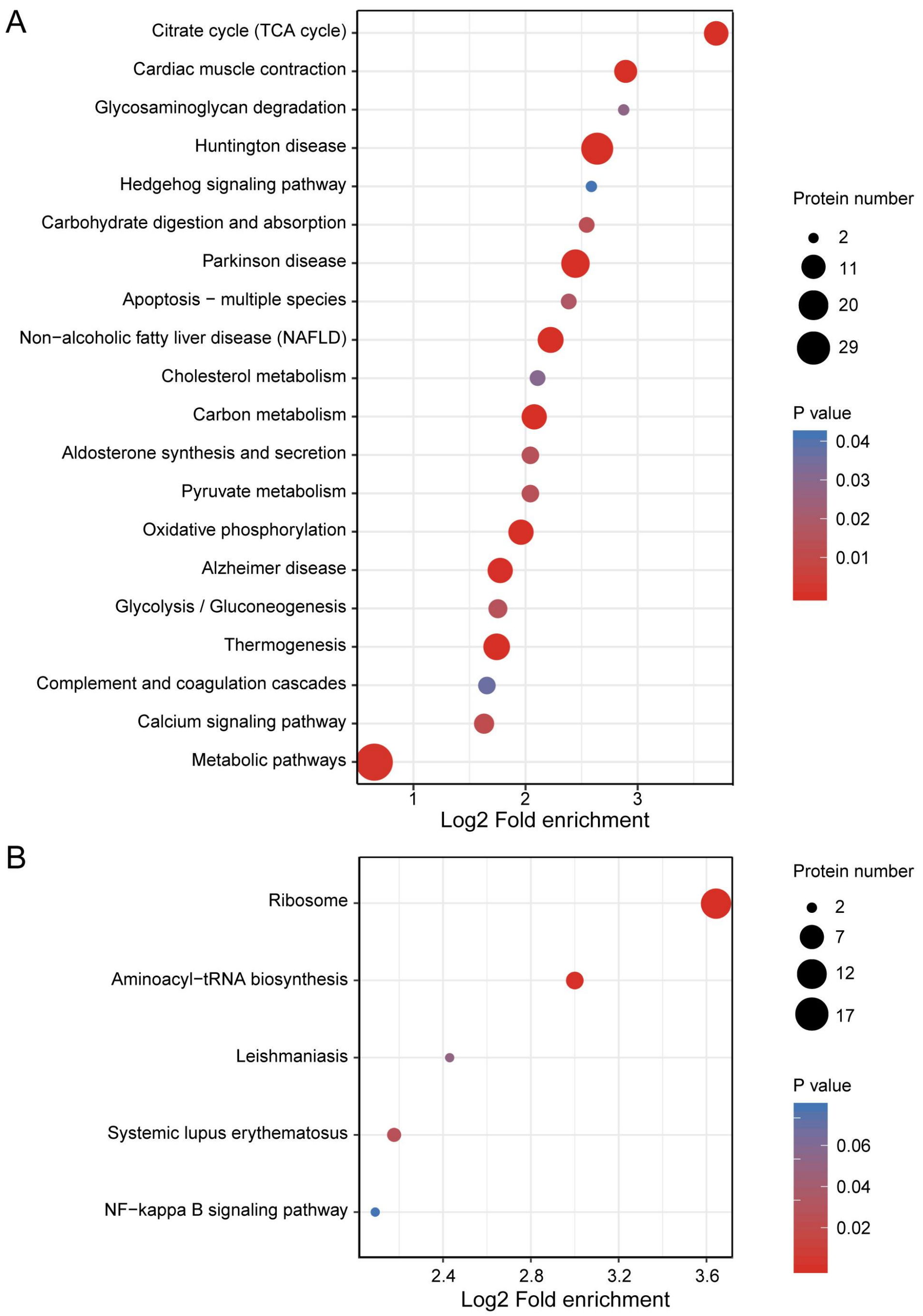

Figure S8. The KEGG enrichment analysis of the (A) 255 downregulated and (B) 88 upregulated differentially expressed proteins, respectively. The y-axis indicates different KEGG pathways. The x-axis indicates the rich factors, calculated by log2 (Fold enrichment), which represents the extent of differentially expressed proteins enriched in the pathway. The color of the circles indicates the *P*-value of the pathway ($P < 0.05$). The size of circles indicates the number of proteins.

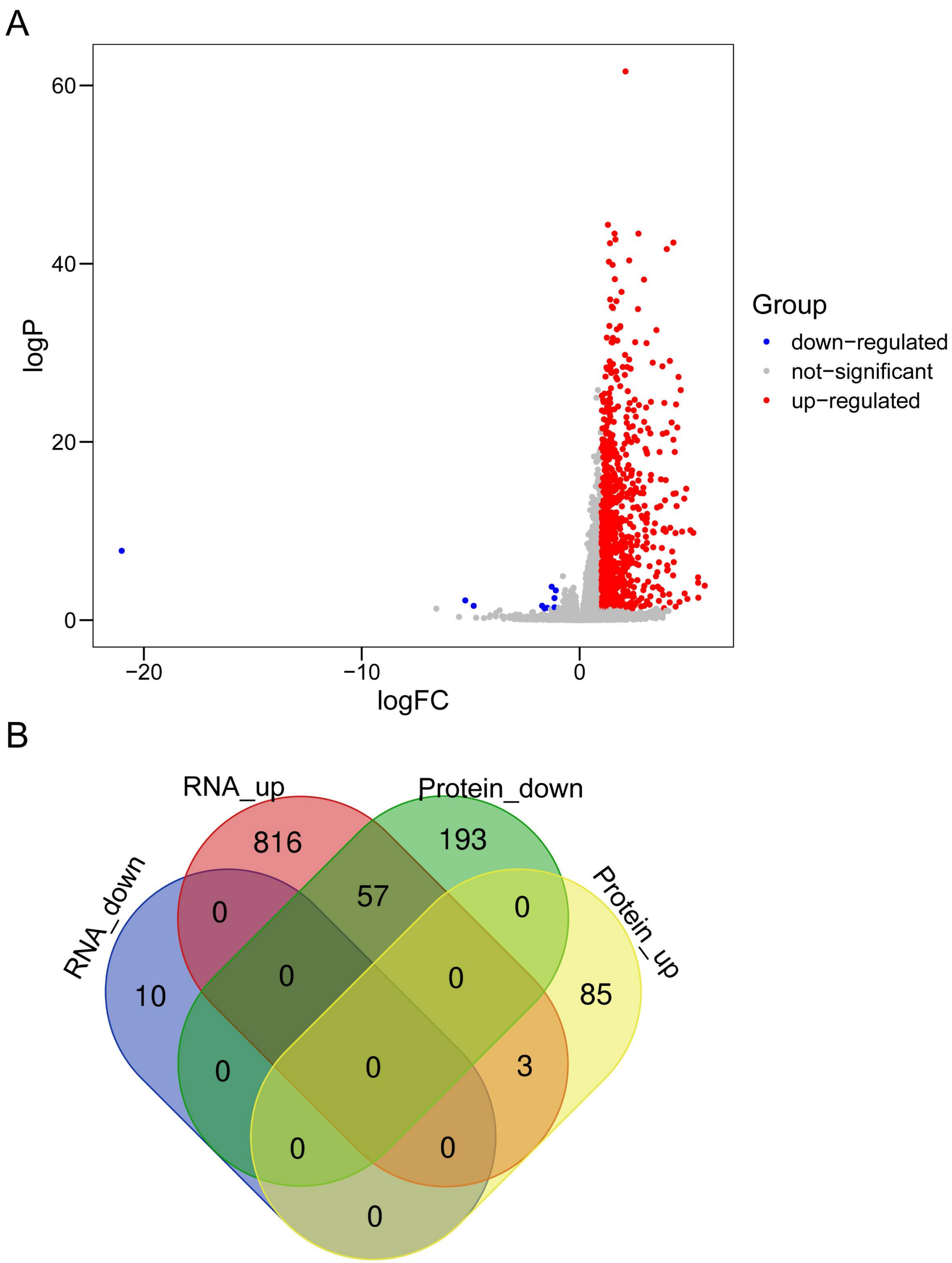

Figure S9. The comparision of differentially expressed genes and proteins between *Tmem232*$^{+/+}$ and *Tmem232*$^{-/-}$ mice. (A) Transcript levels (normalized gene counts) are plotted as a function of differential expression (log2-transformed fold change) between *Tmem232*$^{+/+}$ and *Tmem232*$^{-/-}$ mice testis. Up-regulated expression genes in *Tmem232*$^{-/-}$ mice testis are shown in red and down-regulated expression genes are shown in blue. (B) Venn diagram showing the overlapping symbols among down-regulated expression genes, up-regulated expression genes, down-regulated proteins and up-regulated proteins identified from *Tmem232*$^{+/+}$ and *Tmem232*$^{-/-}$ mice testis and sperm.
