# Supplementary figures and images for: TMEM232 is required for the formation of sperm flagellum and male fertility in mice
Source: Cell Death Dis. 2024 Nov 8;15(11):806. doi: 10.1038/s41419-024-07200-9 (PMC11549365; doi:10.1038/s41419-024-07200-9)

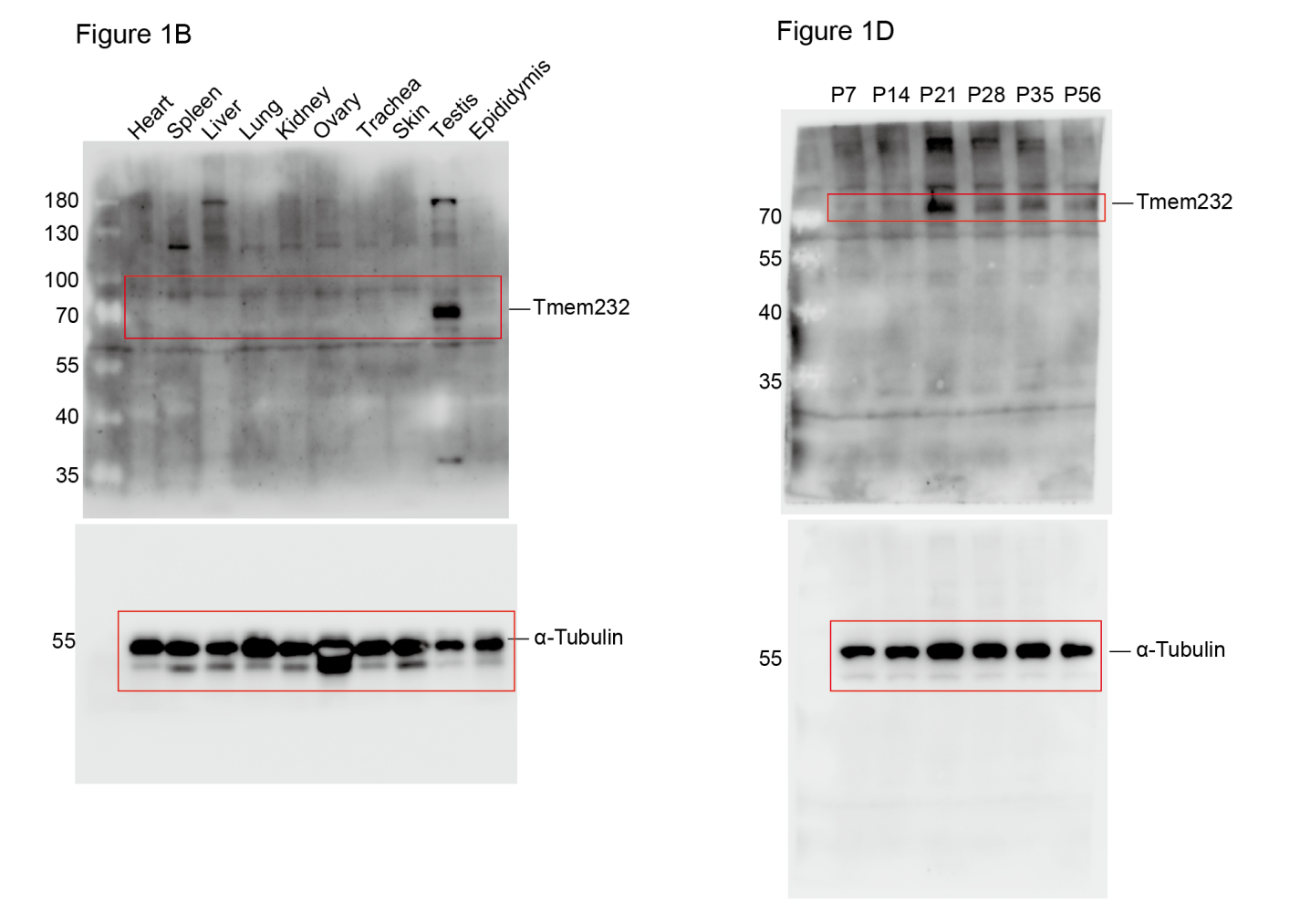


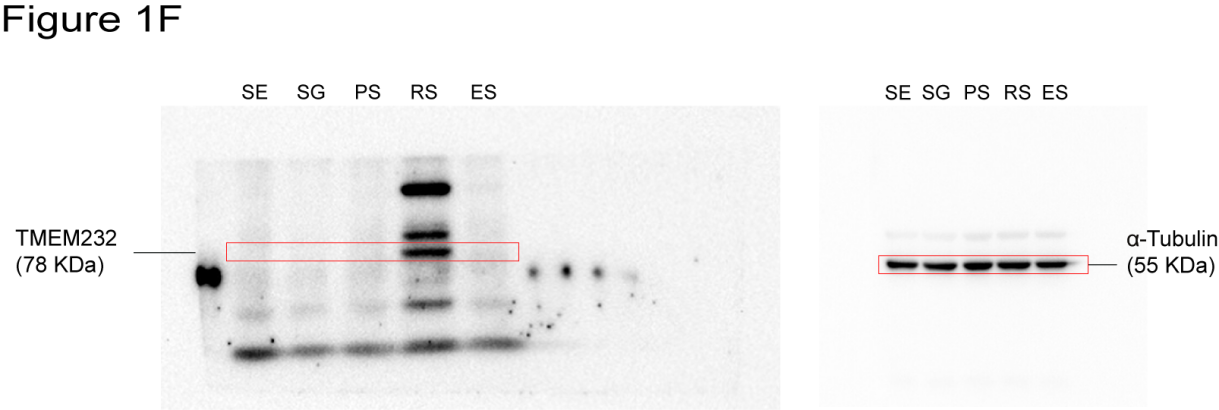





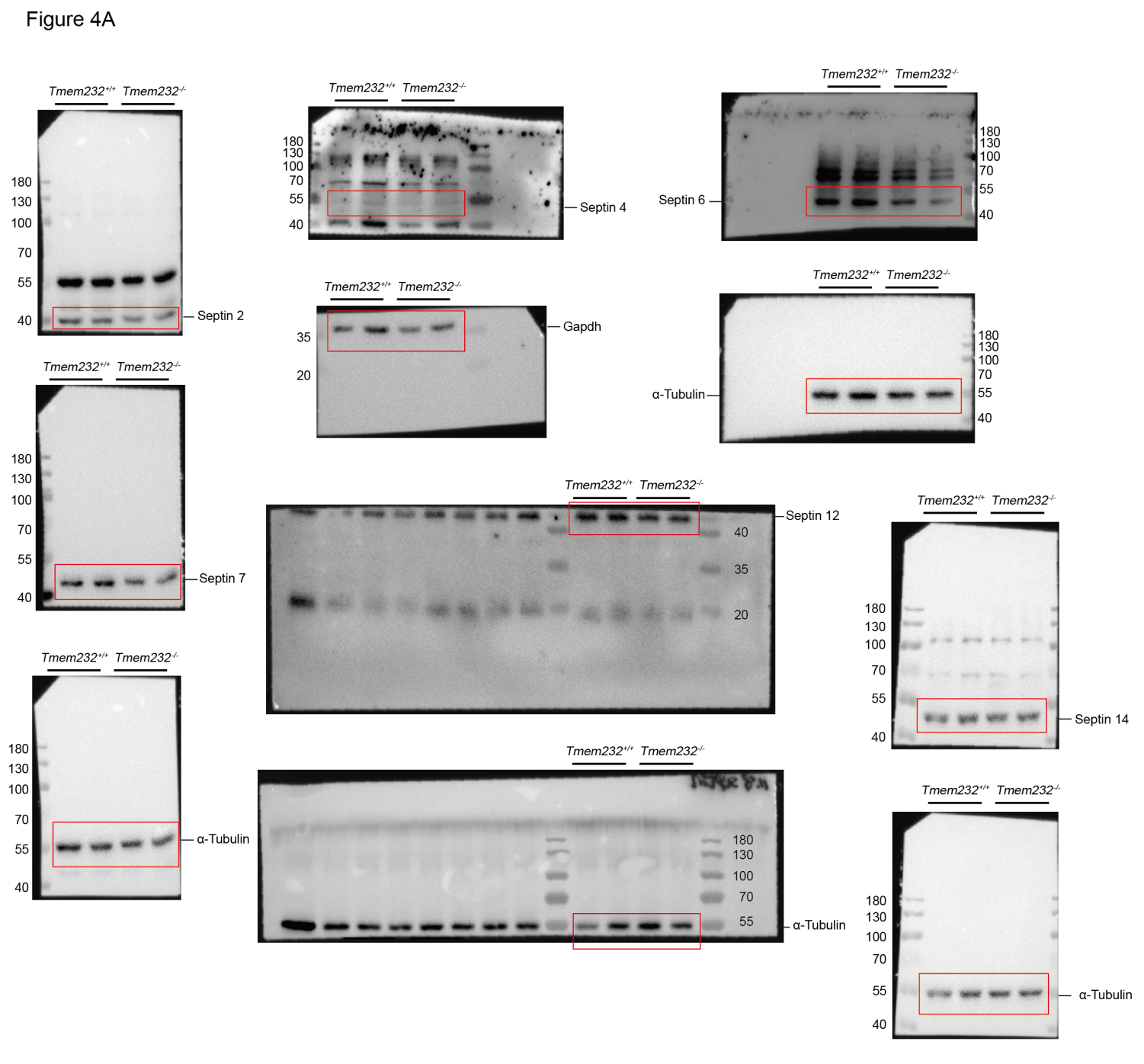

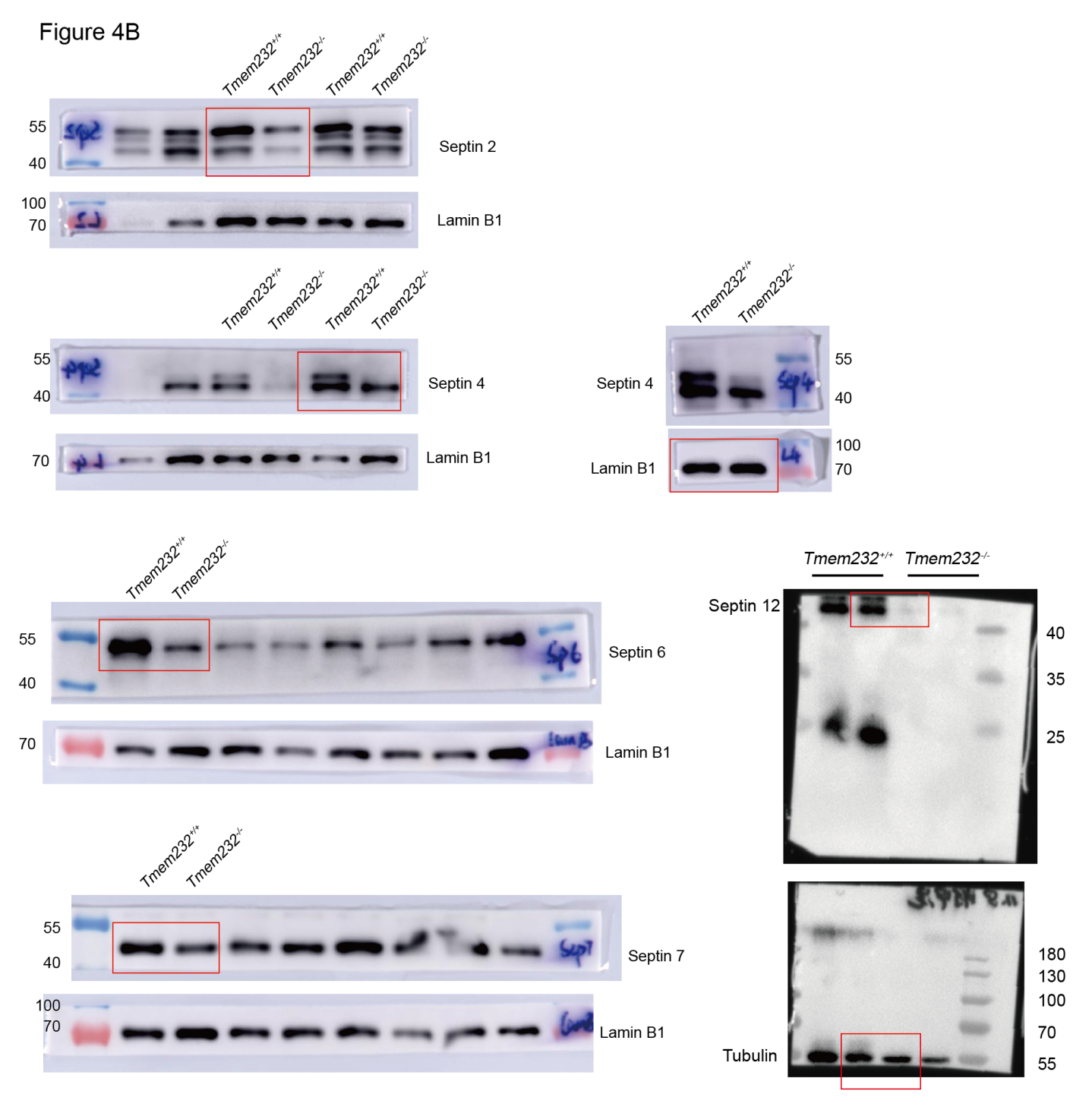

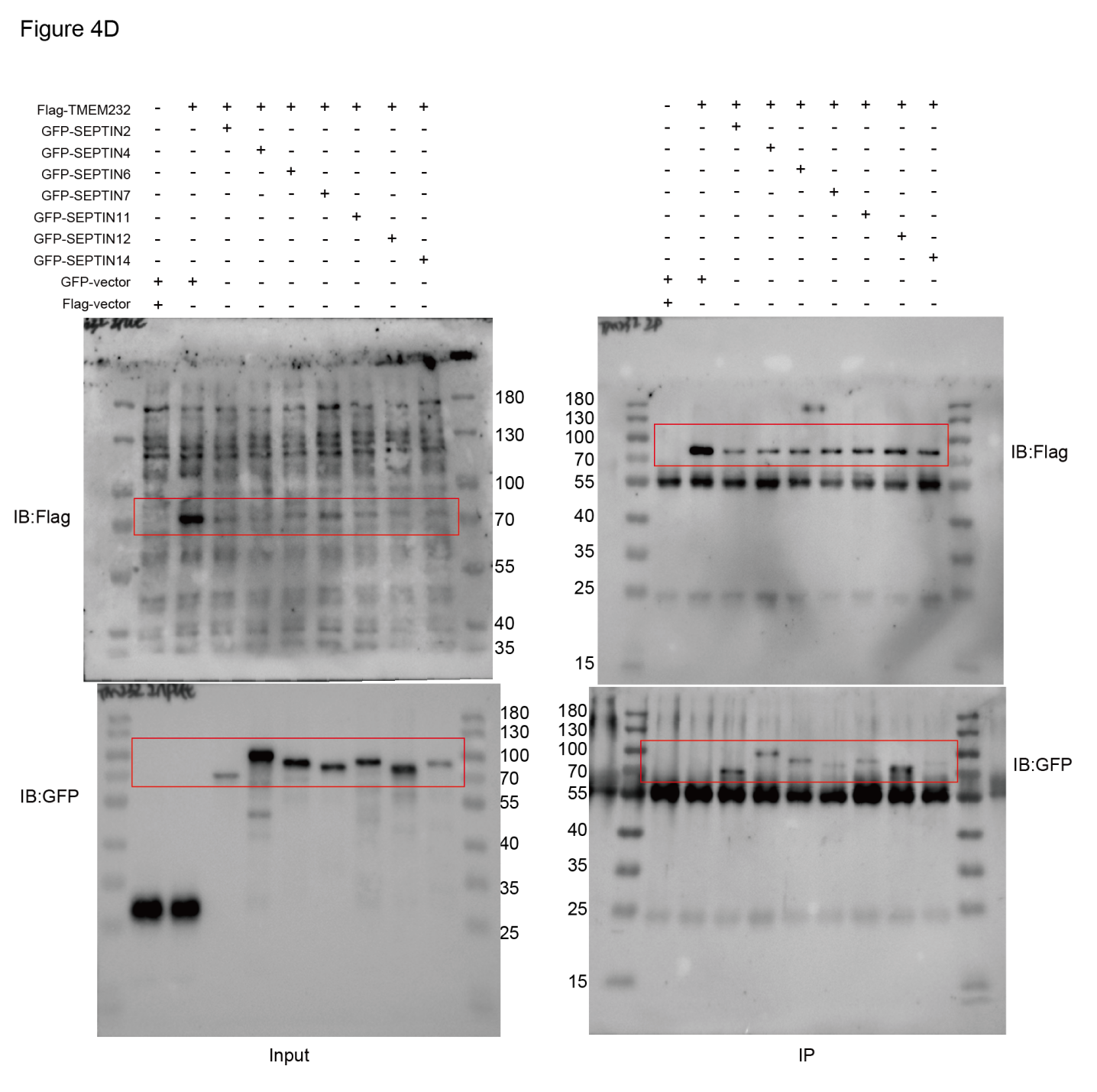

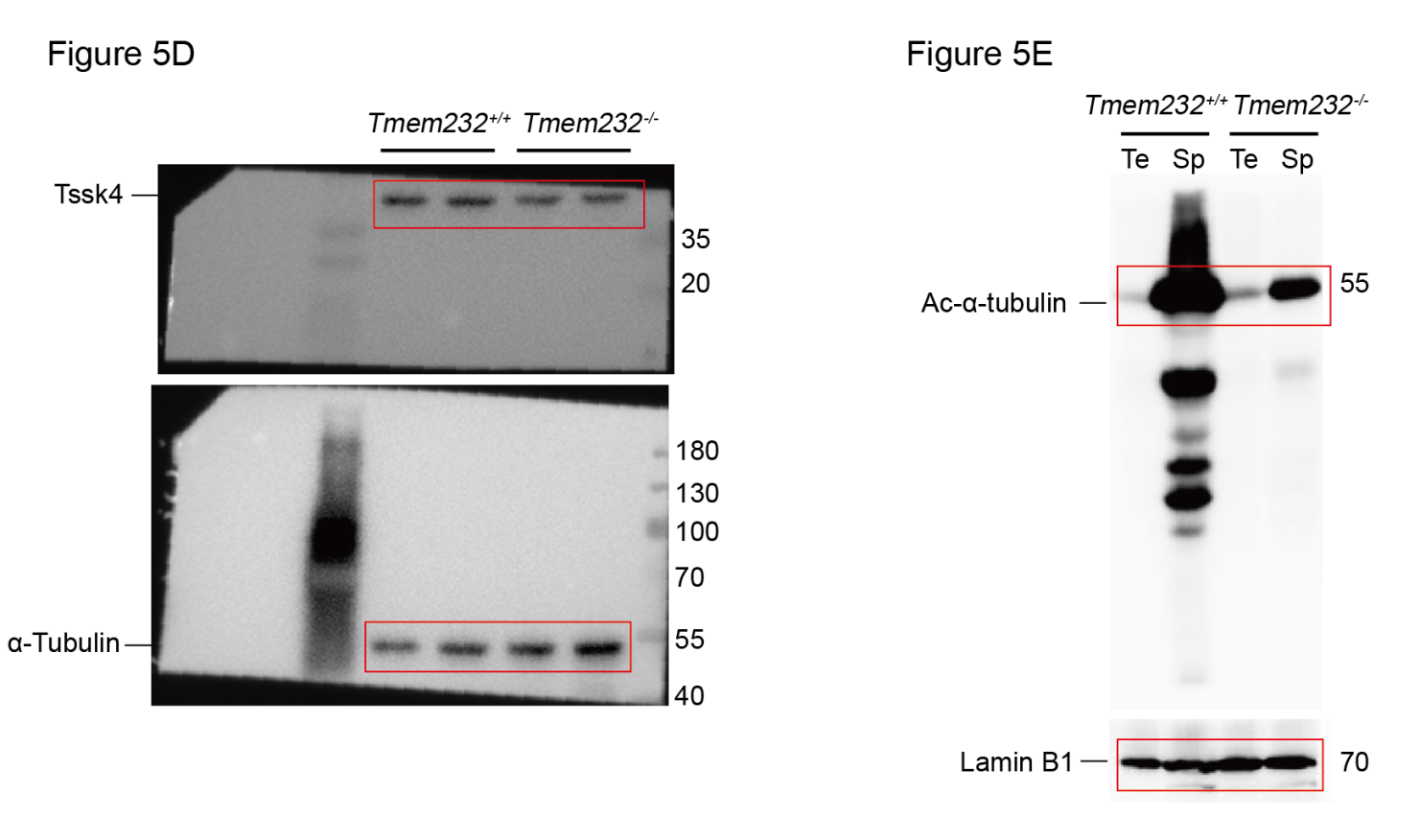


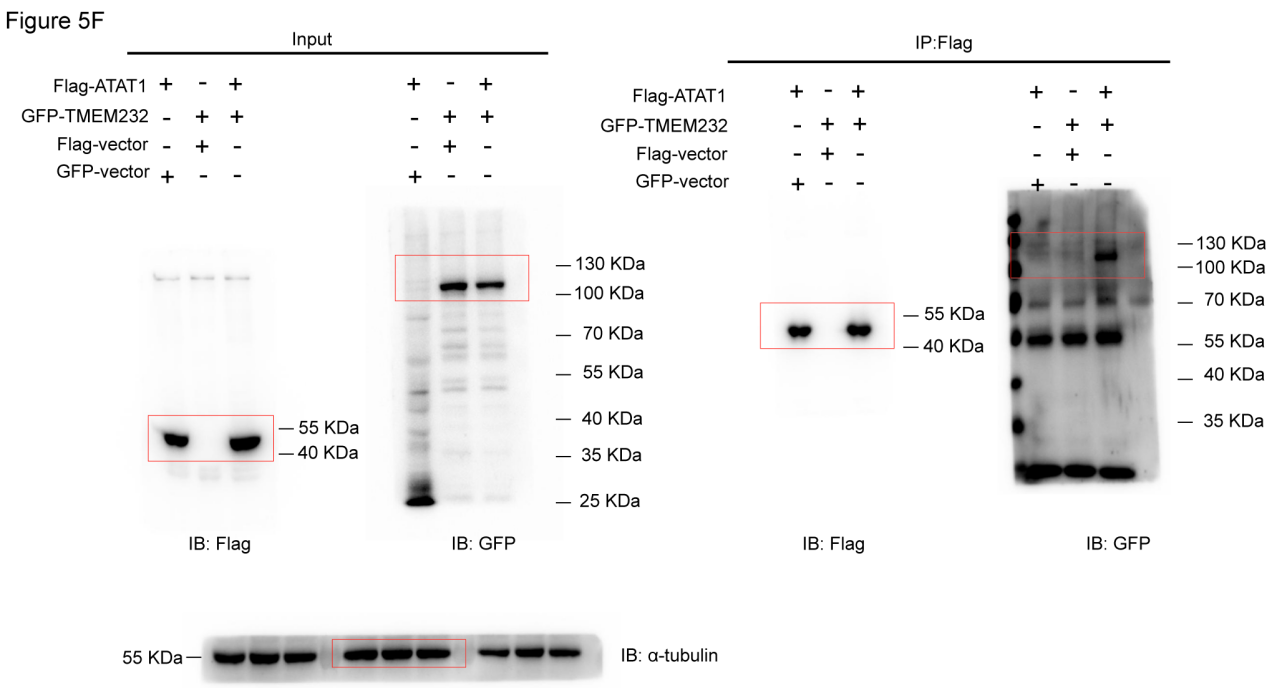


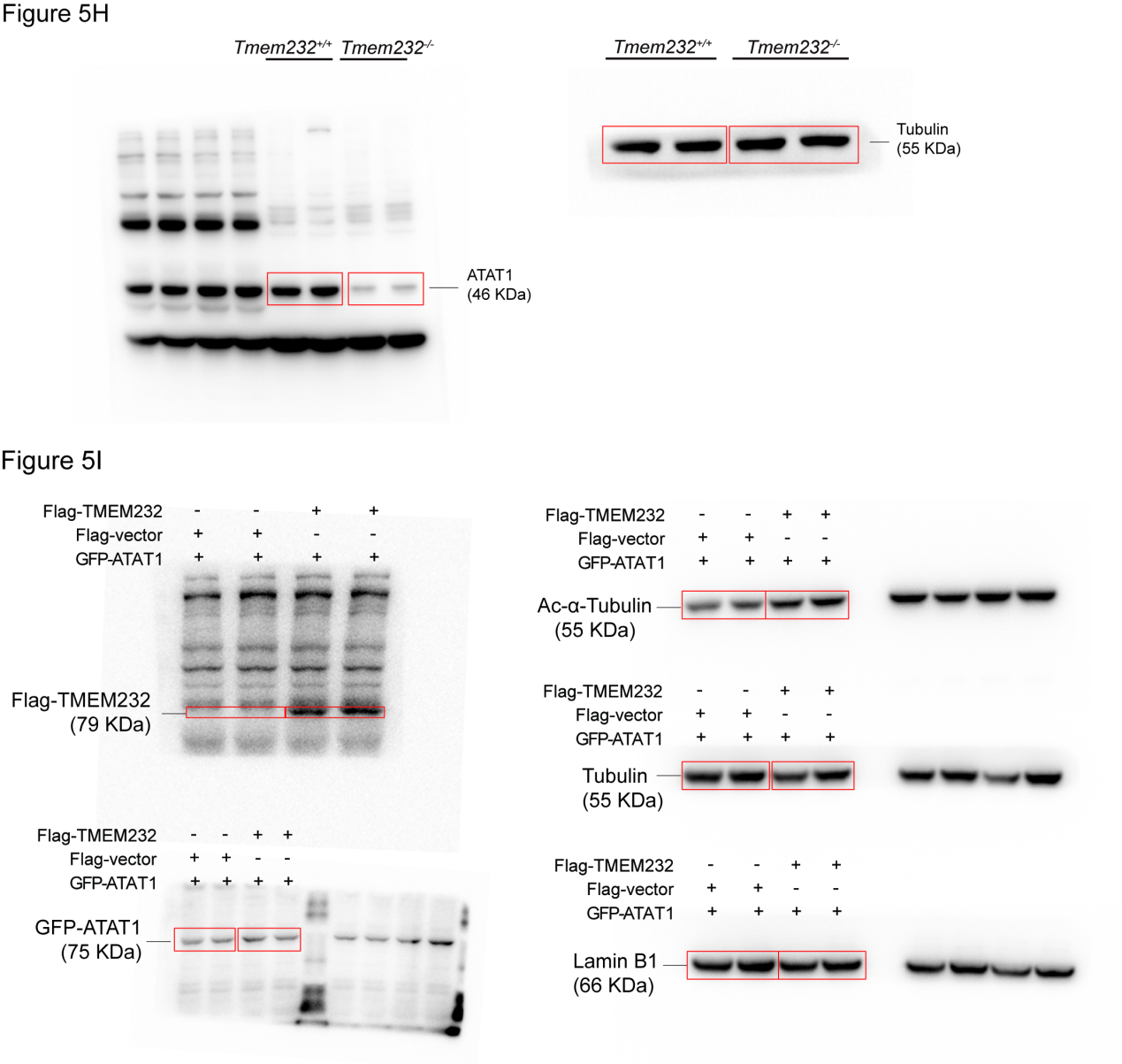


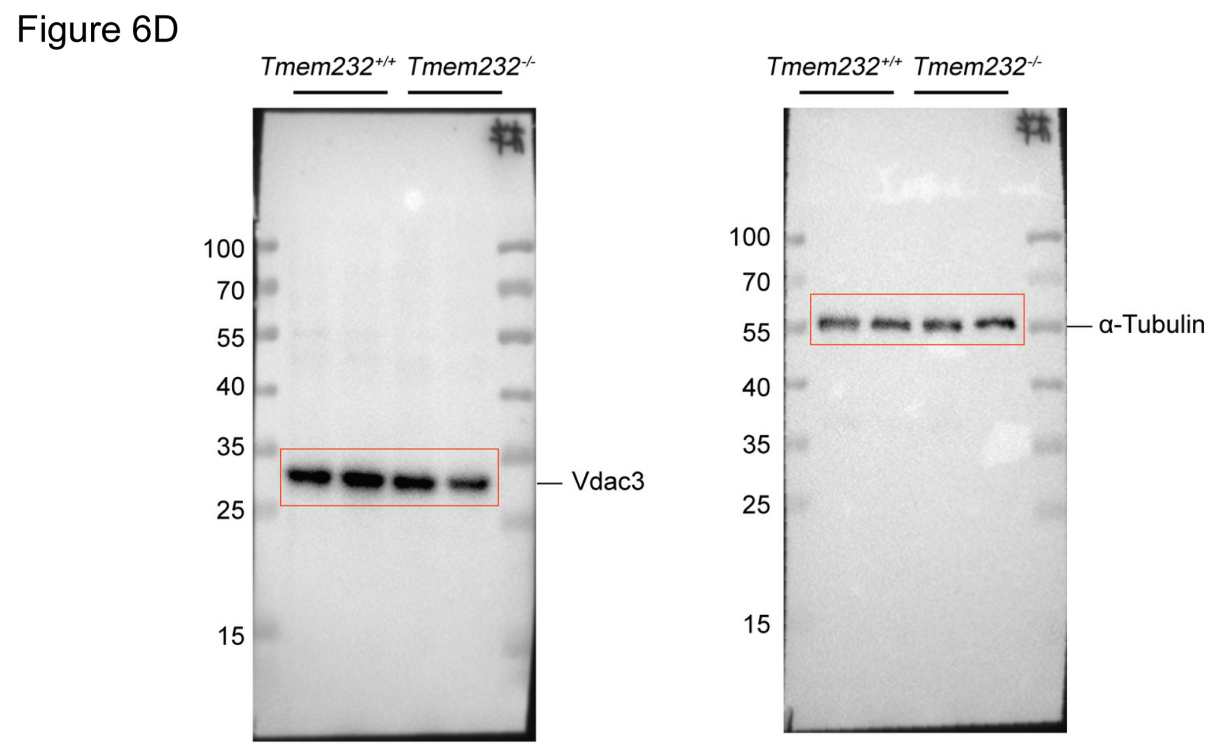


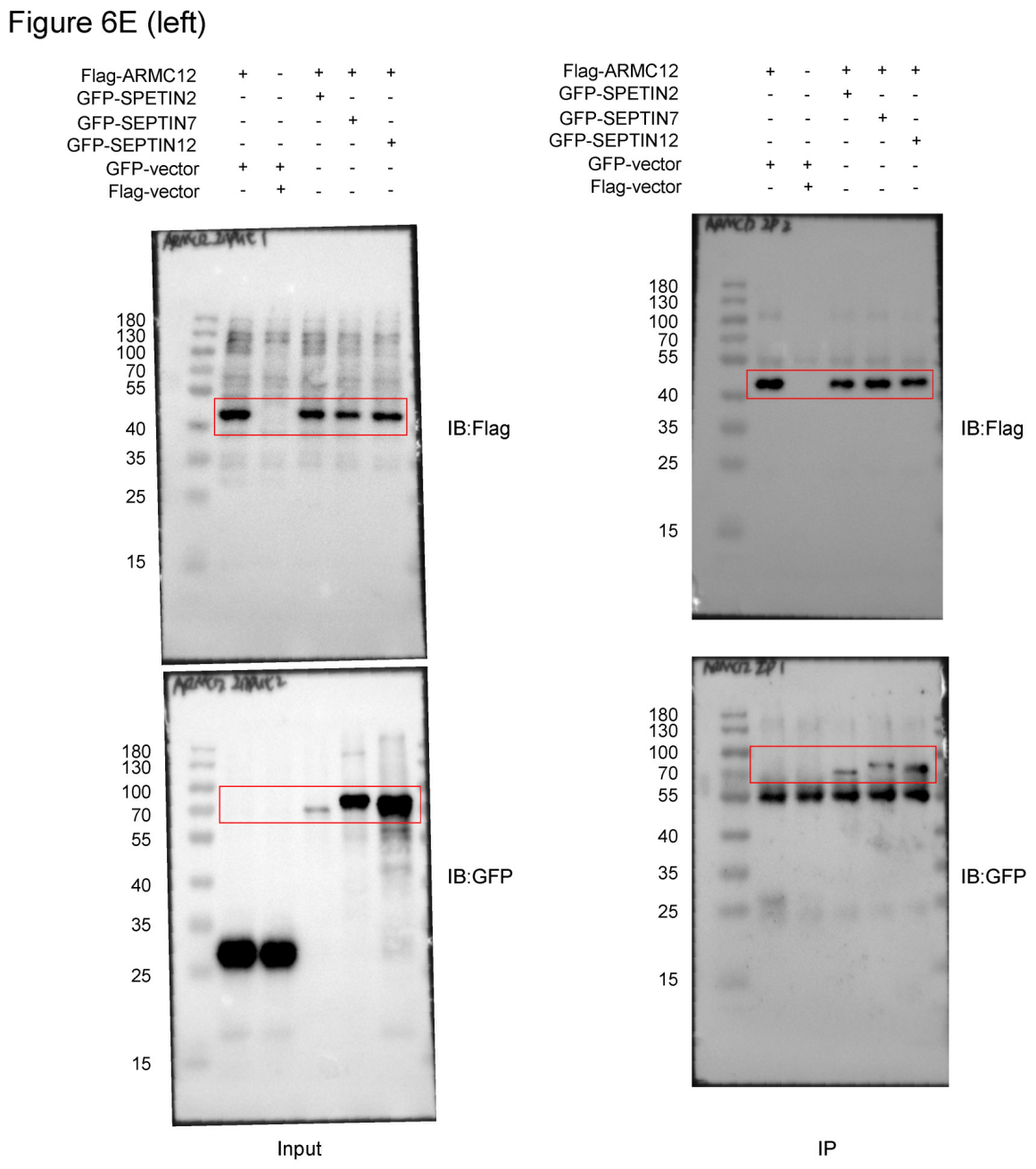





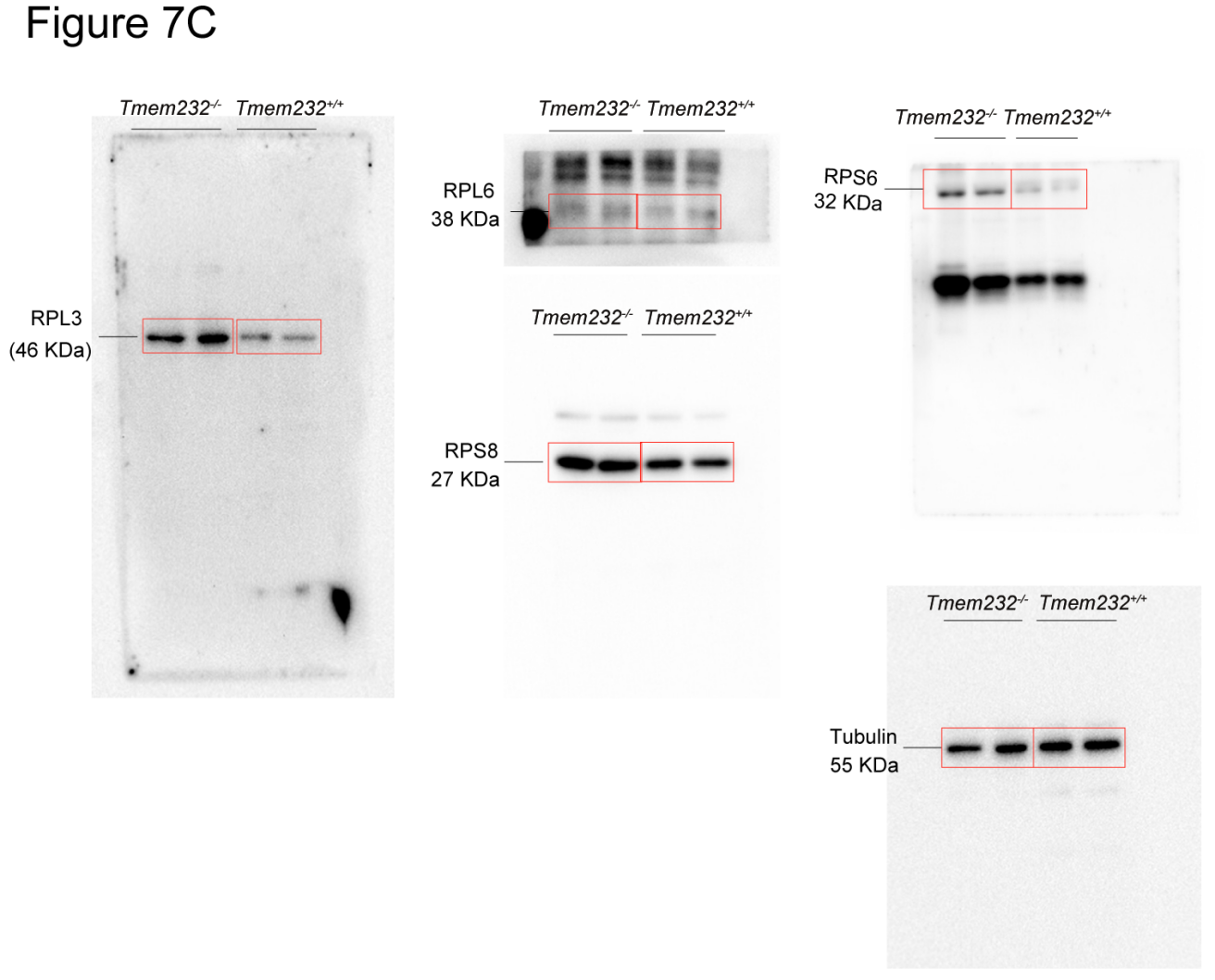


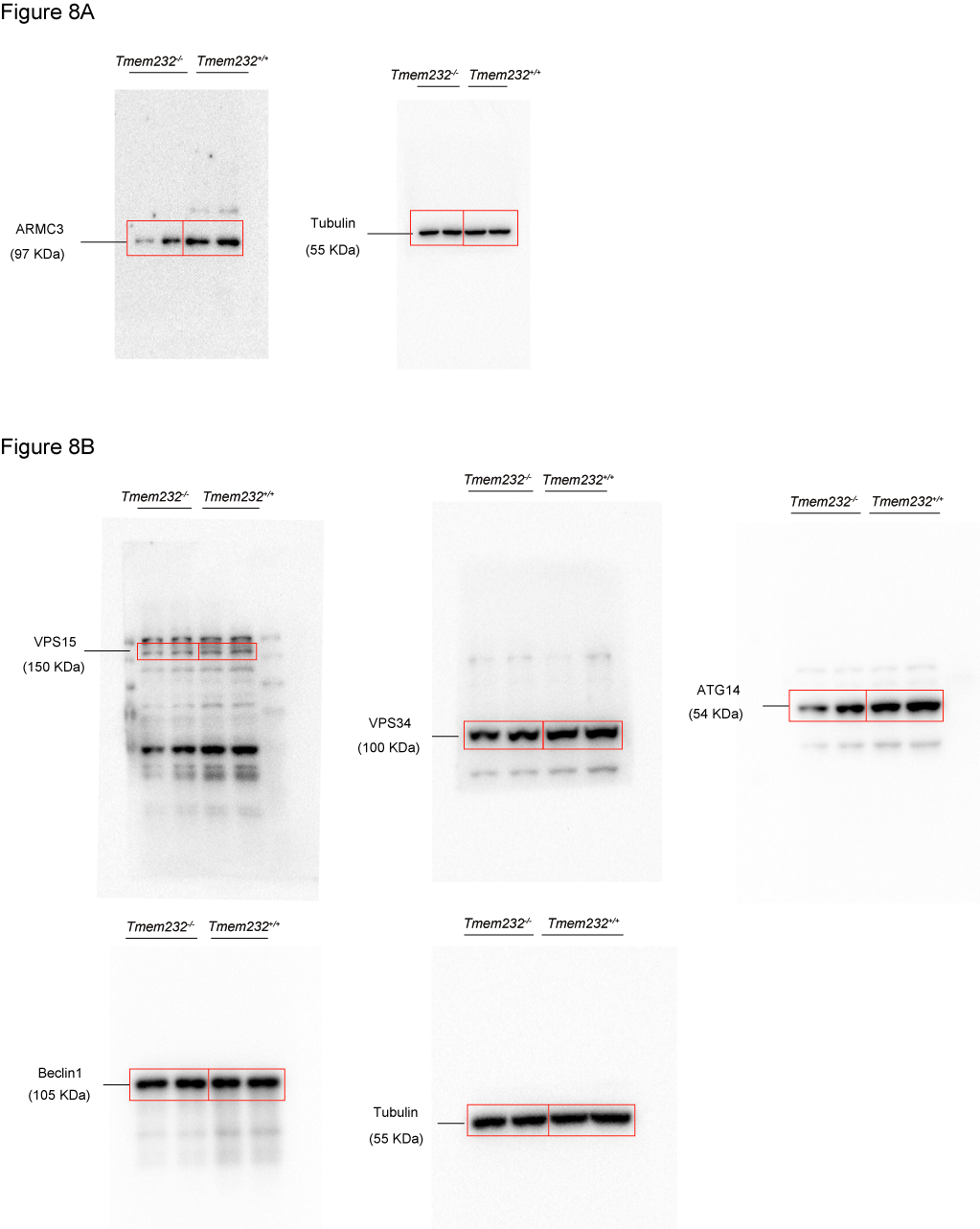

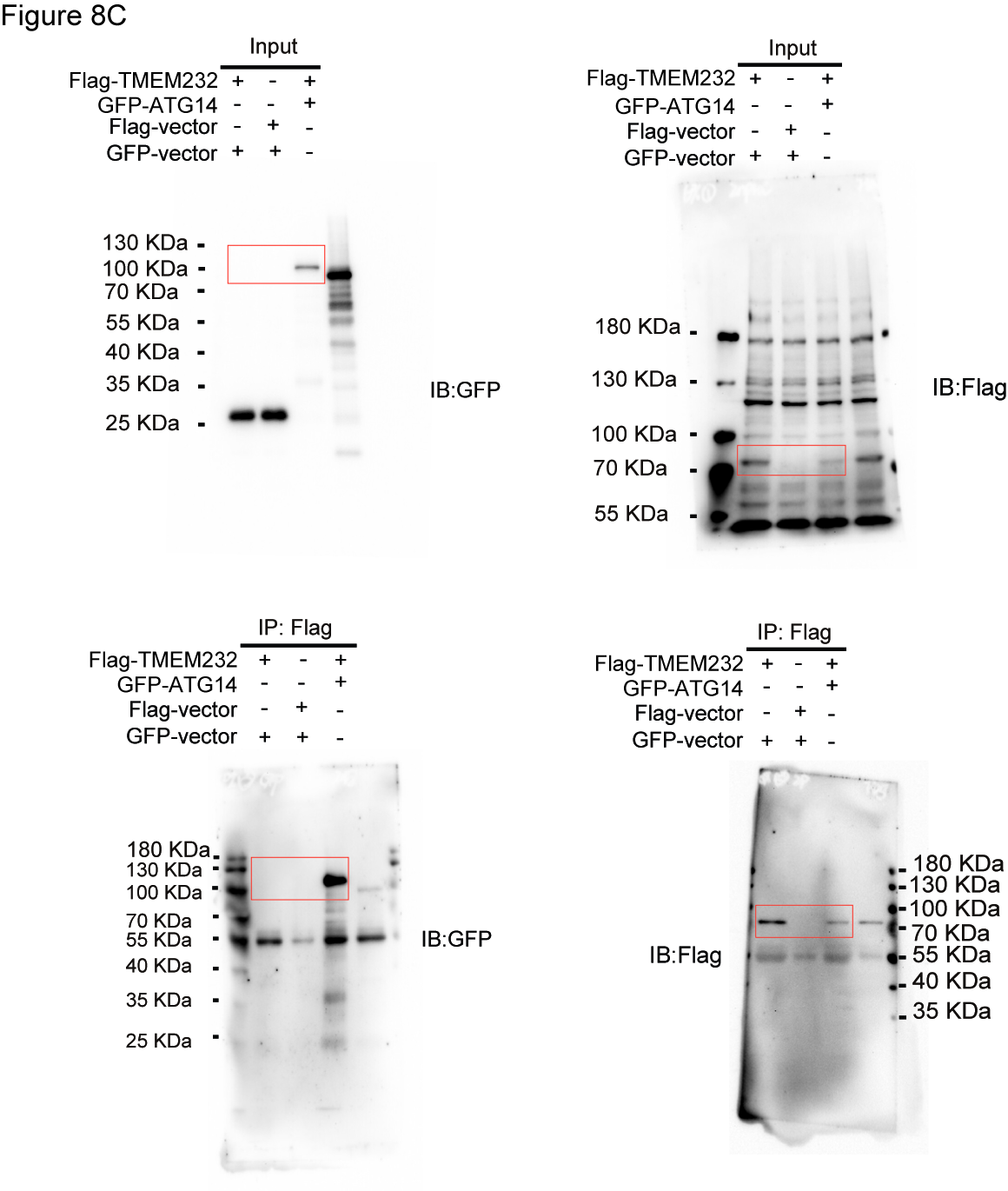

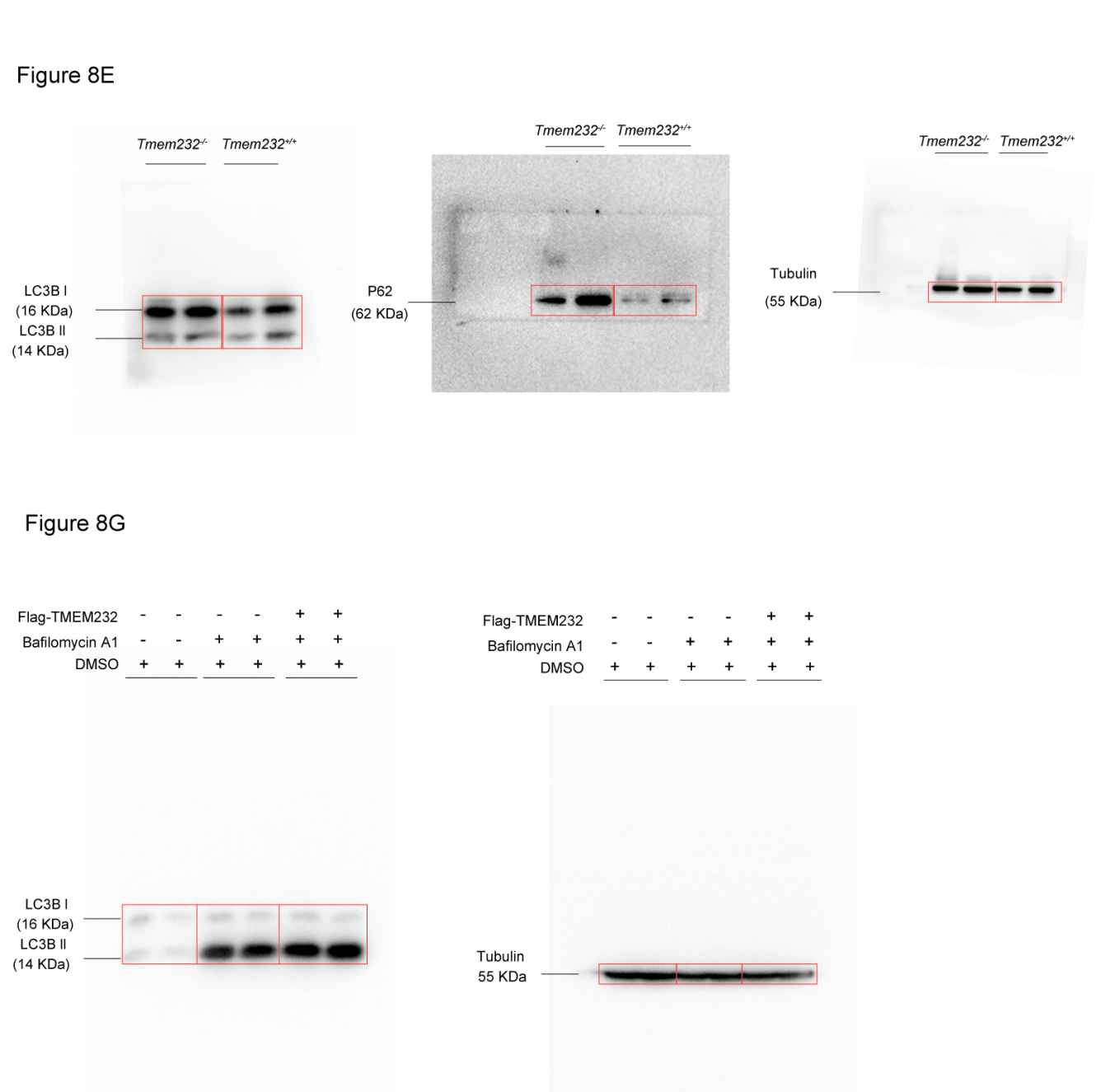


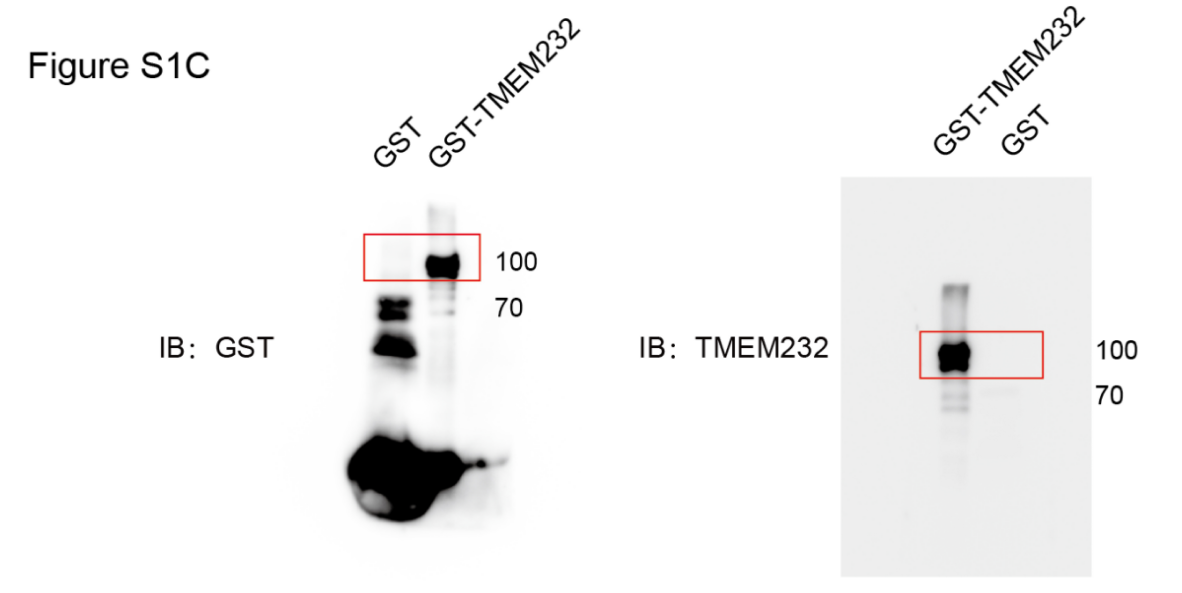


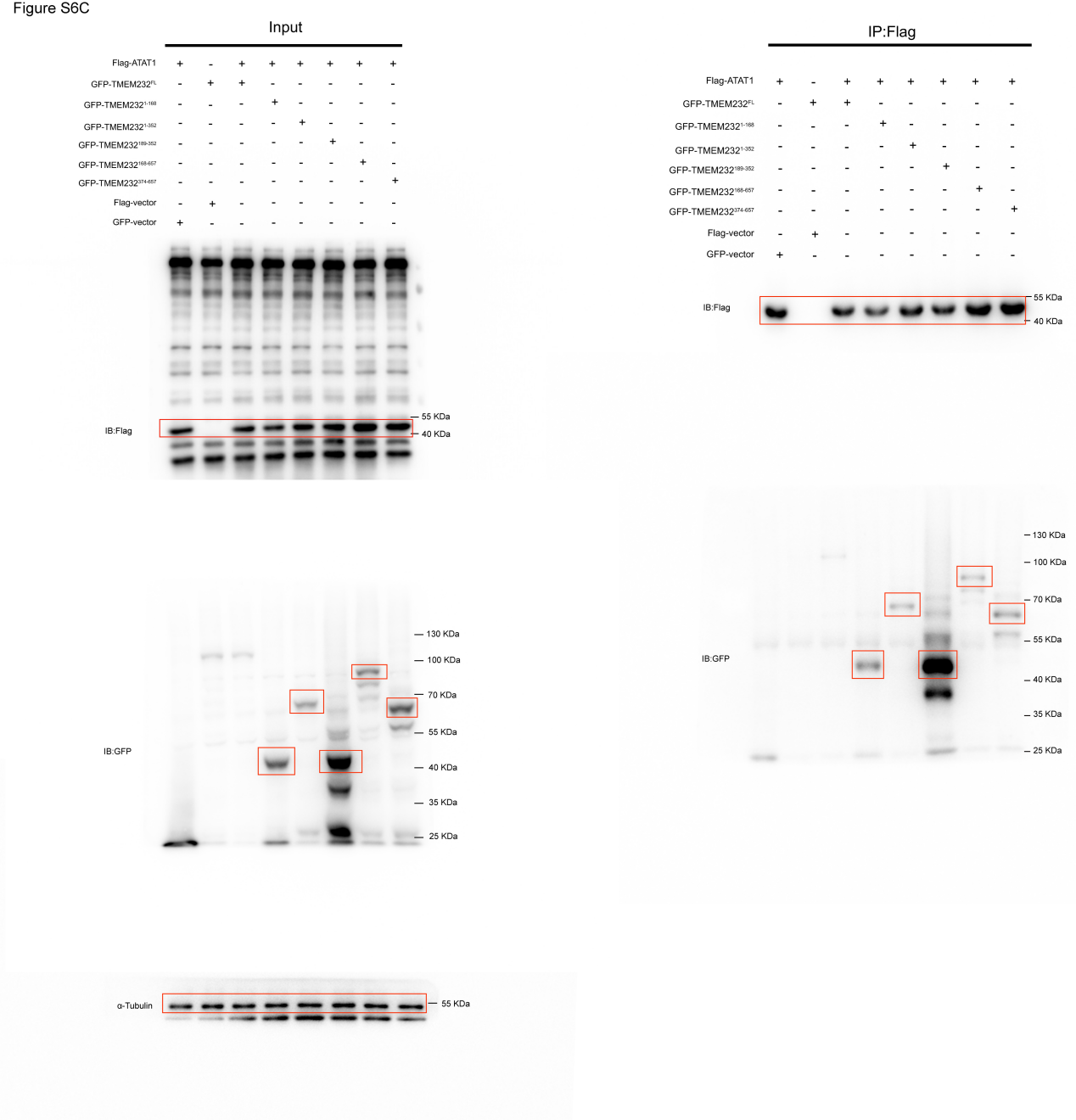


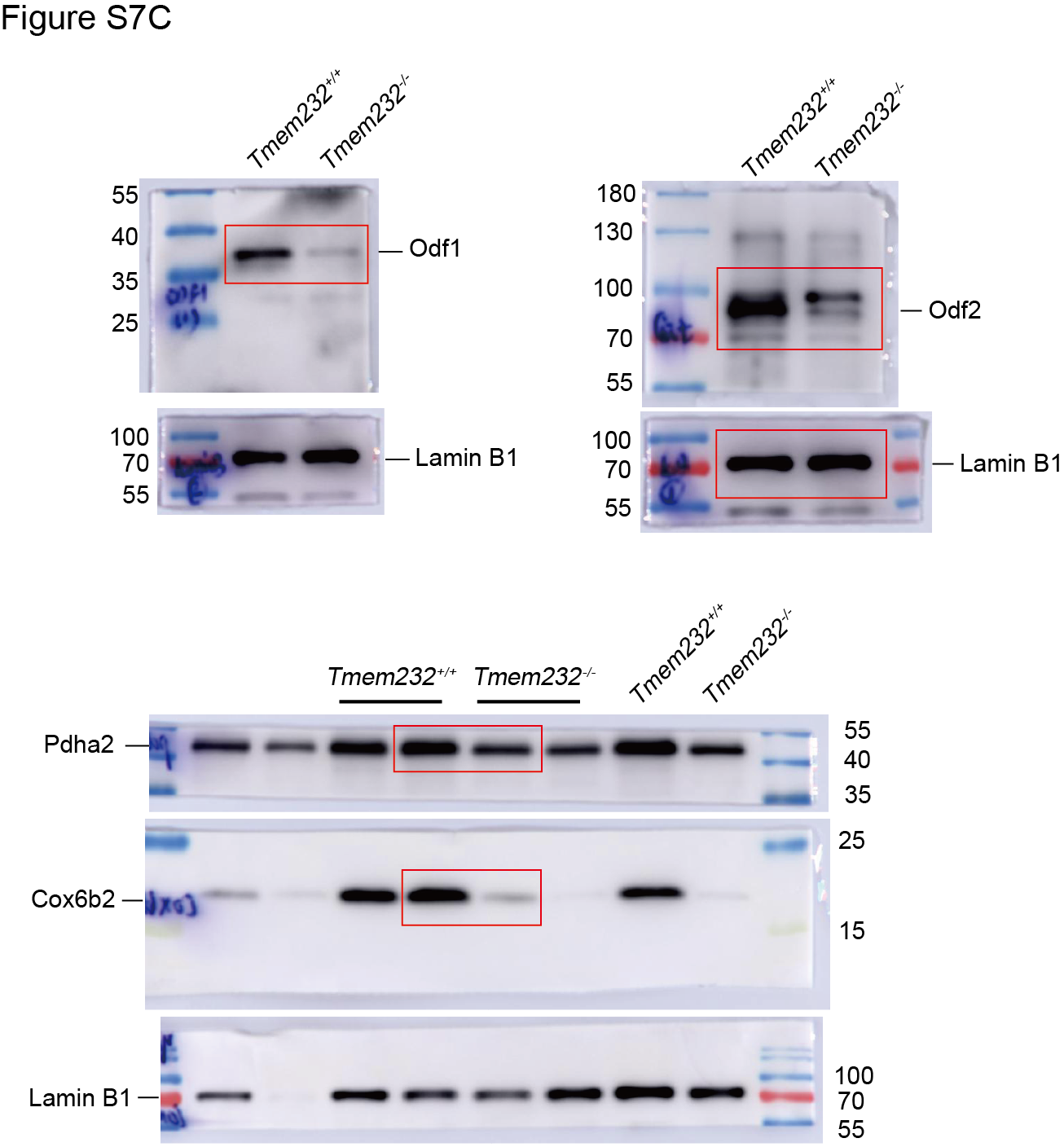

Supplement: Supplementary file 7 — Related Manuscript File [file 41419_2024_7200_MOESM7_ESM.docx]
